# Supplementary material for: Staff perceptions towards virtual reality-motivated treadmill exercise for care home residents: a qualitative feedback study with key stakeholders and follow-up interview with technology developer
Source: BMJ Open. 2023 Nov 23;13(11):e073307. doi: 10.1136/bmjopen-2023-073307 (PMC10668234; doi:10.1136/bmjopen-2023-073307)
Supplement: Supplementary data [file bmjopen-2023-073307supp001.pdf]

Appendices

Appendix A –Workshop Interview Schedule

Site information

1) Please briefly describe the site you work at? (e.g. care home, extra care, supported living)

2) Please describe the number of residents or customers at that site and the level of care required? (e.g. physical ability of residents, prevalence of dementia)

Feedback on ROVR

1) What are your overall thoughts on the ROVR? How physically demanding did you find use of the ROVR?

2) What, if any, benefits do you perceive older adults might gain from using the ROVR?

3) What, if any, challenges might be encountered in the use of the standing or seated ROVR system with older adults (and care home residents)?

4) What characteristics do you think older people would need to use the standing and/or seated ROVR safely?

5) What challenges or health conditions do you think might make use of the standing and/or seated ROVR unsafe? (e.g. visual impairments, osteoarthritis, balance and coordination issues, frailty, mobility issues, support with grooming/communication/transfers/toileting/locomotion/self-care/communication *examples taken from the Functional Independence Measure*)

6) If the ROVR was in your care facility, do you envisage that it would change ways of working? (if so, how?)

7) Based on the video and demonstration you saw today, would you feel confident supporting use of the ROVR at your care facility? (further ideas on training requirements)

Appendix B – Supporting evidence for Table 2

Theme	Initial Codes, *Sub-codes*	Example Evidence
Anticipated benefits	Virtual visits	
	- *Interesting local sites*	"Eden project, Tintagel, Cornish mines, that kind of thing. That's the hope, and particularly post COVID with kind of reductions in mobility." (Care Home 1)
	- *Virtual tourist sites*	"Some of these tourist sites to visit. So you could go to Venice or Paris." (Workshop 1)
	Physical activity/exercise	"And it does exercise the top of the body as well. Yeah, obviously you need to push down. Yeah, take the weight off your feet when you slide. So it's, well, it is a whole body exercise really" (Care Home 5)
		"You know, it's making each day have something new or useful or different, and this could open that to them. A massive, massive but as well as you know, without them realizing bit by bit and increasing their fitness, mobility, their longevity, their life span, their health" (Workshop 2)
	- *Motivating activity*	"And you know, struggle to get [exercise], how boring it is. If they can actually walk on that something to motivate them to do it. Yes, massive motivation isn't it. " (Care Home 5)
	Prevents boredom	"Oh, it's good exercise as well as Yeah, it's actually giving them an experience. It's getting a mobile, perhaps someone that might just sit in their chair normally." (Care Home 2)
		"And [a resident] gets bored sometimes in the afternoon to the evening. And he's getting his coat or going out for a walk. Well, if we set him up on there. You could have a walk on here instead" (Care Home 5)
	Rehabilitation	"We had a bloke the other day that had a stroke. In the April he was discharged from hospital they couldn't do any more for him and he wasn't standing, wasn't walking. He went home from us because we, our staff were inputting exercise […] There was another lady […] But she was off her legs. And we've got her going and then she went home again. Yeah. If we can get close enough [to the ROVR] with the chair that they can actually sit and do it, it would be [rehabilitating]." (Care Home 5)
		"Particularly I think it'd be fantastic for rehab. Absolutely. Brilliant. People go that have to walk up and down bars." (Care Home 5)
	Staff use	"Yeah. even if our staff will be using it, our staff in front of our residents, makes good health and wellbeing staff and a morale boost. Yeah, because it's something new, something exciting that they can all talk about. So it's not just not just yeah, not just the service users, the staff as well as the whole well being." (Care Home 5)
	Social interaction	"The novelty factor and the benefits of something different to what they've experienced in it, the talking points for themselves and their families that they probably can hang on to for quite a while." (Workshop 1)
		"I'm anticipating, essentially, social interaction, with the carers a bit more because there's going to be more communication? Yeah. So enhanced communication. Yes. Exercise. Yes. Yeah. Exercise. And interesting life." (Care Home 5)
		"[They can talk to each other] Yeah. I think that's the best part of it" (Care Home 2)
		"So maybe in time, it would be nice to involve the communities in and around our houses." (Workshop 2)
	Wellbeing	"Ultimately, if they're using it, their well being is completely going to change and they're going to be happier." (Care Home 1)
		"But I think for mental health it would be good" (Care Home 2)
	Accessible experiences	"So some are just not able to get out anymore. Yeah. So if you're providing the opportunity to see the place that they love" (Care Home 4)
	- *Different to care home*	"If you're living in a care home, to go to the different, that environment was lovely." (Workshop 1)
	Stimulates the brain	"Stimulates the brain. Yeah. Got me thinking." (Care Home 1)
	Restorative	"that will bring their confidence back" (Workshop 2)
	Reminiscence	"Especially if they're walking in an area they know. They are walking down. Trigger memories" (Care Home 2)

	- *Reminiscence benefits*	"Well, I think it's memories, it's reminiscing for them, isn't it? If they went to it's like, you know, bearing in mind not everybody that lives here is from Devon or Cornwall, we've got people that have moved from the land at some point. So even to have those places it brings back memories. You know, we've got old photographs of Saltash, pictures of Saltash, and that, you know, they'll say, Oh, we went to that cinema when I was young. You know, it brings back memories. So I'm presuming this will do the same. I'll show them because yeah, A lot of living with dementia. They're living years ago." (Care Home 4)
	Expectations challenges positively	"That was alright actually, I didn't think, it wasn't as bad as I thought it was going to be. I felt more in control than I thought I would." (Care Home 2)
	Positive distraction and novelty	"What we were saying, and you know you said how was things I think, you know, people at the moment are feeling so overwhelmed. Yeah, we've been through something like this would take their minds off things" (Care Home 2)
Acceptability	Positive comments	"Yeah, that's amazing. Absolutely amazing" (Care Home 3)<br>"I honestly think this is amazing" (Care Home 6)
	ROVR is interesting	"Interesting idea." (Workshop 1)
	Adoption desired	"I would […] buy it for the staff and residents. I would consider that, I would put it before my board so that we actually had one permanently." (Care Home 5).
	Ease of use	"Easy to control" (Care Home 5)
	- *Headset initially heavy but comfortable*	"It feels a little bit heavy forward so I've got to bring it forward, but it is good." (Workshop 1)
	- *Physically easy*	"Not for me intense at all." (Workshop 1)
	- *Treadmill is easy to enter*	"In felt fine [getting in and out], because at least I had one stable foot on the ground, and sort of the bars were in front of me." (Workshop 1)
	- *Treadmill is easy to move on*	"I actually find the walking on it much easier than I thought it would be to be." (Workshop 1)
	- *VR is novel experience*	"One [benefit], the novelty factor and the benefits of something different to what they've experienced in it." (Workshop 1)
Concerns of use	Secureness	"That does feel more secure, leaning on the bars around you. And it helps to keep you stable." (Care Home 3)
	Safety concerns	"Yeah, just whilst they still think they can walk. And while that seems pretty easy, yeah, that potentially is gonna make them really unstable. Or they'll get breathless really quick" (Care Home 3)<br>"It's unsafe I would say as strong as that they would be unsafe" (Care Home 4)
	- *Hitting objects*	"... Oh, I've got a move in a minute, because I'm going to hit, say the bollard, I already hit it. And I wanted to go up the stairs. But I couldn't go upstairs because I'd hit it already..." (Care Home 1)
	- *Shoes*	"The shoes are going to be a little bit of an issue. If they can come in different sizes that might be But the smaller they are, the tighter they're going to be. So I found that quite tight to fit the one large and I've only got size three feet. Yeah. very slippery to stand on. And I can see that being quite an issue" (Care Home 1)
	- *Safety hazards*	"Maybe you need something around the outside. So if they fell, they just they can't come out. They can only go on their knees then can't they?. Do you know what I mean? If there was a guard? Actually, because that's my worry if someone fell out of that." (Care Home 5)<br>"You see that wouldn't be suitable for a resident. That is too slippery" (Care Home 4)
	- *Danger*	"That's that's a slippery slope." (Workshop 2)<br>"They [residents] can't afford to fall, if they fall it's gonna be an injury isn't it" (Care Home 6)<br>"It's a bit slippery to get on and off? That is a danger" (Care Home 5)
	Lack of control	"It's it's it's fast and then it's because you can't control it" (Care Home 3)

	- *Movement issues*	“I'm not really moving anywhere.” (Workshop 1)
	Risk of falls/Dangerous	“more support for them because the type of people that we get like I've said are not as agile as they used to be even though sometimes they think they are and they're potentially going to fall in that.” (Care Home 3)
	- *History of falls*	“I don't know that she'd be happy to do it. Because she's got a history of falls. Okay, so she’d probably not enjoy it.” (Workshop 1)
	- *Could cause falls*	“I think when it could actually make someone fall.” (Workshop 1)
	- *Risk of injury – hips*	“If you slipped now, you're gonna bang yourself on those bars, then you're gonna catch your ribs or whatever? Or your arm, break an arm on there if you go down, yeah.” (Care Home 5)<br>“Yep. Okay. I can see a few hips going.” (Care Home 2)
	Accessibility	“Getting in was alright, getting out was difficult, because you've already done that slippery surface” (Care Home 3)<br>“You could do with like a virtual reality wheelchair, they sit down and go, around.” (Care Home 5)
	Prefer people go out	“the deterioration that we've seen in so many of our residents is tenfold and I'd rather get them out. But then you have that knock on effect that the families don't want to. But if they have the option of liaising via a website, let's go and visit Eden with her” (Care Home 1)
	Heavy shoes	“Yeah, the heaviness of those shoes, and being nervous of falling.” (Workshop 1)
	Lack of motivation	“Definitely. Yeah. There's lots of ‘Mister, come on, you can do it. You can do it.’, ‘I don't want to do it’." (Workshop 1)
	Uncomfortable movement	“I feel like the twisting itself, which is where the uncomfortable sensation perhaps maybe lies.” (Workshop 1)
	Bigger environment more physically demanding	“Depends on how big the world is, because this world feels small and so I feel like I have to twist.” (Workshop 1)
	Critique	“I wouldn't want them using it without a staff member on the site.” (Workshop 2)<br>“Resident would be screaming the place down, and panic about the step [into ROVR]” (Care Home 6)
	Potential issues	“this idea of being in an open space or field might be disorientating” (Workshop 2)
Concerns of negative effects	Side effects	“Yeah. It wasn't motion sickness but I just started to feel like I just need to take this off now, yeah, it's just a really strange feeling” (Care Home 3)<br>“Yeah, I've got a feeling of like, yeah, maybe I've got like a bit of car sickness.” (Care Home 4)
	- *Encouraging shuffling/sliding*	“That is almost the encouraging of Parkinson's type shuffling isn't it?” (Workshop 1)
	- *Unnatural movement*	“As you move around this change of texture to this and that makes it seem like it's not as organic or not as natural” (Workshop 1)<br>“A bit weird, because you’re not stepping” (Care Home 5)<br>“Keep sliding […] it’ll feel a bit weird” (Care Home 6)
	Dizziness	“I might feel dizzy. It was alright there but when you go off the steps and stuff you start getting a bit. It's because when you went the faster you go. Then when you turn that's when you get a bit. Isn't it like discombobulated?” (Care Home 5)<br>“It feels really weird, my head is still spinning” (Care Home 6)
	Disorientating	“That’s amazing but it messes with your head” (Care Home 3)
	- *Orientating to environment*	“Trying to orientate yourself is quite difficult. Yeah. Yeah. Especially around the house.” (Care Home 1)<br>“Especially when you got the headset on, you're disorientated as well.” (Care Home 4)<br>“The only thing that concerns me is the disorientation that. Yeah, that's the only thing. Okay, Whether they'd be disorientated.” (Care Home 2)<br>“Yeah, I think it'd be really hard because you lose all your visual cues like even though I've got no balance problems it was pretty tricky. You do certainly do lose your orientation before you put those on, like, I didn't know where anyone was. Deal with the disorientation and the

		nausea. Anyway, which they might not be able to tell you about, so I'd be worried about it for dementia. Without a lot of, it's worth trying." (Workshop 1)
	- *Coordination between VR and feet*	"Yeah, since you've had the headset on your feet don't seem in momentum. Is it difficult to watch and organise your feet?" (Care Home 4)
	- *Height changes*	"It [*disorientation*] didn't affect me till there was like the change in height" (Workshop 1)
	- *Motion*	"Motion is kind of backwards and forwards, like you're on a cross trainer." (Workshop 1)
	- *Social angle confusing*	"For a few? Most of them I would say it [social experience in VR] would be very confusing." (Workshop 1)
	Concentrating more on the exercise	"Concentrating more on the exercise." (Workshop 1)
	Depth perception difficulties	"Because it is such a such a strange sensation of a lot of different coordination at the same time, and that the depth perception issues" (Workshop 1)
	Motion sickness in VR	"Oh, I'm actually feeling really dizzy." (Workshop 1)<br>"I felt motion sickness when I got off yeah. But I think it was too enclosed. Like, there's too many walls and things. I hit a wall quite quickly," (Care Home 4)
	Fear	"I think some elderly people would find it quite scary. Yeah, I do." (Care Home 1)<br>"They [residents] might feel a bit scared, because because it's slippy." (Care Home 2)
Suitability/Unsuitability	Extra support	"I think the worry for me is is if we get to the stage where the residents go in there. Yeah, I think we need to have much more support for them" (Care Home 3)
	- *Supervised use required*	"I don't think I'd feel happy start with if they weren't being supervised." (Care Home 2)<br>"Yeah, but you still need to hold on it. But I think what you would always do with the residents on that, or you would always have one person here" (Care Home 5)
	- *Guidance and guided navigation*	"I was just saying, if your staff can't see what's going on, when you run into problems, they can't troubleshoot it for someone who's struggling cognitively, or even just unfamiliar with it, like, am I on the balcony? Do I need to get to it? If I'm not on the balcony, how do you guide someone up to it? What do I do? They can't then put the mask on, with infection, you can just put the mask on and check, you've got to take someone's headset off." (Workshop 1)
	- *Human resources*	"I do think it will need some supervision, so definitely, we're going to have to get the staff on board with it. Because well, I, it's not something that I would expect to walk in at nine o'clock in the morning and see someone on it without one of us being there" (Workshop 2)
	- Number of residents	"they all they've all got walking frames and need wheelchairs. And even that's residential. I would say only a couple." (Care Home 1)<br>"I can think of one called [resident name]. Yeah, that's about the only one I will allow would feel comfortable with." (Care Home 4)<br>"Obviously you just have to pick the residents that would be appropriate for, because someone with mental health issues would really struggle on this." (Care Home 4)
	- *Differences between residents*	"[Suitable for residents with] More stability when they're on their feet" (Care Home 5)
	- *Residents unable to use*	"I don't know if a resident would be able to do that" (Care Home 1)
	Diagnosis	"Imagine that somebody with cognitive impairment or a spatial awareness issue, poor eyesight, mobility wise, […] not somebody of age (Care Home 6)

	- *Balance*	"I think it's the overall, you know, they are on lots of medication, how that's going to affect their balance. I've got some people who have vertigo, they wouldn't be able to do it." (Care Home 1)
	- *Dementia/cognition*	"First, immediately, people are just gonna let go. Okay, especially if they got mild dementia and something like that" (Care Home 1)<br>"And of course they are in a not in a reality situation. So they don't know actually where they are." (Care Home 5)<br>"Has anyone said about the issue about with the mental health. They might think why are here? Yeah. Why are we keeping them from them. There's some here that would love to be in Australia with the granddaughter and they would fully understand that it's a Virtual world and also coming back out and leaving that experience then suddenly the grandchild is gone again. Someone living with dementia only got to see somebody crossing the car park, and they think it's one of their family and they think why do you not let them in, it's not their family but they think." (Care Home 4)<br>"I feel like it would totally freak our clients [...] I think better cognition" (Care Home 6)
	- *Fear of falling*	*Interviewer:* "Yeah. Do you think that that slipperiness would be a problem? "<br>*Participant:* "It would be for some of our residents here. Yeah. Yeah. They would just fall over" (Care Home 1)<br>"Just what we've already said, totally inappropriate for residents because if they fall." (Care Home 4)
	- *Have to be able*	"They've got to be quite able. So our most able person is is she's done the, the other one with us. She goes out gardening, she goes out doing lots of things. But she's had a fall, because she was pulling weeds out and one was too strong. So it can easily be done. That then will knock her confidence. So I don't want her going on something like this that she wasn't that keen. Yeah. Okay. So they have to be very able" (Care Home 1)<br>"In fact, they because I've had to hold here. They would have to hold on really tight. Yeah. And because they're older this the grip alone." (Care Home 4)
	- *Incontinence*	"So you might have to look around that a little bit. Yeah. Especially with people that are already afraid of falling. Yeah, they're incontinent because they fall. They don't want you know, they don't want to go to the toilet, because so going on something like this might be a little bit of an issue." (Care Home 1)
	- *Influence of medication*	"I think it's the overall, you know, they are on lots of medication, how that's going to affect their balance. I've got some people who have vertigo, they wouldn't be able to do it" (Care Home 1)
	- *Limited mobility*	"This bit as well. I mean, we've got here they're not very mobile or anything." (Care Home 1)<br>"We're got a few that are quite mobile. Even the ones that could walk I think would really struggle." (Care Home 5)
	- *Problems with vision*	"A little bit. Getting better but it's still quite blurry though." (Care Home 4)
	- *Neck pain*	"I was like that well, going around rather than like that, that would that give you the neck neck ache and then that, okay, posture? Yeah" (Care Home 1)
	- *Neuromuscular*	*Interviewer*: "So thinking about maybe some of your family members, then what kind of conditions or challenges do you think a person would have that would mean this wasn't suitable?"<br>*Participant:* "Neuromuscular degeneration is one for me." (Workshop 1)
	- *Lived problem*	"would that be important that actually there was an ongoing service? Rather than you buy this and you're left on your own? Would you prefer it that actually, perhaps I pay a monthly fee? And I know, I can always pick up the phone if something goes wrong and contact that company? Would you rather kind of buy it and own it? Or have that kind of that long term rental maintenance and the service that comes with it?" (Workshop 2)

	- *Vertigo*	“For someone with that impairment, or someone with that illness, that's just not suitable for them. What about someone with vertigo?” (Care Home 2)
	Suitability for residents	“What do you think with the residents? I just I just said there's not many that we'll be able to put on that.” (Care Home 5)
	- *99% not suitable*	“99% won't be able to do it anyhow” (Workshop 1)
	- *Suitable for residents 20 years younger*	“Twenty years ago when we had residents, like you can think we still got residents that would have been fine but now. No way” (Workshop 1)
	- *Wouldn’t recommend to people with mobility issues*	“Unsteady gait in general, having vertigo, history of, history of falls in any sense, increasing that risk of. I wouldn't bother I think.” (Workshop 1)
	Ethics	“There's still challenges, though, isn't there of getting on and off like exercise, you know, I've taken loads of people on it, like that, it can be quite a dodgy thing to do. It's not okay, so yeah, there's still challenges with getting older adults on and off these things, especially anyone who has cognitive impairment,” (Workshop 1)
	Risk	“Older adults wouldn't be able to even step over that even though she's on it […] And probably in nursing homes older people's standing tolerance may not be that. And the effect on balance from turning, as well as the nausea point of view” (Workshop 1)
	Size of technology	“It is massive. I didn't expect it to be this big.” (Care Home 1)
	- *Wifi connectivity*	“It would probably be in the lounge because probably we get quite good reception in the lounge. Sometimes in parts of the building we might lose it at times.” (Care Home 4)
	Familiarisation	“And just like anything else, it's time to get used to it. That familiarization with. But that's like everything that we have that's new. Once people are familiar with it, and it's normal. I mean, to our residents, this sort of technology is quite strange, anyway,” (Care Home 2)
	- *Adapting to use*	“Because I'm, I've got a bad back. But I was able to still able to do it. I got poor eyes because I've got wet macular degenerate disease but it was fine. Okay, I couldn't do as long as what [other participants] did probably, but also it's my first time in VR so I was cautious but as you get used to it.” (Care Home 5)
	- *Unfamiliar*	“I can just see a pillar here. I could hear someone it's like the pillars talking […] It's like I'm disorientated” (Care Home 5)
	- *Adjusting to headset*	“And when the VR went on, very disorientating, very blurry, and for then to adjust it as they want it, I think might be an issue. So you need to be able to see what they're seeing on the computer to you maybe have to adjust it that” (Care Home 1)
	- *Familiarity*	“I certainly think the fact that you're going to have destinations, local destinations will be the attraction.” (Workshop 2)
Improvements	Content suggestions	“Under the sea would be good wouldn't it?” (Care Home 5)<br>“We could take them to places. If we could get in the gardens every year. If you could capture that.” (Care Home 4)<br>“Because you really you're taking them to places you know like the Beaches.”(Care Home 4)
	- *Cartoon-like images*	“You want to touch the flowers, so they will let go and then they'll Yeah, yeah. So I do think that's a bit of a challenge mobility, and that's quite cartoonish as well. So will it be reality?” (Care Home 1)
	- *New environments*	“Yeah, Mount Edgecumbe. All those kinds of things that they they would love. Even the Tamar Bridge. A garden center. You know, last gardens of Helligen, Eden Project. I think they'd love it. Yeah, I think they'd really love it. And they're sitting down and they can look at the flowers. And, you know, pick, they can do all these kinds of things. But if they're standing up, I think there'll be more "I don't like it. I don't like it", because it's do you see what I mean? Yes. Yeah,” (Care Home 1)
	Inclusivity	“how we could adapt the equipment for her typically she has full capacity and would really benefit from this but obviously it's a double amputee so she's not going to physically walk” (Care Home 3)
	- *Adaptable bar height*	“Yeah, even if that was that was, you could undo it and take that bit off. You only need an eye and a button don't you like you don't like the walking sticks with the eyes and the button you only need a click button and then you could make it different sizes as well. It could go back further, be adaptable. They have something to hold on to in front of them.” (Care Home 5)

	- *Fitting to a wheelchair*	"Even if it's just like a normal chair. Yeah. Not a wheelchair. Because when you soon as you get in a wheelchair, you think you're going somewhere, which might be a good thing." (Care Home 1)
	- *People less mobile*	"You know, most of them come in in wheelchairs. Okay. That's the way residents are coming in." (Workshop 1)
	- *Access could be improved*	"You have to naturally be able to step up, don't you? So if you could step up onto like, just a something there, as a gap in between." (Workshop 1)
	- *Physical requirements*	"From a physical perspective. I have to say this is a dance of what they have been used to before, but I would think that whoever would need to, to safely do this would already have a degree of practicality about them." (Workshop 1)
	Safety improvements	"There's that feeling of going back. So I think if there were two front legs and at the back, that would give much more support?" (Care Home 3)<br>"I think you're looking at someone with a degenerative physical disease, but good cognition, once you've made some modification, so it doesn't kill them." (Workshop 1)
	- *Softer impact bars*	"If they do, if a resident slips out, as in legs come down. Obviously, we would need some sort of buffers over the top of the bars," (Care Home 5)
	- *Stop slipping*	"I don't know, do you need to have it slippery actually." (Care Home 5)
	- *Awfully slippery*	"Awfully slippery. So you're, you're like when you're walking on ice or wherever you're on guard. So that takes away the enjoyment." (Workshop 1)
	- *Difficulty exiting treadmill*	"I'm not sure how many people would be able to get out of that." (Workshop 1)
	Add arm movements	"You have like any interaction with hand stuff as well. You know you've got your feet Yeah, you do. So you have things in your hands so" (Care Home 5)
	Cast visual for group to watch	"Yeah. I don't know if we could connect to this screen, so the rest of them can actually see on the screen where this [in the VR] is." (Care Home 5)
	Seated version	"The seated unit would be more beneficial to our client base, as we are at the moment." (Care Home 2)<br>"It would have to be a sitting one, but would they like that [VR headset] on their head" (Care Home 6)
	- *Square*	"If it was sitting down. Yeah. Yeah, that would be different." (Workshop 1)
	- *Headset use only*	"I would think if it was less slippery, yeah. And maybe if you could, well, you couldn't just use the headset. Could you? Could you just use the headset?" (Care Home 1)
	- *Adaptations*	"Could they not sit? If they were sat using their feet Yeah. But then they wouldn't get do you have to. Is there a was a chair scenario." (Care Home 4)
	Possibilities/exercise bike	"I wonder about it on a bike because that way. You've got your bike, which turns, so pedalling is innate. People know peddling goes forwards. And so on. And if you could turn on that. I wonder if it would keep you a little bit more orientated? Because all your movement has been done with that and with the steering wheel aspect to that. And so I don't know if that would be better. If you're trying to get the exercise. Cause it's really interesting. You're looking at it from the exercise point of view. If it's just getting someone out and about." (Care Home 5)
Current design	Good content	"I'm just getting to the balcony. Oh my god. Oh that is amazing brilliant. It is amazing that is phenomenal." (Care Home 5)<br>"But it just feels so real" (Care Home 4)<br>"It's beautiful" (Care Home 6)
	- *Visual features*<br>- *Spatial features*	"No, I don't want to go down the steps. I'm gonna go this way. I don't want to go down the steps, I don't like it" (Care Home 4)
	Liked headset	"It doesn't feel heavy on my head" (Care Home 5)

Bradwell HL, *et al. BMJ Open* 2023; 13:e073307. doi: 10.1136/bmjopen-2023-073307

		"Yeah, that's good, it feels really comfortable" (Care Home 4)
	Safety features	"That's obviously why the bars are there" (Care Home 5)
	Technology issues	"However much walking I got I wasn't going anywhere." (Care Home 5)
	- *Glitching*	"You're actually flying all over the place […] you are there but you're flashing all over it as well." (Care Home 5)
	- *Loading worlds*	"All I got is clouds and a green line. It's just a green line." (Care Home 5)
	- *Speed of movement*	"I'm guessing I would say that one slide of that foot? You'd probably moving about two feet." (Care Home 5)
	Ergonomics	"I'm stepping in a way I wouldn't normally step" (Care Home 5)<br>"They didn't feel big on the feet. Because you're sliding" (Care Home 4)
	Mechanics	"It doesn't feel like natural walking." (Workshop 2)
	Resolution	"it's going to feel a lot more stable with that in there, and so they've suggested that when people have their first experiences, it's always with the training mats in place." (Workshop 2)
