# Supplementary material for: Staff perceptions towards virtual reality-motivated treadmill exercise for care home residents: a qualitative feedback study with key stakeholders and follow-up interview with technology developer
Source: BMJ Open. 2023 Nov 23;13(11):e073307. doi: 10.1136/bmjopen-2023-073307 (PMC10668234; doi:10.1136/bmjopen-2023-073307)
Supplement: Author's manuscript [file bmjopen-2023-073307.draft_revisions.pdf]

BMJ Open is committed to open peer review. As part of this commitment we make the peer review history of every article we publish publicly available.

When an article is published we post the peer reviewers' comments and the authors' responses online. We also post the versions of the paper that were used during peer review. These are the versions that the peer review comments apply to.

The versions of the paper that follow are the versions that were submitted during the peer review process. They are not the versions of record or the final published versions. They should not be cited or distributed as the published version of this manuscript.

BMJ Open is an open access journal and the full, final, typeset and author-corrected version of record of the manuscript is available on our site with no access controls, subscription charges or pay-per-view fees (http://bmjopen.bmj.com).

If you have any questions on BMJ Open's open peer review process please email info.bmjopen@bmj.com

Perceptions of staff towards virtual reality motivated omnidirectional treadmill exercise for older adult care home residents: A qualitative feedback study.

Journal:	*BMJ Open*
Manuscript ID	bmjopen-2023-073307
Article Type:	Original research
Date Submitted by the Author:	01-Mar-2023

Complete List of Authors:	Bradwell, Hannah; University of Plymouth<br>Cooper, Leonie; University of Plymouth<br>Edwards, Katie; University of Plymouth, Faculty of Health<br>Baxter, Rory; University of Plymouth<br>Tomaz, Simone; University of Stirling<br>Ritchie, John; University of Stirling<br>Gaudl, Swen; University of Plymouth<br>Veliz-Reyes, Alejandro; University of Plymouth<br>Ryde, Gemma; University of Stirling<br>Križaj, Tanja; University of Plymouth<br>Warren, Alison; University of Plymouth<br>Chatterjee, Arunangsu; University of Plymouth, Centre of Health Technology; University of Leeds, School of Medicine<br>Willis, Katharine; University of Plymouth<br>Haynes, Richard; University of Stirling<br>Hennessy, Catherine; University of Stirling<br>Whittaker, Anna; University of Stirling<br>Asthana, Sheena; University of Plymouth<br>Jones, Ray; University of Plymouth

Keywords:	Virtual Reality, Physical Therapy Modalities, Information technology < BIOTECHNOLOGY & BIOINFORMATICS

SCHOLARONE™
Manuscripts

I, the Submitting Author has the right to grant and does grant on behalf of all authors of the Work (as defined in the below author licence), an exclusive licence and/or a non-exclusive licence for contributions from authors who are: i) UK Crown employees; ii) where BMJ has agreed a CC-BY licence shall apply, and/or iii) in accordance with the terms applicable for US Federal Government officers or employees acting as part of their official duties; on a worldwide, perpetual, irrevocable, royalty-free basis to BMJ Publishing Group Ltd ("BMJ") its licensees and where the relevant Journal is co-owned by BMJ to the co-owners of the Journal, to publish the Work in this journal and any other BMJ products and to exploit all rights, as set out in our licence.

The Submitting Author accepts and understands that any supply made under these terms is made by BMJ to the Submitting Author unless you are acting as an employee on behalf of your employer or a postgraduate student of an affiliated institution which is paying any applicable article publishing charge ("APC") for Open Access articles. Where the Submitting Author wishes to make the Work available on an Open Access basis (and intends to pay the relevant APC), the terms of reuse of such Open Access shall be governed by a Creative Commons licence – details of these licences and which Creative Commons licence will apply to this Work are set out in our licence referred to above.

Other than as permitted in any relevant BMJ Author's Self Archiving Policies, I confirm this Work has not been accepted for publication elsewhere, is not being considered for publication elsewhere and does not duplicate material already published. I confirm all authors consent to publication of this Work and authorise the granting of this licence.

For peer review only

Perceptions of staff towards virtual reality motivated omnidirectional treadmill exercise for older adult care home residents: A qualitative feedback study.

Bradwell, H.L[1*]. Cooper, L[1]., Edwards, K.J[1]., Baxter, R[1]., Tomaz, S.A[2]., Ritchie, J[2]., Gaudl, S[1]., Veliz-Reyes, A[1]., Ryde, G.C[2]., Krizaj, T[1]., Warren, A[1]., Chatterjee, A[1 3]., Willis, K[1]., Haynes, R[2]., Hennessy, C.H[2]., Whittaker, A.C[2]., Asthana, S[1]., Jones, R.B[1] on behalf of the GOALD project.

[1] Centre for Health Technology, University of Plymouth, Plymouth, UK.

[2] University of Stirling, Stirling, UK.

[3] School of Medicine, University of Leeds

*Corresponding author: Hannah.bradwell@plymouth.ac.uk

For peer review only

Abstract

Objectives
Health and care resources are under increasing pressure, partly due to the ageing population. Physical activity supports healthy ageing, but motivating exercise is challenging. We aimed to explore staff perceptions towards a virtual reality (VR) omnidirectional treadmill (ROVR), aimed at increasing physical activity for older adult care home residents.

Design
Interactive workshops and qualitative evaluation.

Settings
Eight interactive workshops were held at six care homes and two university sites across Cornwall, England from September-November 2021.

Participants
Forty-four staff participated, including care home, supported living, clinical care and compliance managers, carers, activity coordinators, occupational therapists, and physiotherapists.

Methods
Participants tried the VR treadmill system, followed by focus groups exploring device design, potential usefulness, or barriers for care home residents. Focus groups were audio recorded, transcribed verbatim and thematically analysed. We subsequently conducted a follow-up interview with the technology developer (September 2022), to explore feedback impact.

Results
Analysis produced seven key themes: anticipated benefits, acceptability, concerns of use, concerns of negative effects, suitability/unsuitability, improvements, and current design. Participants were generally positive towards VR to motivate care home residents' physical activity and noted several potential benefits (increased exercise, stimulation, social interaction, and rehabilitation). Despite reported potential, staff had safety concerns for frail older residents, due to the standing position. Participants suggested design improvements to enhance safety, usability, and accessibility. Feedback to the designers

For peer review only - http://bmjopen.bmj.com/site/about/guidelines.xhtml

resulted in development of a new seated VR treadmill to address concerns on falls while maintaining motivation to exercise. The follow-up developer interview identified significant value in academia-industry collaboration.

Conclusion

The use of VR motivated exercise holds potential to increase exercise, encourage reminiscence, and promote meaningful activity for care home residents. Staff concerns resulted in a re-designed seated treadmill for those too frail to use the standing version. This novel study demonstrates importance of stakeholder feedback in product design.

Key words: social care, physical activity, technology, virtual reality, innovation, knowledge exchange

Word count: 4342

Article Summary

Strengths and limitations of this study

- Relatively large sample of participants for qualitative research providing breadth of perceptions and data saturation
- Rigorous qualitative analysis on novel topic by a research team with diverse (yet, related to the topic) disciplinary backgrounds (including engineering, health, design)
- Explored perceptions of key stakeholders (health and social care staff)
- However, limited interaction durations between participants and the technology
- No perceptions gathered from older adult stakeholders

Background

Introduction

Pressure on health and social care (H&SC) resources is increasing, because of increased life expectancy and decreasing numbers of care workers [1, 2]. Therefore, there has been a push for research and innovation into technologies to support healthy ageing [3, 4]. Reducing loneliness and remaining active are key strands of improved wellbeing for older adults [5, 6]. Physical activity has numerous health benefits for older adults, helping reduce risks of diseases such as cancer, diabetes, cardiovascular disease and stroke, and delaying dementia onset [6]. Being consistently active for over six months has also been associated with reduced incidence of falls [7]. However older adults do little exercise and have difficulties maintaining exercise over time [6].

One technology of interest in supporting older people with meaningful activity, motivating exercise, and encouraging social connectivity is virtual reality (VR). There have been increasing numbers of studies exploring VR use within care settings [8]. Here we report a study of a VR and omnidirectional treadmill system, ROVR Adventure (ROVR), designed for older adults [9]. The system is designed to motivate activity, physically navigating virtual

For peer review only - http://bmjopen.bmj.com/site/about/guidelines.xhtml

For peer review only

environments that have meaning for the users. The use of environments with meaning to the user is essential to achieve the aim of meaningful activity, which, by definition, must be tailored to the users' preferences [10].

The VR environments in ROVR include places of cultural and historical interest such as ancient forts, museums, nature scenes and cityscapes. These 3D virtual environments represent places care home residents may no longer be able to visit physically. Access to culture (including heritage and history) is a human right [11], with documented health and wellbeing benefits, including improved quality of life, opportunity for reminiscence and connectedness to spaces and history [12]. Older adults in particular are invested in heritage, yet physical access to sites of cultural or heritage interest is often limited due to site characteristics and conservation issues (e.g., uneven ground, inability to implement accessibility features) [13]. The Heritage Alliance annual report [12] recommended partnership between heritage and health, to overcome access challenges to help realise the full potential of heritage for health and wellbeing. The Covid-19 pandemic highlighted the potential for online access to culture and heritage in place of physical access [14], with VR offering possible solutions [15]. The use of VR to visit cultural and heritage sites can therefore aid in inclusivity for those with physical and sensory barriers [15], particularly prevalent among older adults. The opportunity for reminiscence brings additional wellbeing potential, with reminiscence thought to provide stimulation, enjoyment, sense of self-worth and achievement [16].

Alongside potentially motivating physical activity and providing access to cultural and heritage sites, ROVR allows for 'walks' to be socially interactive, with multiple users joining the same virtual environment to 'walk and talk' together, regardless of their location. The aim of the system therefore is to enhance social connectedness and improve physical health through meaningful activity [17]. However, prior to this study ROVR had never been used in care homes. With any technology, there may be significant differences in opinions between developers and end-users [18] so older people's feedback in design of technology is essential. Given potential safety concerns and the need to understand how to implement the technology we first needed to explore care home staff opinions on feasibility, design, and potential for older care home residents.

Method

Aims

The aims of this study were to explore staff perceptions towards acceptability and feasibility of the ROVR Adventure system for older adult care home residents. Results from the study were provided to the technology developer to help design iterations.

Design

This study was a qualitative feedback study, allowing staff to try, then to discuss, the equipment. Findings were presented to the technology developer allowing them to modify the design. We interviewed the Chief Executive about this and their experience of business

For peer review only - http://bmjopen.bmj.com/site/about/guidelines.xhtml

working with research. This manuscript has been constructed following the COREQ checklist for qualitative research [19].

Location and dates

Data collection on perceptions of the ROVR standing treadmill and VR system took place from September to November 2021 in Looe, Liskeard, Saltash, St Austell, Perranporth, Camborne, and Truro in Cornwall UK. A follow-up interview with the technology developer took place via Zoom in September 2022.

Materials

Figure 1 shows the ROVR Adventure omnidirectional treadmill, with a Pico Neo 2 VR headset to immerse the users in the virtual environments. The headset is equipped with a 4K LCD display that provides a resolution of 1920 x 2160 pixels per eye with a field view of 101 degrees. The treadmill is a smooth plastic dish coated thinly with lubricant on which people stand wearing overshoes with ceramic plates on the base. This allows for a low friction, slippery surface on which people 'slide' their feet backwards and forwards to represent walking. The treadmill links to the ROVR PC software (in this study we ran the programme on a Lenovo ThinkPad L13) and VR system (Figure 1) via Bluetooth, which together, translates movement on the treadmill into movement through VR worlds. To change direction, participants swivel around and point their bodies in the direction of desired travel. VR worlds include 3D scanned, real world, heritage locations as well as animated worlds ranging from museums to simple houses (Figure 2). The ROVR VR worlds can also be social experiences, with several users entering the same world from distant locations and walking and talking together.

(Figure 1 and 2 here)

Procedure

Convenience sampling was used. The health professionals were recruited at University of Plymouth e-Health events, through approach by the researcher (HB). Health professionals read participant information sheets and signed written consent forms before engaging in the workshop. The care homes were approached by email by HB, chosen based on prior collaboration with the researcher, to scope interest in trying the ROVR. For care home contacts who were interested, researcher visits were arranged, and participant information sheets and consent forms were provided. All participants provided written informed consent prior to taking part. Two researchers (HB, KE, both PhD holders with a background in digital health research and specific interest on social care) engaged in eight interaction workshops where a total of 44 key stakeholders (health and care staff) were provided with demonstrations of the ROVR Adventure and the opportunity to try the technology themselves. All staff tried the ROVR having at least 5 minutes of interaction. We used audio recording devices to capture comments during use. All 44 staff then participated in focus group discussions to share their perceptions. These were also audio recorded. Sessions lasted from 43-110 (mean 71) minutes. Sessions had between three and eight participants (Table 1). We were unable to link participants to distant ROVR users as a social experience

For peer review only - http://bmjopen.bmj.com/site/about/guidelines.xhtml

due to feasibility but did demonstrate two users in one world (the researcher on laptop and participant on treadmill), to facilitate a discussion around the potential social experience. One of the workshops engaged health professionals such as occupational therapists and physiotherapists, and the other seven workshops engaged care home staff and managers.

For the one follow-up interview, the technology developer was invited to take part in an informal interview to discuss their perceptions of the impact of the academic feedback from the focus groups on their products and processes. The interview was conducted by a separate researcher (AW) to those who had been collaborating with the technology developer for the purpose of this study, to avoid bias.

Ethics

This study received ethical approval from the Faculty of Health Ethics Committee at the University of Plymouth, project ID 2887 (August 2021). All participants gave written informed consent.

Patient and public involvement

Considering the timing of this study, with health and care staff capacity still limited due to ongoing effects of the Covid-19 pandemic, it was not possible to involve them in the design, reporting or dissemination of this research.

Data Collection

Stakeholders provided feedback during their interaction with the ROVR Adventure treadmill and following the interaction during informal focus groups (interview schedule available in Appendix A). The feedback was audio-recorded and transcribed verbatim. The interview with the technology developer was also audio recorded and transcribed verbatim, there was no interview schedule used, but general queries posed to the developer on their perceptions towards the feedback from this study.

Data Analysis

Researchers (HB, LC, RB, ST, JR, AW, TK, SG, ARV) analysed the transcripts using thematic analysis. Braun et al. [20] detail the process of thematic analysis, a form of analysing qualitative data that is often used in health and social sciences. The process involves becoming familiar with the data set, generating initial codes, searching for themes, and reviewing and defining those themes. The sub-type of thematic analysis used was reflexive, in generating codes from explicit content, which evolves and adapts through considerable analytic work, to produce themes, representing an understanding of meaning across a dataset [20]. Analysis was inductive, as researchers had pre-determined aims. Analysis was conducted by nine researchers due to the size of the dataset, with a considerable amount of qualitative data collected. HB, LC and RB led on iterations of reviewing and defining themes, however, the whole research team had two meetings to explore different perspectives on the data and codes to enhance validity of interpretation through perspectives from multi-disciplinary expertise (occupational therapy, psychology, physical activity science, computer

For peer review only - http://bmjopen.bmj.com/site/about/guidelines.xhtml

science, social care research, digital design). We used the NVivo 12 data analysis software for data management to ensure a clear audit trail, enhancing dependability [21]. The involvement of a multidisciplinary team with various expertise in the data analysis processes enhances the credibility of our findings [22], the researchers who conducted analysis had diverse (yet, related to the topic) disciplinary backgrounds (including engineering, health, design), increasing robustness and significance of results (all researchers included have or are working towards a PhD). The process for the analysis of the follow-up interview with the technology developer was the same, although conducted by GR, AW and HB.

Participants

In total, across the eight interaction workshops, 44 participants interacted with the ROVR systems VR treadmill and provided evaluative feedback (Table 1). For the follow-up interview with the technology developer there was one participant, the chief operating officer of the ROVR Systems technology developer.

Table 1: Participants at each interaction workshop

Site	Participants (n)
Workshop at University site 1	(5) General health and care professionals including occupational therapist, physiotherapist and patient involvement expert
Workshop at University site 2	(3) Supported living manager and care home staff
Care home 1 – 26 bed residential care home	(3) Care home manager, compliance manager, activity coordinator and carer
Care home 2 – 83 bed nursing home with unit for people with dementia	(7) Care home compliance manager, clinical needs manager, activity coordinator and carers
Care home 3 – 47 bed care home for people with dementia	(6) Care home manager and carers
Care home 4 – 33 bed care home	(8) Care home manager and carers
Care home 5 – 40 bed care home	(7) Care home manager and carers
Care home 6 – 60 bed nursing home for people with dementia	(5) Residential care nursing manager, carers

Results

Section 1: Workshop results

Here we discuss the results of the eight interactive workshops exploring stakeholder perceptions towards the VR motivated ROVR treadmill. In section 1, quotes have been identified by site where the data were collected rather than by individual participant. The roadshow/workshop nature of data collection and recording meant identifying individuals within busy transcripts was not feasible. In section 2, we share the results of the follow-up interview with the technology developer. Table 2 shows the themes and codes identified in the analysis of the eight workshops.

For peer review only - http://bmjopen.bmj.com/site/about/guidelines.xhtml

Table 2: The results of thematic analysis of the 8 interaction workshops. Evidence to support each code can be found in Appendix B and is synthesised in narrative below.

**Theme**	**Initial Codes, *Sub-codes***
Anticipated benefits	Virtual visits; *Interesting local sites, Virtual tourist sites*<br>Physical activity/exercise; *Motivating exercise*<br>Prevents boredom<br>Rehabilitation<br>Staff use<br>Social interaction<br>Wellbeing<br>Accessible experiences; *Different to care home*<br>Stimulates the brain<br>Restorative<br>Reminiscence; *Reminiscence benefits*<br>Expectations challenges positively<br>Positive distraction and novelty
Acceptability	Positive comments<br>ROVR is interesting<br>Adoption desired<br>Ease of use; *Headset initially heavy but comfortable, Physically easy, Treadmill is easy to enter, Treadmill is easy to move on, VR is novel experience*
Concerns of use	Secureness<br>Safety concerns; *Hitting objects, Shoes, Safety hazards, Danger*<br>Lack of control; *Movement issues*<br>Risk of falls/Dangerous; *History of falls, Could cause falls, Risk of injury - hips*<br>Accessibility<br>Prefer people go out<br>Heavy shoes<br>Lack of motivation<br>Uncomfortable movement<br>Bigger environment more physically demanding<br>Critique<br>Potential issues
Concerns of negative effects	Side effects; *Encouraging shuffling/sliding, Unnatural movement*<br>Dizziness<br>Disorientating; *Orienting to environment, Coordination between VR and feet, Height changes, Motion, Social angle confusing*<br>Concentrating more on the exercise<br>Depth perception difficulties<br>Motion sickness in VR<br>Fear
Suitability/Unsuitabilit y	Extra support; *Supervised use required, Guidance and guided navigation, Human resources, Number of residents, Differences between residents, Residents unable to use*

For peer review only - http://bmjopen.bmj.com/site/about/guidelines.xhtml

	Diagnosis; *Balance, Dementia/cognition, Fear of falling, Have to be able, Incontinence, Influence of medication, Limited mobility, Problems with vision, Neck pain, Neuromuscular, Lived problem, Vertigo*<br>Suitability for residents; *99% not suitable, Suitable for residents 20 years younger, Wouldn't recommend to people with mobility issues*<br>Ethics<br>Risk<br>Size of technology; *WiFi connectivity*<br>Familiarisation; *Adapting to use, Unfamiliar, Adjusting to headset, Familiarity*
Improvements	Content suggestions; *Cartoon-like images, New environments*<br>Inclusivity; *Adaptable bar height, Fitting to a wheelchair, People less mobile, Access could be improved, Physical requirements*<br>Safety improvements; *Softer impact bars, Stop slipping, Awfully slippery, Difficulty exiting treadmill*<br>Add arm movements<br>Cast visual for group to watch<br>Seated version; *Square, Headset use only, Adaptations*<br>Possibilities/exercise bike
Current design	Good content; *Visual Features, Spatial Features*<br>Liked headset<br>Safety features<br>Technology issues; *Glitching, Loading worlds, Speed of movement*<br>Ergonomics<br>Mechanics<br>Resolution

Anticipated Benefits

Participants noted a range of perceived benefits of using ROVR, with many focusing on the potential for physical benefits, such as providing "*good exercise*" (Care Home 2) and being "*particularly […] fantastic for rehab*" (Care Home 5). Participants noted that finding motivation to complete standard exercises can be a "*struggle*" and "*boring*" (Care Home 5), while the VR content would mean "*they can actually walk on that, something to motivate them to do it*" (Care home 5).

Participants also noted the potential enhanced accessibility of "*tourist sites*" (Care Home 5), enhancing visiting potential, particularly for those with "*reductions in mobility*" (Care Home 1). One staff member reported "*some [residents] are just not able to get out anymore […] you're providing the opportunity to see the place that they love*" (Care Home 4). This experience was additionally expected to create "*enhanced communication*" (Care Home 5) prompting conversations between residents with each other and the staff. Care staff additionally felt that reminiscence could be an additional benefit as it may "*trigger memories*" for the residents. (Care Home 2).

For peer review only - http://bmjopen.bmj.com/site/about/guidelines.xhtml

For peer review only

Acceptability

Further to the range of potential benefits noted by care staff, participants generally demonstrated good acceptability towards the device, often praising the technology as "*amazing, absolutely amazing*" (Care Home 3), "*honestly I think it's amazing*" (Care Home 6). One care home manager even reported that she would "*buy it for the staff and residents […] so that we actually had one permanently*" (Care Home 5).

Concerns of Use

Despite the general acceptability and praise towards the concept, and appreciation for potential benefits, participants noted some concerns about suitability of such devices for older adults and the care setting. Most concerns related to the safety of vulnerable older adults using a low-friction, stand-up treadmill. One care home manager reported that for care home residents specifically, the design was "*unsafe*" (Care Home 4), with another suggesting it was "*a danger*" due to being "*slippery*" (Care Home 5). The worry was that generally care home residents are "*not as agile as they used to be*" and could "*potentially fall*" (Care Home 3). This could result in injuries to "*ribs […] or your arm, break an arm*" (Care Home 5), with one carer suggesting "*I can see a few hips going*" (Care Home 2). As a result of these concerns, participants felt they would not want residents using the treadmill "*without a staff member*" (Workshop 2).

Concerns of Negative Effects

Beyond some safety concerns of residents using a stand-up, slippery treadmill, broader concerns were raised around "*motion sickness*" (Care Home 3), feeling "*dizzy*" (Care Home 5), and unnatural movements "*because you're not stepping*" (Care Home 5), "*it'll feel a bit weird*" (Care Home 6).

Suitability/Unsuitability

Participants debated the older adults in their care who ROVR would and would not be best suited to. Based on the concerns presented above, care home staff echoed that residents would "*need to have much more support*" to use the treadmill (Care Home 3), and to be "*supervised*" (Care Home 2), which would mean "*we're going to have to get the staff on board*" (Workshop 2). The treadmill was felt to be more suitable for residents with "*more stability when they're on their feet*" (Care Home 5), while care staff listed diagnoses that would make the standing treadmill less suitable. Suitability concerns were raised for people with "*lots of medication [that] affects their balance*" (Care Home 1), people with "*dementia*" (Care Home 4), who could "*totally freak*" (Care Home 6) and people with "*vertigo*" (Care Home 1). Despite the concerns, participants acknowledged that "*just like anything else, it's time to get used to it*" (Care Home 2), suggesting that prolonged use may encourage more confidence.

Improvements

Although participants were generally accepting and appreciative of the concept of VR to motivate physical activity, and acknowledged the potential benefits, the stakeholders were

For peer review only - http://bmjopen.bmj.com/site/about/guidelines.xhtml

conscious of potential safety concerns which led to numerous participants suggesting a re-design of the treadmill to a "*seated unit*" which would "*be more beneficial to our client base*" (Care Home 2). Care staff suggested less mobile residents could be "*sat using their feet*" (Care Home 4) on a flat platform, to navigate the VR worlds without the risk of the standing, low-friction treadmill. Participants felt this would improve inclusivity of the experience as "*most [residents] come in in wheelchairs*" (Care Home 1). With these adaptations in mind, participants also shared thoughts on desired content for inclusion as a VR world, such as "*gardens*" (Care Home 4), "*under the sea*" (Care Home 5), or "*beaches*" (Care Home 4).

Current Design

While care stakeholders noted required adaptations to use VR to motivate movement and exercise safely for the target population of older adults and care home residents, participants praised aspects of the current design, including the VR content itself as "*amazing, brilliant, it is amazing, that is phenomenal*" (Care Home 5). They felt the headset "*doesn't feel too heavy on my head*" (Care Home 5), but "*feels really comfortable*" (Care Home 4). Participants did also note some technical glitches to be overcome, such as not "*going anywhere*" (Care Home 5) when walking, and "*flashing all over*" (Care Home 5). These forms of issue were reported to the developers for bug fixes during the study period.

Section 2: Results of the follow-up interview with the technology developer

Quotes have not been provided with an identifier as only one participant took part in this follow-up interview. Table 3 provides the main themes and initial codes and sub-codes resultant from analysis.

Table 3: The results of thematic analysis of the follow-up interview with the technology developer. Evidence to support each code is synthesised in narrative below.

Theme	Initial Codes, *Sub-codes*
Benefits	Beneficial outcomes; *Physical benefits, Social benefits, Mental benefits, Pushing personal boundaries, Benefits of Working with the University*
Process of development	Technology development; *Seated version*<br>Considering inclusivity; *Age, Care homes, Medical issues, Different sectors*<br>Phases of product development
Challenges	Cost and resources

Benefits

The follow-up interview with the technology developer highlighted a range of benefits born from the industry-academia collaboration. This included an initial code directly relating to *benefits of working with the University as* the participant reported it was "*really helpful*" through the opportunity to *"apply the minimum viable product [and figure] out where it works and where it doesn't"* stating *"you get really fast feedback."* The developer felt

For peer review only - http://bmjopen.bmj.com/site/about/guidelines.xhtml

academic collaboration and real-world feedback from stakeholders provided *“feedback we can work with.”* The participant stated: “*it’s really important for commerce [industry] to understand this, they need to work with people who are already experts*” as the participant felt universities *“have the freedom to do what most companies can’t.”* In this case, the technology developer benefited from the extensive experience the researchers had in working within social care and with older adults, *“you were working in this environment and already had that knowledge and understood the challenges involved.”* The developers felt bringing their specific technical knowledge and combining it with the university’s evaluation expertise meant “*we didn’t have to […] learn it ourselves […] or teach you […], all we had to do was provide the equipment and gain feedback.”* The developer felt having evaluation through the University provided a “*helpful,” “independent view” “which is unbiased.”*

The technology developer also noted benefits they perceived of the technology itself, based on the interactions during the study period and subsequent collaborative studies (to be published elsewhere). The developer discussed how older adults in general can need “*motivation to exercise*” with one particular challenge “*in the Northern hemisphere*” being “*winters [which are] long and wet and dark*” leading to older adults becoming “*deconditioned over the winter.”* Technologies such as ROVR provide an indoor solution to motivate physical activity, which could be achieved “*on a daily basis.”* The developer also shared the benefits of the potential social connection facilitated within the ROVR VR worlds, “*they [older adult] visited [cultural location in VR] before, they'd love to visit. They can meet up with friends, they don't have to be in the same location, they can join, without necessarily having VR equipment yeah. And that makes this a social interaction.”* Beyond the physical activity and potential for social contact, the developer felt they observed that the technology *“cognitively de-stressed*” older adults, meaning a range of potential benefits were observed.

Process of development

During the interview, the technology developer detailed how feedback from the current study helped lead to development of a seated version of the ROVR treadmill (ROVR Explore), which they felt was particularly required for “*the level of condition we found in care homes*” as “*they’re really seated […] without major assistance for a long time*” meaning they couldn’t use the standing ROVR. The participant stated: “*one of the limitations, […] is the inability to move around safely*.” The developer responded with “*a plate, which could be sat in front of people if they were seated and enable them to move.”* The participant stated: “*it was a real opportunity to include people who would otherwise be excluded*” demonstrating significant value in this insight and product development as a result of the collaboration.

Challenges

The technology developer also shared some thoughts on challenges they face generally, such as cost. Before 2012, VR development was only for those *“with a million pound budget*,” and though costs have fallen dramatically the feeling is that “*costs of development are still pretty high at present*” with “*various challenges around the world adding to those*

For peer review only - http://bmjopen.bmj.com/site/about/guidelines.xhtml

costs". Development costs and associated challenges further highlight the value of evaluation, iterative design and co-development with stakeholders, ensuring early developments are suitable and desirable for the intended audience before funds are wasted on inappropriate mass production.

Discussion

This study explored health and care staff perceptions towards the potential for VR motivated treadmill exercise for older adult care home residents, which is important due to the impact of the ageing population and growing burden of disease [2]. Research into the potential for technological innovations to support healthy ageing is essential to help respond to these challenges [3]. Our study adds to the literature on design of technology to encourage and support physical activity and exercise in older adults and care home residents.

Novel technologies such as the ROVR have potential to support healthy ageing. Our results demonstrate clear promise for such technologies to motivate and sustain increased physical activity in older adults and people living in care homes, with our large sample of care staff generally agreeing on potential benefits for exercise and activity. Implementation of products such as these would likely have benefits for meaningful activity, meeting the NICE [10] definition of providing an activity that promotes health and wellbeing through an activity with meaning to the individual, and is tailored to their preferences. Our results have demonstrated an interest and preference among stakeholders for cultural, heritage and natural sites for use as a meaningful VR experience. Further work is now required with older adults themselves to confirm their preference for such an activity.

Staff also perceived benefits for social interaction, through both the social aspect of the VR platform but additionally through increases in residents talking to each other, their families and carers with the technology experience providing a focal point. This could have implications for the experience of loneliness among the target population [5, 23]. Our results suggest the technology was also expected to allow for reminiscence and enhanced experiences, through allowing older adults and care home residents to 'visit' places and heritage sites they could no longer access. Staff also noted that older adults may be able to 'visit' places significant to them through memories from the past. The benefits of access to heritage locations are well documented [12], therefore future research could explore and document the impact virtual access that is paired with technology that involves exercise to move around heritage sites has on health and wellbeing outcomes.

However, despite the positive perceptions towards the concept of VR to motivate activity and use of some form of treadmill to encourage movement and muscle use, care staff raised concerns around product design, mostly related to safety. Participants were concerned that for the frailer older adults and care home residents, standing on the low-friction treadmill could present a falls risk. When results were provided to the technology developer, this study had immediate implications for product design. Based on this stakeholder feedback, the ROVR Systems team subsequently developed the Explore system, a seated version of the omnidirectional treadmill which would not present a falls risk while still motivating

For peer review only - http://bmjopen.bmj.com/site/about/guidelines.xhtml

activity and lower leg movement to build up leg strength. The seated Explore system is currently undergoing evaluation with stakeholders as part of the ongoing GOALD project [24]. Beyond this immediate effect, this study has broader implications for those designing physical activity technologies aimed at supporting aged care. It demonstrates that care stakeholders are open and accepting to such technologies, believing the concept to be positive, while also providing useful design, hardware, and content insight for future developments. This value is clearly demonstrated in this study through the follow-up interview with the developer, and subsequent new product development.

This study also reinforces previous work [1, 18], showing the importance of engaging stakeholders in the design and testing of technologies aimed at health and care. The follow-up technology developer interview showed the value of academic-industry collaboration. The developers were able to use these findings to adapt the technology into a seated treadmill, resulting in an enhanced safety experience for those unable to comfortably stand and to increase the accessibility of the experience and its potential health and wellbeing benefits.

Strengths and limitations

Our study achieved a relatively large sample for qualitative work and gained deep and thorough insight into care staff perceptions. Our analysis used rigorous methods and the topic of enquiry was novel, in exploring VR motivated physical activity specifically for older adult care home residents.

The strong qualitative approach produced data that was substantial and complex, and although the themes and categories presented here encompass all data, there is additional evidence of more profound and complex forms of engagement with VR technologies, such as speculative feedback or feelings of telepresence. These additional dimensions warrant further investigation.

There were two limitations. First, the duration for which participants interacted with ROVR was short. Our findings therefore may be over optimistic and influenced by the novelty effect. Longitudinal work would have better helped identify barriers and facilitators to use that would not be immediately recognised. The concerns of negative effects reported in our results, such as dizziness and motion sickness, would likely have been better assessed in longitudinal work, allowing time for participants to familiarise and overcome any initial negative sensations. These types of reactions are common among all VR systems, with work underway to generally reduce cybersickness experiences for users of head mounted displays [25]. Research has shown that repeated exposure to the same VR content will reduce severity of motion sickness [25], thus a longitudinal or repeated measures study would more accurately report on the degree of barrier presented by the unpleasant sensations. As with the potential reduction in motion sickness over time, participants themselves noted that it would take time to gain confidence in using the ROVR device with older adults and care home residents, therefore demonstrating potential to be more accepting over time.

Secondly, we only sought staff opinions in the potential future use of these devices, rather than older adults themselves. However, for devices such as these to be used by older adult

For peer review only - http://bmjopen.bmj.com/site/about/guidelines.xhtml

care home residents, as intended by the developers, they first need to be accepted and adopted by the staff team who would be responsible for buying and facilitating use. To overcome both the limitation of short interaction period and staff stakeholders only, we have conducted a 6-week implementation study with the ROVR device to be reported separately. It should also be considered that we did not explore device cost or site investment potential, therefore for real world implementations, cost would be a consideration.

Conclusion

The use of VR motivated exercise on an omnidirectional treadmill holds potential based on the perceptions of care staff for increasing exercise of older care home residents, while also providing virtual access to heritage sites, encouraging reminiscence and social connectivity, and promoting participation in a meaningful activity. A standing device however was not appropriate for a care home population and a new design is now being used for research with care home residents. User-centred design of health and care technologies is essential, and this study has demonstrated significant benefits for industry partners in collaborating with university institutions for independent evaluation with end-users.

Funding

This work was supported by UKRI Healthy Ageing Social, Behavioural and Design Research Programme grant number ES/V016113/1 as the project was completed by researchers on the GOALD project and the project also purchased ROVR equipment for the study, with further support from the EPIC (eHealth Productivity and Innovation in Cornwall and the Isle of Scilly) project which was part funded by the European Regional Development Fund. Additional funding for the EPIC project was received from University of Plymouth. ERDF Grant number [05R18P02814], as EPIC also purchased some of the ROVR equipment used. Finally, support was also provided by Innovate grant 10004423, which funded development, equipment and support provided by ROVR Systems Ltd.

Competing interests

The authors declare no competing interests.

Author Contributions

HB and KE led on the design of the research. HB, KE, LC, AW and RB conducted data collection. HB, LC, RB, AW, TK, ST, JR, GR, SG and AVR conducted data analysis. RJ oversaw the project design, completion and substantively contributed towards the write up, which was led by HB. SA, KW, CH, ACW, AC and RH oversaw study design and completion as GOALD senior management with RJ and reviewed and contributed towards the manuscript. All authors reviewed, edited and agreed to the final manuscript.

This project has received funding from the UKRI and ESRC Healthy Ageing Social Behavioural and Design Research Programme (SBDRP) grant No ES/V016113/1.
https://www.plymouth.ac.uk/research/centre-for-health-technology/goald

For peer review only - http://bmjopen.bmj.com/site/about/guidelines.xhtml

GOALD

Generating Older Active Lives Digitally

Healthy Ageing Challenge
Social Behavioural Design
Research Programme

UK Research
and Innovation

on behalf of the GOALD project: Catherine H. Hennessy, Faculty of Social Sciences, University of Stirling, Ray B. Jones, Faculty of Health, University of Plymouth, Richard Haynes, Faculty of Arts & Humanities, University of Stirling, Anna C. Whittaker, Faculty of Health Sciences & Sport, University of Stirling, (Core Management Team) and Sheena Asthana, Plymouth Institute for Health and Care Research (PIHR), University of Plymouth, Rory Baxter, Faculty of Health, University of Plymouth, Hannah Bradwell, Faculty of Health, University of Plymouth, Arunangsu Chatterjee, Faculty of Medicine and Health, University of Leeds, Pete Coffee, School of Social Sciences, Heriot-Watt University, Leonie Cooper, Faculty of Health, University of Plymouth, Alison Dawson, Faculty of Social Sciences, University of Stirling, Katie Edwards, Faculty of Health, University of Plymouth, Swen Gaudl, Faculty of Science & Engineering, University of Plymouth, Tanja Krizaj, Faculty of Health, University of Plymouth, Gregory Mannion, Faculty of Social Sciences, University of Stirling, Gemma Ryde, Institute of Cardiovascular and Medical Sciences, University of Glasgow, Alejandro Veliz Reyes, Faculty of Arts, Humanities and Business, University of Plymouth, John Ritchie, Faculty of Arts & Humanities, University of Stirling, Simone Tomaz, Faculty of Health Sciences & Sport, University of Stirling, Alison Warren, Faculty of Health, University of Plymouth, Karen Watchman, Faculty of Health Sciences & Sport, University of Stirling, Katherine Willis, Faculty of Arts, Humanities and Business, University of Plymouth, and partners - Active Stirling Ltd., Generations Working Together, Hearing Loss Cornwall, Nudge Community Builders, South Asian Society (Devon and Cornwall), Sporting Heritage, Sports Heritage Scotland, St. Breward Community, iSightCornwall, UKActive.

Acknowledgements

We would also like to thank our collaborating care homes for hosting our workshops and colleagues on the EPIC project who helped establish the collaboration.

Data sharing

Anonymised data is available from the corresponding author at reasonable request.

References

For peer review only - http://bmjopen.bmj.com/site/about/guidelines.xhtml

1. Moyle, W., Jones, C., Pu, L., & Chen, S.-C. (2018). Applying user-centred research design and evidence to develop and guide the use of technologies, including robots, in aged care. Contemporary nurse, 54(1), 1-3. doi:10.1080/10376178.2017.1438057

2. Abdi, J., Al-Hindawi, A., Ng, T. & Vizcaychipi, M.P. (2018). Scoping review on the use of socially assistive robot technology in elderly care. BMJ Open, 8(2), e018815.

3. UK Parliament (2018). Robotics in Social Care. Houses of Parliament, Post Note. [Internet] Available from: https://post.parliament.uk/research-briefings/post-pn-0591/

4. Garçon, L., Khasnabis, C., Walker, L., Nakatani, Y., Lapitan, J., Borg, J., et al. (2016). Medical and Assistive Health Technology: Meeting the Needs of Ageing Populations. Gerontologist, 56, Suppl 2:S293-302.

5. Siniscarco, M. T., Love-Williams, C., & Burnett-Wolle, S. (2017). Video Conferencing: An Intervention for Emotional Loneliness in Long-Term Care. Activities, Adaptation & Ageing, 41(4), 316-329. doi:10.1080/01924788.2017.1326763

6. Langhammer B, Bergland A, Rydwik E. The Importance of Physical Activity Exercise among Older People. Biomed Res Int. 2018 Dec 5;2018:7856823. doi: 10.1155/2018/7856823. PMID: 30627571; PMCID: PMC6304477.

7. Sherrington, C., Michaleff, Z. A., Fairhall, N., Paul, S. S., Tiedemann, A., Whitney, J., ... & Lord, S. R. (2017). Exercise to prevent falls in older adults: an updated systematic review and meta-analysis. British journal of sports medicine, 51(24), 1750-1758. http://dx.doi.org/10.1136/bjsports-2016-096547

8. To-Miles F, Mann J, Hung L (2022). Facilitators and barriers to using virtual reality and augmented reality and its impact on social engagement in aged care settings: a scoping review protocol. BMJ Open, 12, e061722. doi: 10.1136/bmjopen-2022-061722

9. ROVR Wizdish (2021). Step into a new reality: ROVR systems. [Online] [Accessed 4/11/2021] Available from: https://rovr.systems/

10. NICE (2013). Mental wellbeing of older people in care homes. Quality standard QS50. Available from: https://www.nice.org.uk/guidance/qs50/chapter/quality-statement-1-participation-in-meaningful-activity#:~:text=Meaningful%20activity%20includes%20physical%2C%20social,crafts%2C%20conversation%2C%20and%20singing. [accessed 13/02/23].

11. UNESCO (2021). Right to participate in cultural life. UNESCO. [Online] [Accessed 2/11/2021]. Available from: https://en.unesco.org/human-rights/cultural-life

12. Heritage Alliance (2020). Heritage Alliance Annual Report. [Online] Available from: https://www.theheritagealliance.org.uk/wp-content/uploads/2020/10/Heritage-Alliance-AnnualReport_2020_Online.pdf

13. Marasco, Alessandra & Balbi, Barbara. (2019). Designing accessible experiences for heritage visitors through virtual reality. e-Review of Tourism Research. 17.

For peer review only

For peer review only - http://bmjopen.bmj.com/site/about/guidelines.xhtml

14. Kużelewska, E., Tomaszuk, M. European Human Rights Dimension of the Online Access to Cultural Heritage in Times of the COVID-19 Outbreak. *Int J Semiot Law* (2020). https://doi.org/10.1007/s11196-020-09712-x

15. Paladini, A., Dhanda, A., Ortiz, M. R., Weigert, A., Nofal, E., Min, A., … Quintero, M. S. IMPACT OF VIRTUAL REALITY EXPERIENCE ON ACCESSIBILITY OF CULTURAL HERITAGE. ISPRS - International Archives of the Photogrammetry, Remote Sensing and Spatial Information Sciences, XLII-2/W11, 929–936. https://doi.org/10.5194/ISPRS-ARCHIVES-XLII-2-W11-929-2019

16. Dempsey, L., Murphy, K., Cooney, A., Casey, D., O'Shea, E., Declan, D., Jordan, F. and Hunter, A. (2014), 'Reminiscence in dementia: a concept analysis', Dementia, 13 (2), pp. 176-92, doi: 10.1177/1471301212456277

17. Smith, N., Towers, A-M., Plamer, S., Beecham J. & Welch, E. (2018). Being occupied: supporting 'meaningful activity' in care homes for older people in England. Cambridge University Press.

18. Bradwell, H.L., Edwards, K.J., Winnington, R., Thill, S. & Jones, R.B. (2019). Companion robots for older people: importance of user-centred design demonstrated through observations and focus groups comparing preferences of older people and roboticists in South West England. BMJ Open, 9(9), e032468.

19. Tong A, Sainsbury P, Craig J. (2007). Consolidated criteria for reporting qualitative research (COREQ): a 32-item checklist for interviews and focus groups, International Journal for Quality in Health Care, 19(6), 349–357. doi.org/10.1093/intqhc/mzm042

20. Braun, V., Clarke, V., Hayfield, N. & Terry, G. (2019). Thematic analysis. In Liamputtong, P. (Ed.). Handbook of Research Methods in Health Social Sciences. https://doi.org/10.1007/978-981-10-5251-4_103

21. Bonello, M., Meehan, B. Transparency and coherence in a doctoral study case analysis: reflecting on the use of NVivo within a 'Framework' approach. The Qualitative Report. 2019;24(3):483-98

22. Morse, J.M., Field, P.A. Qualitative research methods for health professionals. 1995.

23. Campaign to End Loneliness (n.d.). The facts on loneliness. [Online] Available from: https://www.campaigntoendloneliness.org/the-facts-on-loneliness/

24. University of Plymouth (2022) Generating Older Active Lives Digitally. [Online] Available from: https://www.plymouth.ac.uk/research/centre-for-health-technology/goald [accessed 01/03/23].

25. Chang, E., Kim. H.T. & Yoo, B. (2020) Virtual Reality Sickness: A Review of Causes and Measurements, International Journal of Human–Computer Interaction, 36:17, 1658-1682, DOI: 10.1080/10447318.2020.1778351

Figure legends

For peer review only - http://bmjopen.bmj.com/site/about/guidelines.xhtml

Figure 1: (Left) Care home staff member interacting with the ROVR Adventure treadmill with researcher recording initial reactions. (Right) VR headset with the ROVR Adventure omnidirectional treadmill and example of the app to access VR worlds

Figure 2: Example of the virtual museum participants could explore by walking on the ROVR Adventure

For peer review only

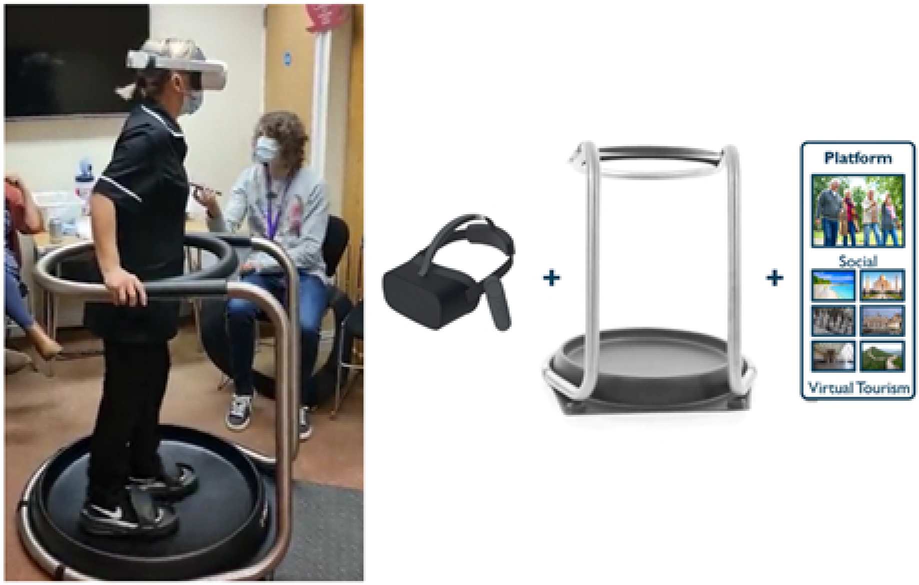

Figure 1: (Left) Care home staff member interacting with the ROVR Adventure treadmill with researcher recording initial reactions. (Right) VR headset with the ROVR Adventure omnidirectional treadmill and example of the app to access VR worlds

124x79mm (96 x 96 DPI)

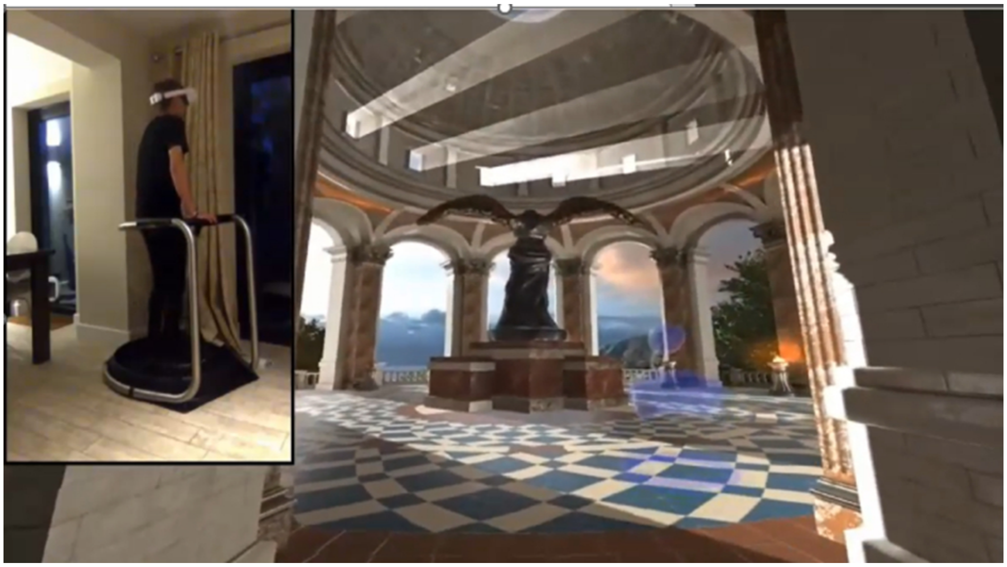

Figure 2: Example of the virtual museum participants could explore by walking on the ROVR Adventure

248x138mm (96 x 96 DPI)

Appendices

Appendix A –Workshop Interview Schedule

Site information

1) Please briefly describe the site you work at? (e.g. care home, extra care, supported living)

2) Please describe the number of residents or customers at that site and the level of care required? (e.g. physical ability of residents, prevalence of dementia)

Feedback on ROVR

1) What are your overall thoughts on the ROVR? How physically demanding did you find use of the ROVR?

2) What, if any, benefits do you perceive older adults might gain from using the ROVR?

3) What, if any, challenges might be encountered in the use of the standing or seated ROVR system with older adults (and care home residents)?

4) What characteristics do you think older people would need to use the standing and/or seated ROVR safely?

5) What challenges or health conditions do you think might make use of the standing and/or seated ROVR unsafe? (e.g. visual impairments, osteoarthritis, balance and coordination issues, frailty, mobility issues, support with grooming/communication/transfers/toileting/locomotion/self-care/communication *examples taken from the Functional Independence Measure*)

6) If the ROVR was in your care facility, do you envisage that it would change ways of working? (if so, how?)

7) Based on the video and demonstration you saw today, would you feel confident supporting use of the ROVR at your care facility? (further ideas on training requirements)

For peer review only

Appendix B – Supporting evidence for Table 2

Theme	Initial Codes, *Sub-codes*	Example Evidence
Anticipated benefits	Virtual visits	
	- *Interesting local sites*	“Eden project, Tintagel, Cornish mines, that kind of thing. That's the hope, and particularly post COVID with kind of reductions in mobility.” (Care Home 1)
	- *Virtual tourist sites*	“Some of these tourist sites to visit. So you could go to Venice or Paris.” (Workshop 1)
	Physical activity/exercise	“And it does exercise the top of the body as well. Yeah, obviously you need to push down. Yeah, take the weight off your feet when you slide. So it's, well, it is a whole body exercise really” (Care Home 5)<br>“You know, it's making each day have something new or useful or different, and this could open that to them. A massive, massive but as well as you know, without them realizing bit by bit and increasing their fitness, mobility, their longevity, their life span, their health” (Workshop 2)
	- *Motivating activity*	“And you know, struggle to get [exercise], how boring it is. If they can actually walk on that something to motivate them to do it. Yes, massive motivation isn't it. ” (Care Home 5)
	Prevents boredom	“Oh, it's good exercise as well as Yeah, it's actually giving them an experience. It's getting a mobile, perhaps someone that might just sit in their chair normally.” (Care Home 2)<br>“And [a resident] gets bored sometimes in the afternoon to the evening. And he's getting his coat or going out for a walk. Well, if we set him up on there. You could have a walk on here instead” (Care Home 5)
	Rehabilitation	“We had a bloke the other day that had a stroke. In the April he was discharged from hospital they couldn't do any more for him and he wasn't standing, wasn't walking. He went home from us because we, our staff were inputting exercise […] There was another lady […] But she was off her legs. And we've got her going and then she went home again. Yeah. If we can get close enough [to the ROVR] with the chair that they can actually sit and do it, it would be [rehabilitating].” (Care Home 5)<br>“Particularly I think it'd be fantastic for rehab. Absolutely. Brilliant. People go that have to walk up and down bars.” (Care Home 5)
	Staff use	“Yeah. even if our staff will be using it, our staff in front of our residents, makes good health and wellbeing staff and a morale boost. Yeah, because it's something new, something exciting that they can all talk about. So it's not just not just yeah, not just the service users, the staff as well as the whole well being.” (Care Home 5)
	Social interaction	“The novelty factor and the benefits of something different to what they've experienced in it, the talking points for themselves and their families that they probably can hang on to for quite a while.” (Workshop 1)<br>“I'm anticipating, essentially, social interaction, with the carers a bit more because there's going to be more communication? Yeah. So enhanced communication. Yes. Exercise. Yes. Yeah. Exercise. And interesting life.” (Care Home 5)<br>“[They can talk to each other] Yeah. I think that's the best part of it” (Care Home 2)
	Wellbeing	“So maybe in time, it would be nice to involve the communities in and around our houses.” (Workshop 2)<br>“Ultimately, if they're using it, their well being is completely going to change and they're going to be happier.” (Care Home 1)<br>“But I think for mental health it would be good” (Care Home 2)
	Accessible experiences	“So some are just not able to get out anymore. Yeah. So if you're providing the opportunity to see the place that they love” (Care Home 4)
	- *Different to care home*	“If you're living in a care home, to go to the different, that environment was lovely.” (Workshop 1)
	Stimulates the brain	“Stimulates the brain. Yeah. Got me thinking.” (Care Home 1)
	Restorative	“that will bring their confidence back” (Workshop 2)
	Reminiscence	“Especially if they're walking in an area they know. They are walking down. Trigger memories” (Care Home 2)

For peer review only

	- *Reminiscence benefits*	"Well, I think it's memories, it's reminiscing for them, isn't it? If they went to it's like, you know, bearing in mind not everybody that lives here is from Devon or Cornwall, we've got people that have moved from the land at some point. So even to have those places it brings back memories. You know, we've got old photographs of Saltash, pictures of Saltash, and that, you know, they'll say, Oh, we went to that cinema when I was young. You know, it brings back memories. So I'm presuming this will do the same. I'll show them because yeah, A lot of living with dementia. They're living years ago." (Care Home 4)
	Expectations challenges positively	"That was alright actually, I didn't think, it wasn't as bad as I thought it was going to be. I felt more in control than I thought I would." (Care Home 2)
	Positive distraction and novelty	"What we were saying, and you know you said how was things I think, you know, people at the moment are feeling so overwhelmed. Yeah, we've been through something like this would take their minds off things" (Care Home 2)
Acceptability	Positive comments	"Yeah, that's amazing. Absolutely amazing" (Care Home 3)<br>"I honestly think this is amazing" (Care Home 6)
	ROVR is interesting	"Interesting idea." (Workshop 1)
	Adoption desired	"I would […] buy it for the staff and residents. I would consider that, I would put it before my board so that we actually had one permanently." (Care Home 5).
	Ease of use	"Easy to control" (Care Home 5)
	- *Headset initially heavy but comfortable*	"It feels a little bit heavy forward so I've got to bring it forward, but it is good." (Workshop 1)
	- *Physically easy*	"Not for me intense at all." (Workshop 1)
	- *Treadmill is easy to enter*	"In felt fine [getting in and out], because at least I had one stable foot on the ground, and sort of the bars were in front of me." (Workshop 1)
	- *Treadmill is easy to move on*	"I actually find the walking on it much easier than I thought it would be to be." (Workshop 1)
	- *VR is novel experience*	"One [benefit], the novelty factor and the benefits of something different to what they've experienced in it." (Workshop 1)
Concerns of use	Secureness	"That does feel more secure, leaning on the bars around you. And it helps to keep you stable." (Care Home 3)
	Safety concerns	"Yeah, just whilst they still think they can walk. And while that seems pretty easy, yeah, that potentially is gonna make them really unstable. Or they'll get breathless really quick" (Care Home 3)<br>"It's unsafe I would say as strong as that they would be unsafe" (Care Home 4)
	- *Hitting objects*	"... Oh, I've got a move in a minute, because I'm going to hit, say the bollard, I already hit it. And I wanted to go up the stairs. But I couldn't go upstairs because I'd hit it already..." (Care Home 1)
	- *Shoes*	"The shoes are going to be a little bit of an issue. If they can come in different sizes that might be But the smaller they are, the tighter they're going to be. So I found that quite tight to fit the one large and I've only got size three feet. Yeah. very slippery to stand on. And I can see that being quite an issue" (Care Home 1)
	- *Safety hazards*	"Maybe you need something around the outside. So if they fell, they just they can't come out. They can only go on their knees then can't they?. Do you know what I mean? If there was a guard? Actually, because that's my worry if someone fell out of that." (Care Home 5)<br>"You see that wouldn't be suitable for a resident. That is too slippery" (Care Home 4)<br>"That's that's a slippery slope." (Workshop 2)
	- *Danger*	"They [residents] can't afford to fall, if they fall it's gonna be an injury isn't it" (Care Home 6)<br>"It's a bit slippery to get on and off? That is a danger" (Care Home 5)
	Lack of control	"It's it's it's fast and then it's because you can't control it" (Care Home 3)

For peer review only - http://bmjopen.bmj.com/site/about/guidelines.xhtml

	- *Movement issues*	“I'm not really moving anywhere.” (Workshop 1)
	Risk of falls/Dangerous	“more support for them because the type of people that we get like I've said are not as agile as they used to be even though sometimes they think they are and they're potentially going to fall in that.” (Care Home 3)
	- *History of falls*	“I don't know that she'd be happy to do it. Because she's got a history of falls. Okay, so she’d probably not enjoy it.” (Workshop 1)
	- *Could cause falls*	“I think when it could actually make someone fall.” (Workshop 1)
	- *Risk of injury – hips*	“If you slipped now, you're gonna bang yourself on those bars, then you're gonna catch your ribs or whatever? Or your arm, break an arm on there if you go down, yeah.” (Care Home 5)<br>“Yep. Okay. I can see a few hips going.” (Care Home 2)
	Accessibility	“Getting in was alright, getting out was difficult, because you've already done that slippery surface” (Care Home 3)<br>“You could do with like a virtual reality wheelchair, they sit down and go, around.” (Care Home 5)
	Prefer people go out	“the deterioration that we've seen in so many of our residents is tenfold and I'd rather get them out. But then you have that knock on effect that the families don't want to. But if they have the option of liaising via a website, let's go and visit Eden with her” (Care Home 1)
	Heavy shoes	“Yeah, the heaviness of those shoes, and being nervous of falling.” (Workshop 1)
	Lack of motivation	“Definitely. Yeah. There's lots of ‘Mister, come on, you can do it. You can do it.’, ‘I don't want to do it’." (Workshop 1)
	Uncomfortable movement	“I feel like the twisting itself, which is where the uncomfortable sensation perhaps maybe lies.” (Workshop 1)
	Bigger environment more physically demanding	“Depends on how big the world is, because this world feels small and so I feel like I have to twist.” (Workshop 1)
	Critique	“I wouldn't want them using it without a staff member on the site.” (Workshop 2)<br>“Resident would be screaming the place down, and panic about the step [into ROVR]” (Care Home 6)
	Potential issues	“this idea of being in an open space or field might be disorientating” (Workshop 2)
Concerns of negative effects	Side effects	“Yeah. It wasn't motion sickness but I just started to feel like I just need to take this off now, yeah, it's just a really strange feeling” (Care Home 3)<br>“Yeah, I've got a feeling of like, yeah, maybe I've got like a bit of car sickness.” (Care Home 4)
	- *Encouraging shuffling/sliding*	“That is almost the encouraging of Parkinson's type shuffling isn't it?” (Workshop 1)
	- *Unnatural movement*	“As you move around this change of texture to this and that makes it seem like it's not as organic or not as natural” (Workshop 1)<br>“A bit weird, because you’re not stepping” (Care Home 5)<br>“Keep sliding […] it’ll feel a bit weird” (Care Home 6)
	Dizziness	“I might feel dizzy. It was alright there but when you go off the steps and stuff you start getting a bit. It's because when you went the faster you go. Then when you turn that's when you get a bit. Isn't it like discombobulated?” (Care Home 5)<br>“It feels really weird, my head is still spinning” (Care Home 6)
	Disorientating	“That’s amazing but it messes with your head” (Care Home 3)<br>“Trying to orientate yourself is quite difficult. Yeah. Yeah. Especially around the house.” (Care Home 1)<br>“Especially when you got the headset on, you're disorientated as well.” (Care Home 4)
	- *Orientating to environment*	“The only thing that concerns me is the disorientation that. Yeah, that's the only thing. Okay, Whether they'd be disorientated.” (Care Home 2)<br>“Yeah, I think it'd be really hard because you lose all your visual cues like even though I've got no balance problems it was pretty tricky. You do certainly do lose your orientation before you put those on, like, I didn't know where anyone was. Deal with the disorientation and the

For peer review only - http://bmjopen.bmj.com/site/about/guidelines.xhtml

		nausea. Anyway, which they might not be able to tell you about, so I'd be worried about it for dementia. Without a lot of, it's worth trying." (Workshop 1)
	- *Coordination between VR and feet*	"Yeah, since you've had the headset on your feet don't seem in momentum. Is it difficult to watch and organise your feet?" (Care Home 4)
	- *Height changes*	"It [*disorientation*] didn't affect me till there was like the change in height" (Workshop 1)
	- *Motion*	"Motion is kind of backwards and forwards, like you're on a cross trainer." (Workshop 1)
	- *Social angle confusing*	"For a few? Most of them I would say it [social experience in VR] would be very confusing." (Workshop 1)
	Concentrating more on the exercise	"Concentrating more on the exercise." (Workshop 1)
	Depth perception difficulties	"Because it is such a such a strange sensation of a lot of different coordination at the same time, and that the depth perception issues" (Workshop 1)
	Motion sickness in VR	"Oh, I'm actually feeling really dizzy." (Workshop 1)<br>"I felt motion sickness when I got off yeah. But I think it was too enclosed. Like, there's too many walls and things. I hit a wall quite quickly," (Care Home 4)
	Fear	"I think some elderly people would find it quite scary. Yeah, I do." (Care Home 1)<br>"They [residents] might feel a bit scared, because because it's slippy." (Care Home 2)
Suitability/Unsuitability	Extra support	"I think the worry for me is is if we get to the stage where the residents go in there. Yeah, I think we need to have much more support for them" (Care Home 3)<br>"I don't think I'd feel happy start with if they weren't being supervised." (Care Home 2)
	- *Supervised use required*	"Yeah, but you still need to hold on it. But I think what you would always do with the residents on that, or you would always have one person here" (Care Home 5)
	- *Guidance and guided navigation*	"I was just saying, if your staff can't see what's going on, when you run into problems, they can't troubleshoot it for someone who's struggling cognitively, or even just unfamiliar with it, like, am I on the balcony? Do I need to get to it? If I'm not on the balcony, how do you guide someone up to it? What do I do? They can't then put the mask on, with infection, you can just put the mask on and check, you've got to take someone's headset off." (Workshop 1)
	- *Human resources*	"I do think it will need some supervision, so definitely, we're going to have to get the staff on board with it. Because well, I, it's not something that I would expect to walk in at nine o'clock in the morning and see someone on it without one of us being there" (Workshop 2)
	- Number of residents	"they all they've all got walking frames and need wheelchairs. And even that's residential. I would say only a couple." (Care Home 1)<br>"I can think of one called [resident name]. Yeah, that's about the only one I will allow would feel comfortable with." (Care Home 4)<br>"Obviously you just have to pick the residents that would be appropriate for, because someone with mental health issues would really struggle on this." (Care Home 4)
	- *Differences between residents*	"[Suitable for residents with] More stability when they're on their feet" (Care Home 5)
	- *Residents unable to use*	"I don't know if a resident would be able to do that" (Care Home 1)
	Diagnosis	"Imagine that somebody with cognitive impairment or a spatial awareness issue, poor eyesight, mobility wise, […] not somebody of age (Care Home 6)

For peer review only - http://bmjopen.bmj.com/site/about/guidelines.xhtml

	- *Balance*	"I think it's the overall, you know, they are on lots of medication, how that's going to affect their balance. I've got some people who have vertigo, they wouldn't be able to do it." (Care Home 1)
	- *Dementia/cognition*	"First, immediately, people are just gonna let go. Okay, especially if they got mild dementia and something like that" (Care Home 1)<br>"And of course they are in a not in a reality situation. So they don't know actually where they are." (Care Home 5)<br>"Has anyone said about the issue about with the mental health. They might think why are here? Yeah. Why are we keeping them from them. There's some here that would love to be in Australia with the granddaughter and they would fully understand that it's a Virtual world and also coming back out and leaving that experience then suddenly the grandchild is gone again. Someone living with dementia only got to see somebody crossing the car park, and they think it's one of their family and they think why do you not let them in, it's not their family but they think." (Care Home 4)<br>"I feel like it would totally freak our clients [...] I think better cognition" (Care Home 6)
	- *Fear of falling*	*Interviewer:* "Yeah. Do you think that that slipperiness would be a problem? "<br>*Participant:* "It would be for some of our residents here. Yeah. Yeah. They would just fall over" (Care Home 1)<br>"Just what we've already said, totally inappropriate for residents because if they fall." (Care Home 4)
	- *Have to be able*	"They've got to be quite able. So our most able person is is she's done the, the other one with us. She goes out gardening, she goes out doing lots of things. But she's had a fall, because she was pulling weeds out and one was too strong. So it can easily be done. That then will knock her confidence. So I don't want her going on something like this that she wasn't that keen. Yeah. Okay. So they have to be very able" (Care Home 1)<br>"In fact, they because I've had to hold here. They would have to hold on really tight. Yeah. And because they're older this the grip alone." (Care Home 4)
	- *Incontinence*	"So you might have to look around that a little bit. Yeah. Especially with people that are already afraid of falling. Yeah, they're incontinent because they fall. They don't want you know, they don't want to go to the toilet, because so going on something like this might be a little bit of an issue." (Care Home 1)
	- *Influence of medication*	"I think it's the overall, you know, they are on lots of medication, how that's going to affect their balance. I've got some people who have vertigo, they wouldn't be able to do it" (Care Home 1)
	- *Limited mobility*	"This bit as well. I mean, we've got here they're not very mobile or anything." (Care Home 1)<br>"We're got a few that are quite mobile. Even the ones that could walk I think would really struggle." (Care Home 5)
	- *Problems with vision*	"A little bit. Getting better but it's still quite blurry though." (Care Home 4)
	- *Neck pain*	"I was like that well, going around rather than like that, that would that give you the neck neck ache and then that, okay, posture? Yeah" (Care Home 1)
	- *Neuromuscular*	*Interviewer*: "So thinking about maybe some of your family members, then what kind of conditions or challenges do you think a person would have that would mean this wasn't suitable?"<br>*Participant:* "Neuromuscular degeneration is one for me." (Workshop 1)
	- *Lived problem*	"would that be important that actually there was an ongoing service? Rather than you buy this and you're left on your own? Would you prefer it that actually, perhaps I pay a monthly fee? And I know, I can always pick up the phone if something goes wrong and contact that company? Would you rather kind of buy it and own it? Or have that kind of that long term rental maintenance and the service that comes with it?" (Workshop 2)

For peer review only

	- *Vertigo*	"For someone with that impairment, or someone with that illness, that's just not suitable for them. What about someone with vertigo?" (Care Home 2)
	Suitability for residents	"What do you think with the residents? I just I just said there's not many that we'll be able to put on that." (Care Home 5)
	- *99% not suitable*	"99% won't be able to do it anyhow" (Workshop 1)
	- *Suitable for residents 20 years younger*	"Twenty years ago when we had residents, like you can think we still got residents that would have been fine but now. No way" (Workshop 1)
	- *Wouldn't recommend to people with mobility issues*	"Unsteady gait in general, having vertigo, history of, history of falls in any sense, increasing that risk of. I wouldn't bother I think." (Workshop 1)
	Ethics	"There's still challenges, though, isn't there of getting on and off like exercise, you know, I've taken loads of people on it, like that, it can be quite a dodgy thing to do. It's not okay, so yeah, there's still challenges with getting older adults on and off these things, especially anyone who has cognitive impairment," (Workshop 1)
	Risk	"Older adults wouldn't be able to even step over that even though she's on it [...] And probably in nursing homes older people's standing tolerance may not be that. And the effect on balance from turning, as well as the nausea point of view" (Workshop 1)
	Size of technology	"It is massive. I didn't expect it to be this big." (Care Home 1)
	- *Wifi connectivity*	"It would probably be in the lounge because probably we get quite good reception in the lounge. Sometimes in parts of the building we might lose it at times." (Care Home 4)
	Familiarisation	"And just like anything else, it's time to get used to it. That familiarization with. But that's like everything that we have that's new. Once people are familiar with it, and it's normal. I mean, to our residents, this sort of technology is quite strange, anyway," (Care Home 2)
	- *Adapting to use*	"Because I'm, I've got a bad back. But I was able to still able to do it. I got poor eyes because I've got wet macular degenerate disease but it was fine. Okay, I couldn't do as long as what [other participants] did probably, but also it's my first time in VR so I was cautious but as you get used to it." (Care Home 5)
	- *Unfamiliar*	"I can just see a pillar here. I could hear someone it's like the pillars talking [...] It's like I'm disorientated" (Care Home 5)
	- *Adjusting to headset*	"And when the VR went on, very disorientating, very blurry, and for then to adjust it as they want it, I think might be an issue. So you need to be able to see what they're seeing on the computer to you maybe have to adjust it that" (Care Home 1)
	- *Familiarity*	"I certainly think the fact that you're going to have destinations, local destinations will be the attraction." (Workshop 2)
Improvements	Content suggestions	"Under the sea would be good wouldn't it?" (Care Home 5)<br>"We could take them to places. If we could get in the gardens every year. If you could capture that." (Care Home 4)<br>"Because you really you're taking them to places you know like the Beaches."(Care Home 4)
	- *Cartoon-like images*	"You want to touch the flowers, so they will let go and then they'll Yeah, yeah. So I do think that's a bit of a challenge mobility, and that's quite cartoonish as well. So will it be reality?" (Care Home 1)
	- *New environments*	"Yeah, Mount Edgecumbe. All those kinds of things that they they would love. Even the Tamar Bridge. A garden center. You know, last gardens of Helligen, Eden Project. I think they'd love it. Yeah, I think they'd really love it. And they're sitting down and they can look at the flowers. And, you know, pick, they can do all these kinds of things. But if they're standing up, I think there'll be more "I don't like it. I don't like it", because it's do you see what I mean? Yes. Yeah," (Care Home 1)
	Inclusivity	"how we could adapt the equipment for her typically she has full capacity and would really benefit from this but obviously it's a double amputee so she's not going to physically walk" (Care Home 3)
	- *Adaptable bar height*	"Yeah, even if that was that was, you could undo it and take that bit off. You only need an eye and a button don't you like you don't like the walking sticks with the eyes and the button you only need a click button and then you could make it different sizes as well. It could go back further, be adaptable. They have something to hold on to in front of them." (Care Home 5)

For peer review only - http://bmjopen.bmj.com/site/about/guidelines.xhtml

	- *Fitting to a wheelchair*	"Even if it's just like a normal chair. Yeah. Not a wheelchair. Because when you soon as you get in a wheelchair, you think you're going somewhere, which might be a good thing." (Care Home 1)
	- *People less mobile*	"You know, most of them come in in wheelchairs. Okay. That's the way residents are coming in." (Workshop 1)
	- *Access could be improved*	"You have to naturally be able to step up, don't you? So if you could step up onto like, just a something there, as a gap in between." (Workshop 1)
	- *Physical requirements*	"From a physical perspective. I have to say this is a dance of what they have been used to before, but I would think that whoever would need to, to safely do this would already have a degree of practicality about them." (Workshop 1)
	Safety improvements	"There's that feeling of going back. So I think if there were two front legs and at the back, that would give much more support?" (Care Home 3)<br>"I think you're looking at someone with a degenerative physical disease, but good cognition, once you've made some modification, so it doesn't kill them." (Workshop 1)
	- *Softer impact bars*	"If they do, if a resident slips out, as in legs come down. Obviously, we would need some sort of buffers over the top of the bars," (Care Home 5)
	- *Stop slipping*	"I don't know, do you need to have it slippery actually." (Care Home 5)
	- *Awfully slippery*	"Awfully slippery. So you're, you're like when you're walking on ice or wherever you're on guard. So that takes away the enjoyment." (Workshop 1)
	- *Difficulty exiting treadmill*	"I'm not sure how many people would be able to get out of that." (Workshop 1)
	Add arm movements	"You have like any interaction with hand stuff as well. You know you've got your feet Yeah, you do. So you have things in your hands so" (Care Home 5)
	Cast visual for group to watch	"Yeah. I don't know if we could connect to this screen, so the rest of them can actually see on the screen where this [in the VR] is." (Care Home 5)
	Seated version	"The seated unit would be more beneficial to our client base, as we are at the moment." (Care Home 2)<br>"It would have to be a sitting one, but would they like that [VR headset] on their head" (Care Home 6)
	- *Square*	"If it was sitting down. Yeah. Yeah, that would be different." (Workshop 1)
	- *Headset use only*	"I would think if it was less slippery, yeah. And maybe if you could, well, you couldn't just use the headset. Could you? Could you just use the headset?" (Care Home 1)
	- *Adaptations*	"Could they not sit? If they were sat using their feet Yeah. But then they wouldn't get do you have to. Is there a was a chair scenario." (Care Home 4)
	Possibilities/exercise bike	"I wonder about it on a bike because that way. You've got your bike, which turns, so pedalling is innate. People know peddling goes forwards. And so on. And if you could turn on that. I wonder if it would keep you a little bit more orientated? Because all your movement has been done with that and with the steering wheel aspect to that. And so I don't know if that would be better. If you're trying to get the exercise. Cause it's really interesting. You're looking at it from the exercise point of view. If it's just getting someone out and about." (Care Home 5)
Current design	Good content	"I'm just getting to the balcony. Oh my god. Oh that is amazing brilliant. It is amazing that is phenomenal." (Care Home 5)<br>"But it just feels so real" (Care Home 4)<br>"It's beautiful" (Care Home 6)
	- *Visual features*<br>- *Spatial features*	"No, I don't want to go down the steps. I'm gonna go this way. I don't want to go down the steps, I don't like it" (Care Home 4)
	Liked headset	"It doesn't feel heavy on my head" (Care Home 5)

For peer review only

		“Yeah, that's good, it feels really comfortable” (Care Home 4)
	Safety features	“That’s obviously why the bars are there” (Care Home 5)
	Technology issues	“However much walking I got I wasn't going anywhere.” (Care Home 5)
	- *Glitching*	“You’re actually flying all over the place […] you are there but you’re flashing all over it as well.” (Care Home 5)
	- *Loading worlds*	“All I got is clouds and a green line. It's just a green line.” (Care Home 5)
	- *Speed of movement*	“I'm guessing I would say that one slide of that foot? You'd probably moving about two feet.” (Care Home 5)
	Ergonomics	“I'm stepping in a way I wouldn't normally step” (Care Home 5)<br>“They didn't feel big on the feet. Because you're sliding” (Care Home 4)
	Mechanics	“It doesn't feel like natural walking.” (Workshop 2)
	Resolution	“it's going to feel a lot more stable with that in there, and so they've suggested that when people have their first experiences, it's always with the training mats in place.” (Workshop 2)

For peer review only

For peer review only

For peer review only - http://bmjopen.bmj.com/site/about/guidelines.xhtml

COREQ (COnsolidated criteria for REporting Qualitative research) Checklist

A checklist of items that should be included in reports of qualitative research. You must report the page number in your manuscript where you consider each of the items listed in this checklist. If you have not included this information, either revise your manuscript accordingly before submitting or note N/A.

Topic	Item No.	Guide Questions/Description	Reported on Page No.
**Domain 1: Research team and reflexivity**			
Personal characteristics			
Interviewer/facilitator	1	Which author/s conducted the interview or focus group?	
Credentials	2	What were the researcher's credentials? E.g. PhD, MD	
Occupation	3	What was their occupation at the time of the study?	
Gender	4	Was the researcher male or female?	
Experience and training	5	What experience or training did the researcher have?	
Relationship with participants			
Relationship established	6	Was a relationship established prior to study commencement?	
Participant knowledge of the interviewer	7	What did the participants know about the researcher? e.g. personal goals, reasons for doing the research	
Interviewer characteristics	8	What characteristics were reported about the inter viewer/facilitator? e.g. Bias, assumptions, reasons and interests in the research topic	
**Domain 2: Study design**			
Theoretical framework			
Methodological orientation and Theory	9	What methodological orientation was stated to underpin the study? e.g. grounded theory, discourse analysis, ethnography, phenomenology, content analysis	
Participant selection			
Sampling	10	How were participants selected? e.g. purposive, convenience, consecutive, snowball	
Method of approach	11	How were participants approached? e.g. face-to-face, telephone, mail, email	
Sample size	12	How many participants were in the study?	
Non-participation	13	How many people refused to participate or dropped out? Reasons?	
Setting			
Setting of data collection	14	Where was the data collected? e.g. home, clinic, workplace	
Presence of non-participants	15	Was anyone else present besides the participants and researchers?	
Description of sample	16	What are the important characteristics of the sample? e.g. demographic data, date	
Data collection			
Interview guide	17	Were questions, prompts, guides provided by the authors? Was it pilot tested?	
Repeat interviews	18	Were repeat inter views carried out? If yes, how many?	
Audio/visual recording	19	Did the research use audio or visual recording to collect the data?	
Field notes	20	Were field notes made during and/or after the inter view or focus group?	
Duration	21	What was the duration of the inter views or focus group?	
Data saturation	22	Was data saturation discussed?	
Transcripts returned	23	Were transcripts returned to participants for comment and/or	

For peer review only

Topic	Item No.	Guide Questions/Description	Reported on Page No.
		correction?	
**Domain 3: analysis and findings**			
Data analysis			
Number of data coders	24	How many data coders coded the data?	
Description of the coding tree	25	Did authors provide a description of the coding tree?	
Derivation of themes	26	Were themes identified in advance or derived from the data?	
Software	27	What software, if applicable, was used to manage the data?	
Participant checking	28	Did participants provide feedback on the findings?	
Reporting			
Quotations presented	29	Were participant quotations presented to illustrate the themes/findings? Was each quotation identified? e.g. participant number	
Data and findings consistent	30	Was there consistency between the data presented and the findings?	
Clarity of major themes	31	Were major themes clearly presented in the findings?	
Clarity of minor themes	32	Is there a description of diverse cases or discussion of minor themes?	

Developed from: Tong A, Sainsbury P, Craig J. Consolidated criteria for reporting qualitative research (COREQ): a 32-item checklist for interviews and focus groups. *International Journal for Quality in Health Care*. 2007. Volume 19, Number 6: pp. 349 – 357

Once you have completed this checklist, please save a copy and upload it as part of your submission. DO NOT include this checklist as part of the main manuscript document. It must be uploaded as a separate file.

For peer review only

BMJ Open

Staff perceptions towards virtual reality motivated treadmill exercise for care home residents: A qualitative feedback study with key stakeholders and follow-up interview with technology developer.

Journal:	*BMJ Open*
Manuscript ID	bmjopen-2023-073307.R1
Article Type:	Original research
Date Submitted by the Author:	13-Jul-2023
Complete List of Authors:	Bradwell, Hannah; University of Plymouth<br>Cooper, Leonie; University of Plymouth<br>Edwards, Katie; University of Plymouth, Faculty of Health<br>Baxter, Rory; University of Plymouth<br>Tomaz, Simone; University of Stirling<br>Ritchie, John; University of Stirling<br>Gaudl, Swen; University of Plymouth<br>Veliz-Reyes, Alejandro; University of Plymouth<br>Ryde, Gemma; University of Stirling<br>Križaj, Tanja; University of Plymouth<br>Warren, Alison; University of Plymouth<br>Chatterjee, Arunangsu; University of Plymouth, Centre of Health Technology; University of Leeds, School of Medicine<br>Willis, Katharine; University of Plymouth<br>Haynes, Richard; University of Stirling<br>Hennessy, Catherine; University of Stirling<br>Whittaker, Anna; University of Stirling<br>Asthana, Sheena; University of Plymouth<br>Jones, Ray; University of Plymouth
**Primary Subject Heading**:	Qualitative research
Secondary Subject Heading:	Geriatric medicine, Health informatics
Keywords:	Virtual Reality, Physical Therapy Modalities, Information technology < BIOTECHNOLOGY & BIOINFORMATICS

SCHOLARONE™
Manuscripts

I, the Submitting Author has the right to grant and does grant on behalf of all authors of the Work (as defined in the below author licence), an exclusive licence and/or a non-exclusive licence for contributions from authors who are: i) UK Crown employees; ii) where BMJ has agreed a CC-BY licence shall apply, and/or iii) in accordance with the terms applicable for US Federal Government officers or employees acting as part of their official duties; on a worldwide, perpetual, irrevocable, royalty-free basis to BMJ Publishing Group Ltd ("BMJ") its licensees and where the relevant Journal is co-owned by BMJ to the co-owners of the Journal, to publish the Work in this journal and any other BMJ products and to exploit all rights, as set out in our licence.

The Submitting Author accepts and understands that any supply made under these terms is made by BMJ to the Submitting Author unless you are acting as an employee on behalf of your employer or a postgraduate student of an affiliated institution which is paying any applicable article publishing charge ("APC") for Open Access articles. Where the Submitting Author wishes to make the Work available on an Open Access basis (and intends to pay the relevant APC), the terms of reuse of such Open Access shall be governed by a Creative Commons licence – details of these licences and which Creative Commons licence will apply to this Work are set out in our licence referred to above.

Other than as permitted in any relevant BMJ Author's Self Archiving Policies, I confirm this Work has not been accepted for publication elsewhere, is not being considered for publication elsewhere and does not duplicate material already published. I confirm all authors consent to publication of this Work and authorise the granting of this licence.

For peer review only

Staff perceptions towards virtual reality motivated treadmill exercise for care home residents: A qualitative feedback study with key stakeholders and follow-up interview with technology developer.

Bradwell, H.L[1*]. Cooper, L[1]., Edwards, K.J[1]., Baxter, R[1]., Tomaz, S.A[2]., Ritchie, J[2]., Gaudl, S[1]., Veliz-Reyes, A[1]., Ryde, G.C[2]., Krizaj, T[1]., Warren, A[1]., Chatterjee, A[1 3]., Willis, K[1]., Haynes, R[2]., Hennessy, C.H[2]., Whittaker, A.C[2]., Asthana, S[1]., Jones, R.B[1] on behalf of the GOALD project.

[1] Centre for Health Technology, University of Plymouth, Plymouth, UK.

[2] University of Stirling, Stirling, UK.

[3] School of Medicine, University of Leeds

*Corresponding author: Hannah.bradwell@plymouth.ac.uk

For peer review only

Abstract

Objectives
Health and care resources are under increasing pressure, partly due to the ageing population. Physical activity supports healthy ageing, but motivating exercise is challenging. We aimed to explore staff perceptions towards a virtual reality (VR) omnidirectional treadmill (ROVR), aimed at increasing physical activity for older adult care home residents.

Design
Interactive workshops and qualitative evaluation.

Settings
Eight interactive workshops were held at six care homes and two university sites across Cornwall, England from September-November 2021.

Participants
Forty-four staff participated, including care home, supported living, clinical care and compliance managers, carers, activity coordinators, occupational therapists, and physiotherapists.

Interventions
Participants tried the VR treadmill system, followed by focus groups exploring device design, potential usefulness, or barriers for care home residents. Focus groups were audio recorded, transcribed verbatim and thematically analysed. We subsequently conducted a follow-up interview with the technology developer (September 2022), to explore feedback impact.

Results
Analysis produced seven key themes: anticipated benefits, acceptability, concerns of use, concerns of negative effects, suitability/unsuitability, improvements, and current design. Participants were generally positive towards VR to motivate care home residents' physical activity and noted several potential benefits (increased exercise, stimulation, social interaction, and rehabilitation). Despite reported potential, staff had safety concerns for

frail older residents, due to the standing position. Participants suggested design improvements to enhance safety, usability, and accessibility. Feedback to the designers resulted in development of a new seated VR treadmill to address concerns on falls while maintaining motivation to exercise. The follow-up developer interview identified significant value in academia-industry collaboration.

Conclusion

The use of VR motivated exercise holds potential to increase exercise, encourage reminiscence, and promote meaningful activity for care home residents. Staff concerns resulted in a re-designed seated treadmill for those too frail to use the standing version. This novel study demonstrates importance of stakeholder feedback in product design.

Key words: social care, physical activity, technology, virtual reality, innovation, knowledge exchange

Word count: 4342

Article Summary

Strengths and limitations of this study

- Relatively large sample of participants for qualitative research providing breadth of perceptions and data saturation
- Rigorous qualitative analysis on novel topic by a research team with diverse (yet, related to the topic) disciplinary backgrounds (including engineering, health, design)
- Explored perceptions of key stakeholders (health and social care staff)
- However, limited interaction durations between participants and the technology
- No perceptions gathered from older adult stakeholders

Background

Introduction

Pressure on health and social care (H&SC) resources is increasing, because of increased life expectancy and decreasing numbers of care workers [1, 2]. Therefore, there has been a push for research and innovation into technologies to support healthy ageing [3, 4]. Reducing loneliness and remaining active are key strands of improved wellbeing for older adults [5, 6]. Physical activity has numerous health benefits for older adults, helping reduce risks of diseases such as cancer, diabetes, cardiovascular disease and stroke, and delaying dementia onset [6]. Being consistently active for over six months has also been associated with reduced incidence of falls [7]. However older adults do little exercise and have difficulties maintaining exercise over time [6].

One technology of interest in supporting older people with meaningful activity, motivating exercise, and encouraging social connectivity is virtual reality (VR). There have been increasing numbers of studies exploring VR use within care settings [8]. Here we report a

For peer review only

study of a VR and omnidirectional treadmill system, ROVR Adventure (ROVR), designed for older adults [9]. The system is designed to motivate activity, physically navigating virtual environments that have meaning for the users. The use of environments with meaning to the user is essential to achieve the aim of meaningful activity, which, by definition, must be tailored to the users' preferences [10].

The VR environments in ROVR include places of cultural and historical interest such as ancient forts, museums, nature scenes and cityscapes. These 3D virtual environments represent places care home residents may no longer be able to visit physically. Access to culture (including heritage and history) is a human right [11], with documented health and wellbeing benefits, including improved quality of life, opportunity for reminiscence and connectedness to spaces and history [12]. Older adults in particular are invested in heritage, yet physical access to sites of cultural or heritage interest is often limited due to site characteristics and conservation issues (e.g., uneven ground, inability to implement accessibility features) [13]. The Heritage Alliance annual report [12] recommended partnership between heritage and health, to overcome access challenges to help realise the full potential of heritage for health and wellbeing. The Covid-19 pandemic highlighted the potential for online access to culture and heritage in place of physical access [14], with VR offering possible solutions [15]. The use of VR to visit cultural and heritage sites can therefore aid in inclusivity for those with physical and sensory barriers [15], particularly prevalent among older adults. The opportunity for reminiscence brings additional wellbeing potential, with reminiscence thought to provide stimulation, enjoyment, sense of self-worth and achievement [16]. It should also be considered however, that some concerns have been raised on use of VR with older adults [8]. For example, older adults can have vision-related challenges, and motion sickness in VR specifically for older adults should be thoroughly explored in research with end-users. Future research should consider how to achieve the intended exercise intensity and access to heritage and culture while ensuring resident comfort over suitable durations of VR use.

Alongside potentially motivating physical activity and providing access to cultural and heritage sites, ROVR allows for 'walks' to be socially interactive, with multiple users joining the same virtual environment to 'walk and talk' together, regardless of their location. The aim of the system therefore is to enhance social connectedness and improve physical health through meaningful activity [17]. However, prior to this study ROVR had never been used in care homes. With any technology, there may be significant differences in opinions between developers and end-users [18] so older people's feedback in design of technology is essential. Given potential safety concerns and the need to understand how to implement the technology we first needed to explore care home staff opinions on feasibility, design, and potential for older care home residents.

Method

Aims

The aims of this study were to explore staff perceptions towards acceptability and feasibility of the ROVR Adventure system for older adult care home residents, an understanding of

For peer review only - http://bmjopen.bmj.com/site/about/guidelines.xhtml

whether staff believe the technology will be feasible, acceptable or useful for care home residents is essential ahead of any direct feasibility study or implementation evaluation. Following study completion, results from the analysis were provided to the technology developer to help design iterations, based on staff feedback on potential improvements and design requirements.

Design

This study was a qualitative study based on technology interactions and focus group interviews, allowing staff to try, then to discuss, the equipment. Findings were presented to the technology developer allowing them to modify the design. Findings were presented verbally during meetings and in written form later, based on full analysis. We interviewed the Chief Executive about this and their experience of business working with research. This manuscript has been constructed following the COREQ checklist for qualitative research [19].

Location and dates

Data collection on perceptions of the ROVR standing treadmill and VR system took place from September to November 2021 in Looe, Liskeard, Saltash, St Austell, Perranporth, Camborne, and Truro in Cornwall UK. Feedback was provided to the developers at the time through verbal meetings and in written format via email. A follow-up interview with the technology developer took place via Zoom in September 2022.

Materials

Figure 1 shows the ROVR Adventure omnidirectional treadmill, with a Pico Neo 2 VR headset to immerse the users in the virtual environments. The headset is equipped with a 4K LCD display that provides a resolution of 1920 x 2160 pixels per eye with a field view of 101 degrees. The treadmill is a smooth plastic dish coated thinly with lubricant on which people stand wearing overshoes with ceramic plates on the base. This allows for a low friction, slippery surface on which people 'slide' their feet backwards and forwards to represent walking. The treadmill links to the ROVR PC software (in this study we ran the programme on a Lenovo ThinkPad L13) and VR system (Figure 1) via Bluetooth, which together, translates movement on the treadmill into movement through VR worlds. To change direction, participants swivel around and point their bodies in the direction of desired travel. VR worlds include 3D scanned, real world, heritage locations as well as animated worlds ranging from museums to simple houses (Figure 2). The ROVR VR worlds can also be social experiences, with several users entering the same world from distant locations and walking and talking together. Within the workshops, participants were first offered the opporunity to walk in the simple, animated house, to become accustomed to the experience before trying more complex models such as the heritage locations or museum. Each participant tried at least two or three of the VR environments. The participant was always 'joined' in the VR world by a researcher (as an avatar) linking in to the social world through a computer and walking with them via keyboard controls, to allow demonstration of the social angle.

(Figure 1 and 2 here)

For peer review only - http://bmjopen.bmj.com/site/about/guidelines.xhtml

Procedure

Convenience sampling was used. The health professionals were recruited at University of Plymouth e-Health events, through approach by the researcher (HB). Health professionals read participant information sheets and signed written consent forms before engaging in the workshop. The care homes were approached by email by HB, chosen based on prior collaboration with the researcher, to scope interest in trying the ROVR. For care home contacts who were interested, researcher visits were arranged, and participant information sheets and consent forms were provided. It is important to consider the impact of prior collaboration on results, although the participants may have had enhanced exposure to other technologies through prior work, they had not previously been involved in any VR studies. All participants provided written informed consent prior to taking part. Two researchers (HB, KE, both PhD holders with a background in digital health research and specific interest on social care) engaged in eight interaction workshops where a total of 44 key stakeholders (health and care staff) were provided with demonstrations of the ROVR Adventure and the opportunity to try the technology themselves. All staff tried the ROVR having at least 5 minutes of interaction, although some interactions were longer (>15 minutes), and all staff watched as other staff used the technology, including via casting of the VR world onto a computer screen, to help further inform their opinions through increased exposure to the technology in use. We used audio recording devices to capture comments during use. All 44 staff then participated in focus group discussions to share their perceptions. These were also audio recorded. Sessions lasted from 43-110 (mean 71) minutes. Sessions had between three and eight participants (Table 1). We were unable to link participants to distant ROVR users as a social experience due to feasibility but did demonstrate two users in one world (the researcher on laptop and participant on treadmill), to facilitate a discussion around the potential social experience. One of the workshops engaged health professionals such as occupational therapists and physiotherapists, and the other seven workshops engaged care home staff and managers.

For the one follow-up interview (approximately 10 months after the data collection and feedback), the technology developer was invited to take part in an informal interview to discuss their perceptions of the impact of the academic feedback from the focus groups on their products and processes. The interview was conducted by a separate researcher (AW) to those who had been collaborating with the technology developer for the purpose of this study, to avoid bias. The interview was one hour in duration.

Ethics

This study received ethical approval from the Faculty of Health Ethics Committee at the University of Plymouth, project ID 2887 (August 2021). All participants gave written informed consent.

Patient and public involvement

For peer review only - http://bmjopen.bmj.com/site/about/guidelines.xhtml

Considering the timing of this study, with health and care staff capacity still limited due to ongoing effects of the Covid-19 pandemic, it was not possible to involve them in the design, reporting or dissemination of this research.

Data Collection

Stakeholders provided feedback during their interaction with the ROVR Adventure treadmill and following the interaction during informal focus groups (interview schedule available in Appendix A). We used a semi-structure interview approach, following the schedule but deviating for natural conversation and follow-up questions on interesting comments. The feedback was audio-recorded and transcribed verbatim. The interview with the technology developer was also audio recorded and transcribed verbatim, there was no interview schedule used, but general queries posed to the developer on their perceptions towards the feedback from this study.

Data Analysis

Researchers (HB, LC, RB, ST, JR, AW, TK, SG, ARV) analysed the transcripts using thematic analysis. Braun et al. [20] detail the process of thematic analysis, a form of analysing qualitative data that is often used in health and social sciences. The process involves becoming familiar with the data set, generating initial codes, searching for themes, and reviewing and defining those themes. The sub-type of thematic analysis used was reflexive, in generating codes from explicit content, which evolves and adapts through considerable analytic work, to produce themes, representing an understanding of meaning across a dataset [20]. Analysis was inductive, as researchers had pre-determined aims. Analysis was conducted by nine researchers due to the size of the dataset, with a considerable amount of qualitative data collected. All nine researchers conducted initial coding, then HB, LC and RB led on iterations of reviewing and defining themes, however, the whole research team had two meetings to explore different perspectives on the data and codes to enhance validity of interpretation through perspectives from multi-disciplinary expertise (occupational therapy, psychology, physical activity science, computer science, social care research, digital design). We used the NVivo 12 data analysis software for data management to ensure a clear audit trail, enhancing dependability [21]. The involvement of a multidisciplinary team with various expertise in the data analysis processes enhances the credibility of our findings [22], the researchers who conducted analysis had diverse (yet, related to the topic) disciplinary backgrounds (including engineering, health, design), increasing robustness and significance of results (all researchers included have or are working towards a PhD). The process for the analysis of the follow-up interview with the technology developer was the same, although conducted by GR, AW and HB.

Participants

In total, across the eight interaction workshops, 44 participants interacted with the ROVR systems VR treadmill and provided evaluative feedback (Table 1). The participants included provided a broad range of appropriate stakeholders for English care homes, including across all levels of the operational care home staff and additionally various health professionals with expertise relevant for movement and activity (e.g. occupational therapist and

For peer review only - http://bmjopen.bmj.com/site/about/guidelines.xhtml

physiotherapist). For the follow-up interview with the technology developer there was one participant, the chief operating officer of the ROVR Systems technology developer.

Table 1: Participants at each interaction workshop

Site	Participants (n)
Workshop at University site 1	(5) General health and care professionals including occupational therapist, physiotherapist and patient involvement expert
Workshop at University site 2	(3) Supported living manager and care home staff
Workshop at Care home 1 – 26 bed residential care home	(3) Care home manager, compliance manager, activity coordinator and carer
Workshop at Care home 2 – 83 bed nursing home with unit for people with dementia	(7) Care home compliance manager, clinical needs manager, activity coordinator and carers
Workshop at Care home 3 – 47 bed care home for people with dementia	(6) Care home manager and carers
Workshop at Care home 4 – 33 bed care home	(8) Care home manager and carers
Workshop at Care home 5 – 40 bed care home	(7) Care home manager and carers
Workshop at Care home 6 – 60 bed nursing home for people with dementia	(5) Residential care nursing manager, carers

For peer review only

Results

Section 1: Workshop results

Here we discuss the results of the eight interactive workshops exploring stakeholder perceptions towards the VR motivated ROVR treadmill. In section 1, quotes have been identified by site where the data were collected rather than by individual participant. The roadshow/workshop nature of data collection and recording meant identifying individuals within busy transcripts was not feasible. In section 2, we share the results of the follow-up interview with the technology developer. Table 2 shows the themes and codes identified in the analysis of the eight workshops.

Table 2: The results of thematic analysis of the 8 interaction workshops. Evidence to support each code can be found in Appendix B and is synthesised in narrative below.

Theme	Initial Codes, *Sub-codes*
Anticipated benefits	Virtual visits; *Interesting local sites, Virtual tourist sites*<br>Physical activity/exercise; *Motivating exercise*<br>Prevents boredom<br>Rehabilitation<br>Staff use<br>Social interaction<br>Wellbeing

For peer review only - http://bmjopen.bmj.com/site/about/guidelines.xhtml

	Accessible experiences; *Different to care home*<br>Stimulates the brain<br>Restorative<br>Reminiscence; *Reminiscence benefits*<br>Expectations challenges positively<br>Positive distraction and novelty
Acceptability	Positive comments<br>ROVR is interesting<br>Adoption desired<br>Ease of use; *Headset initially heavy but comfortable, Physically easy, Treadmill is easy to enter, Treadmill is easy to move on, VR is novel experience*
Concerns of use	Secureness<br>Safety concerns; *Hitting objects, Shoes, Safety hazards, Danger*<br>Lack of control; *Movement issues*<br>Risk of falls/Dangerous; *History of falls, Could cause falls, Risk of injury - hips*<br>Accessibility<br>Prefer people go out<br>Heavy shoes<br>Lack of motivation<br>Uncomfortable movement<br>Bigger environment more physically demanding<br>Critique<br>Potential issues
Concerns of negative effects	Side effects; *Encouraging shuffling/sliding, Unnatural movement*<br>Dizziness<br>Disorientating; *Orienting to environment, Coordination between VR and feet, Height changes, Motion, Social angle confusing*<br>Concentrating more on the exercise<br>Depth perception difficulties<br>Motion sickness in VR<br>Fear
Suitability/Unsuitabilit y	Extra support; *Supervised use required, Guidance and guided navigation, Human resources, Number of residents, Differences between residents, Residents unable to use*<br>Diagnosis; *Balance, Dementia/cognition, Fear of falling, Have to be able, Incontinence, Influence of medication, Limited mobility, Problems with vision, Neck pain, Neuromuscular, Lived problem, Vertigo*<br>Suitability for residents; *99% not suitable, Suitable for residents 20 years younger, Wouldn't recommend to people with mobility issues*<br>Ethics<br>Risk<br>Size of technology; *WiFi connectivity*

For peer review only - http://bmjopen.bmj.com/site/about/guidelines.xhtml

	Familiarisation; *Adapting to use, Unfamiliar, Adjusting to headset, Familiarity*
Improvements	Content suggestions; *Cartoon-like images, New environments*<br>Inclusivity; *Adaptable bar height, Fitting to a wheelchair, People less mobile, Access could be improved, Physical requirements*<br>Safety improvements; *Softer impact bars, Stop slipping, Awfully slippery, Difficulty exiting treadmill*<br>Add arm movements<br>Cast visual for group to watch<br>Seated version; *Square, Headset use only, Adaptations*<br>Possibilities/exercise bike
Current design	Good content; *Visual Features, Spatial Features*<br>Liked headset<br>Safety features<br>Technology issues; *Glitching, Loading worlds, Speed of movement*<br>Ergonomics<br>Mechanics<br>Resolution

Anticipated Benefits

Participants noted a range of perceived benefits of using ROVR, with many focusing on the potential for physical benefits, such as providing "*good exercise*" (Care Home 2) and being "*particularly […] fantastic for rehab*" (Care Home 5). Participants noted that finding motivation to complete standard exercises can be a "*struggle*" and "*boring*" (Care Home 5), while the VR content would mean "*they can actually walk on that, something to motivate them to do it*" (Care home 5).

Participants also noted the potential enhanced accessibility of "*tourist sites*" (Care Home 5), enhancing visiting potential, particularly for those with "*reductions in mobility*" (Care Home 1). One staff member reported "*some [residents] are just not able to get out anymore […] you're providing the opportunity to see the place that they love*" (Care Home 4). This experience was additionally expected to create "*enhanced communication*" (Care Home 5) prompting conversations between residents with each other and the staff. Care staff additionally felt that reminiscence could be an additional benefit as it may "*trigger memories*" for the residents. (Care Home 2).

Acceptability

Further to the range of potential benefits noted by care staff, participants generally demonstrated good acceptability towards the device, often praising the technology as "*amazing, absolutely amazing*" (Care Home 3), "*honestly I think it's amazing*" (Care Home 6). One care home manager even reported that she would "*buy it for the staff and residents […] so that we actually had one permanently*" (Care Home 5).

Concerns of Use

For peer review only - http://bmjopen.bmj.com/site/about/guidelines.xhtml

Despite the general acceptability and praise towards the concept, and appreciation for potential benefits, participants noted some concerns about suitability of such devices for older adults and the care setting. Most concerns related to the safety of vulnerable older adults using a low-friction, stand-up treadmill. One care home manager reported that for care home residents specifically, the design was "*unsafe*" (Care Home 4), with another suggesting it was "*a danger*" due to being "*slippery*" (Care Home 5). The worry was that generally care home residents are "*not as agile as they used to be*" and could "*potentially fall*" (Care Home 3). This could result in injuries to "*ribs […] or your arm, break an arm*" (Care Home 5), with one carer suggesting "*I can see a few hips going*" (Care Home 2). As a result of these concerns, participants felt they would not want residents using the treadmill "*without a staff member*" (Workshop 2).

Concerns of Negative Effects

Beyond some safety concerns of residents using a stand-up, slippery treadmill, broader concerns were raised around "*motion sickness*" (Care Home 3), feeling "*dizzy*" (Care Home 5), and unnatural movements "*because you're not stepping*" (Care Home 5), "*it'll feel a bit weird*" (Care Home 6).

Suitability/Unsuitability

Participants debated the older adults in their care who ROVR would and would not be best suited to. Based on the concerns presented above, care home staff echoed that residents would "*need to have much more support*" to use the treadmill (Care Home 3), and to be "*supervised*" (Care Home 2), which would mean "*we're going to have to get the staff on board*" (Workshop 2). The treadmill was felt to be more suitable for residents with "*more stability when they're on their feet*" (Care Home 5), while care staff listed diagnoses that would make the standing treadmill less suitable. Suitability concerns were raised for people with "*lots of medication [that] affects their balance*" (Care Home 1), people with "*dementia*" (Care Home 4), who could "*totally freak*" (Care Home 6) and people with "*vertigo*" (Care Home 1). Despite the concerns, participants acknowledged that "*just like anything else, it's time to get used to it*" (Care Home 2), suggesting that prolonged use may encourage more confidence.

Improvements

Although participants were generally accepting and appreciative of the concept of VR to motivate physical activity, and acknowledged the potential benefits, the stakeholders were conscious of potential safety concerns which led to numerous participants suggesting a re-design of the treadmill to a "*seated unit*" which would "*be more beneficial to our client base*" (Care Home 2). Care staff suggested less mobile residents could be "*sat using their feet*" (Care Home 4) on a flat platform, to navigate the VR worlds without the risk of the standing, low-friction treadmill. Participants felt this would improve inclusivity of the experience as "*most [residents] come in in wheelchairs*" (Care Home 1). With these adaptations in mind, participants also shared thoughts on desired content for inclusion as a VR world, such as "*gardens*" (Care Home 4), "*under the sea*" (Care Home 5), or "*beaches*" (Care Home 4).

For peer review only - http://bmjopen.bmj.com/site/about/guidelines.xhtml

Current Design

While care stakeholders noted required adaptations to use VR to motivate movement and exercise safely for the target population of older adults and care home residents, participants praised aspects of the current design, including the VR content itself as "*amazing, brilliant, it is amazing, that is phenomenal*" (Care Home 5). They felt the headset "*doesn't feel too heavy on my head*" (Care Home 5), but "*feels really comfortable*" (Care Home 4). Participants did also note some technical glitches to be overcome, such as not "*going anywhere*" (Care Home 5) when walking, and "*flashing all over*" (Care Home 5). These forms of issue were reported to the developers for bug fixes during the study period.

Section 2: Results of the follow-up interview with the technology developer

Quotes have not been provided with an identifier as only one participant took part in this follow-up interview. Table 3 provides the main themes and initial codes and sub-codes resultant from analysis.

Table 3: The results of thematic analysis of the follow-up interview with the technology developer. Evidence to support each code is synthesised in narrative below.

Theme	Initial Codes, ***Sub-codes***
Benefits	Beneficial outcomes; P*hysical benefits, Social benefits, Mental benefits, Pushing personal boundaries, Benefits of Working with the University*
Process of development	Technology development; *Seated version*<br>Considering inclusivity; *Age, Care homes, Medical issues, Different sectors*<br>Phases of product development
Challenges	Cost and resources

Benefits

The follow-up interview with the technology developer highlighted a range of benefits born from the industry-academia collaboration. This included an initial code directly relating to *benefits of working with the University as* the participant reported it was "*really helpful*" through the opportunity to *"apply the minimum viable product [and figure] out where it works and where it doesn't"* stating *"you get really fast feedback."* The developer felt academic collaboration and real-world feedback from stakeholders provided *"feedback we can work with."* The participant stated: "*it's really important for commerce [industry] to understand this, they need to work with people who are already experts*" as the participant felt universities *"have the freedom to do what most companies can't."* In this case, the technology developer benefited from the extensive experience the researchers had in working within social care and with older adults, *"you were working in this environment and already had that knowledge and understood the challenges involved."* The developers felt bringing their specific technical knowledge and combining it with the university's evaluation expertise meant "*we didn't have to [...] learn it ourselves [...] or teach you [...], all we had to*

For peer review only - http://bmjopen.bmj.com/site/about/guidelines.xhtml

do was provide the equipment and gain feedback." The developer felt having evaluation through the University provided a "*helpful," "independent view" "which is unbiased."*

The technology developer also noted benefits they perceived of the technology itself, based on the interactions during the study period and subsequent collaborative studies (to be published elsewhere). The developer discussed how older adults in general can need "*motivation to exercise*" with one particular challenge "*in the Northern hemisphere*" being "*winters [which are] long and wet and dark*" leading to older adults becoming "*deconditioned over the winter."* Technologies such as ROVR provide an indoor solution to motivate physical activity, which could be achieved "*on a daily basis."* The developer also shared the benefits of the potential social connection facilitated within the ROVR VR worlds, "*they [older adult] visited [cultural location in VR] before, they'd love to visit. They can meet up with friends, they don't have to be in the same location, they can join, without necessarily having VR equipment yeah. And that makes this a social interaction."* Beyond the physical activity and potential for social contact, the developer felt they observed that the technology *"cognitively de-stressed*" older adults, meaning a range of potential benefits were observed.

Process of development

During the interview, the technology developer detailed how feedback from the current study helped lead to development of a seated version of the ROVR treadmill (ROVR Explore), which they felt was particularly required for "*the level of condition we found in care homes*" as "*they're really seated […] without major assistance for a long time*" meaning they couldn't use the standing ROVR. The participant stated: "*one of the limitations, […] is the inability to move around safely*." The developer responded with "*a plate, which could be sat in front of people if they were seated and enable them to move."* The participant stated: "*it was a real opportunity to include people who would otherwise be excluded*" demonstrating significant value in this insight and product development as a result of the collaboration.

Challenges

The technology developer also shared some thoughts on challenges they face generally, such as cost. Before 2012, VR development was only for those *"with a million pound budget*," and though costs have fallen dramatically the feeling is that "*costs of development are still pretty high at present*" with "*various challenges around the world adding to those costs"*. Development costs and associated challenges further highlight the value of evaluation, iterative design and co-development with stakeholders, ensuring early developments are suitable and desirable for the intended audience before funds are wasted on inappropriate mass production.

Discussion

This study explored health and care staff perceptions towards the potential for VR motivated treadmill exercise for older adult care home residents, which is important due to the impact of the ageing population and growing burden of disease [2]. Research into the

For peer review only - http://bmjopen.bmj.com/site/about/guidelines.xhtml

potential for technological innovations to support healthy ageing is essential to help respond to these challenges [3]. Our study adds to the literature on design of technology to encourage and support physical activity and exercise in older adults and care home residents.

Novel technologies such as the ROVR have potential to support healthy ageing. Our results demonstrate clear promise for such technologies to motivate and sustain increased physical activity in older adults and people living in care homes, with our large sample of care staff generally agreeing on potential benefits for exercise and activity. Implementation of products such as these would likely have benefits for meaningful activity, meeting the NICE [10] definition of providing an activity that promotes health and wellbeing through an activity with meaning to the individual, and is tailored to their preferences. Our results have demonstrated an interest and preference among stakeholders for cultural, heritage and natural sites for use as a meaningful VR experience. Further work is now required with older adults themselves to confirm their preference for such an activity.

Staff also perceived benefits for social interaction, through both the social aspect of the VR platform but additionally through increases in residents talking to each other, their families and carers with the technology experience providing a focal point. This could have implications for the experience of loneliness among the target population [5, 23]. Our results suggest the technology was also expected to allow for reminiscence and enhanced experiences, through allowing older adults and care home residents to 'visit' places and heritage sites they could no longer access. Staff also noted that older adults may be able to 'visit' places significant to them through memories from the past. The benefits of access to heritage locations are well documented [12], therefore future research could explore and document the impact virtual access that is paired with technology that involves exercise to move around heritage sites has on health and wellbeing outcomes. Further exploration is also required to quantitatively assess the impact of the physical activity (or motivation to exercise) over longitudinal periods on outcomes for older adults. Additional future research should also seek to document the technical competence required for care staff to implement and facilitate such technologies without researchers present.

However, despite the positive perceptions towards the concept of VR to motivate activity and use of some form of treadmill to encourage movement and muscle use, care staff raised concerns around product design, mostly related to safety. Participants were concerned that for the frailer older adults and care home residents, standing on the low-friction treadmill could present a falls risk. When results were provided to the technology developer, this study had immediate implications for product design. Based on this stakeholder feedback, the ROVR Systems team subsequently developed the Explore system, a seated version of the omnidirectional treadmill which would not present a falls risk while still motivating activity and lower leg movement to build up leg strength. The seated Explore system is currently undergoing evaluation with stakeholders as part of the ongoing GOALD project [24]. Beyond this immediate effect, this study has broader implications for those designing physical activity technologies aimed at supporting aged care. It demonstrates that care stakeholders are open and accepting to such technologies, believing the concept to be

For peer review only - http://bmjopen.bmj.com/site/about/guidelines.xhtml

positive, while also providing useful design, hardware, and content insight for future developments. This value is clearly demonstrated in this study through the follow-up interview with the developer, and subsequent new product development.

This study also reinforces previous work [1, 18], showing the importance of engaging stakeholders in the design and testing of technologies aimed at health and care. The follow-up technology developer interview showed the value of academic-industry collaboration. The developers were able to use these findings to adapt the technology into a seated treadmill, resulting in an enhanced safety experience for those unable to comfortably stand and to increase the accessibility of the experience and its potential health and wellbeing benefits.

Strengths and limitations

Our study achieved a relatively large sample for qualitative work and gained deep and thorough insight into care staff perceptions. Our analysis used rigorous methods and the topic of enquiry was novel, in exploring VR motivated physical activity specifically for older adult care home residents.

The strong qualitative approach produced data that was substantial and complex, and although the themes and categories presented here encompass all data, there is additional evidence of more profound and complex forms of engagement with VR technologies, such as speculative feedback or feelings of telepresence. These additional dimensions warrant further investigation.

There were two limitations. First, the duration for which participants interacted with ROVR was short. Our findings therefore may be over optimistic and influenced by the novelty effect. Longitudinal work would have better helped identify barriers and facilitators to use that would not be immediately recognised. The concerns of negative effects reported in our results, such as dizziness and motion sickness, would likely have been better assessed in longitudinal work, allowing time for participants to familiarise and overcome any initial negative sensations. These types of reactions are common among all VR systems, with work underway to generally reduce cybersickness experiences for users of head mounted displays [25]. Research has shown that repeated exposure to the same VR content will reduce severity of motion sickness [25], thus a longitudinal or repeated measures study would more accurately report on the degree of barrier presented by the unpleasant sensations. As with the potential reduction in motion sickness over time, participants themselves noted that it would take time to gain confidence in using the ROVR device with older adults and care home residents, therefore demonstrating potential to be more accepting over time.

Secondly, we only sought staff opinions in the potential future use of these devices, rather than older adults themselves. However, for devices such as these to be used by older adult care home residents, as intended by the developers, they first need to be accepted and adopted by the staff team who would be responsible for buying and facilitating use. To overcome both the limitation of short interaction period and staff stakeholders only, we have conducted a 6-week implementation study with the ROVR device to be reported separately. It should also be considered that we did not explore device cost or site

For peer review only - http://bmjopen.bmj.com/site/about/guidelines.xhtml

investment potential, therefore for real world implementations, cost would be a consideration.

Conclusion

The use of VR motivated exercise on an omnidirectional treadmill holds potential based on the perceptions of care staff for increasing exercise of older care home residents, while also providing virtual access to heritage sites, encouraging reminiscence and social connectivity, and promoting participation in a meaningful activity. A standing device however was not appropriate for a care home population and a new design is now being used for research with care home residents. User-centred design of health and care technologies is essential, and this study has demonstrated significant benefits for industry partners in collaborating with university institutions for independent evaluation with end-users.

Funding

This work was supported by UKRI Healthy Ageing Social, Behavioural and Design Research Programme grant number ES/V016113/1 as the project was completed by researchers on the GOALD project and the project also purchased ROVR equipment for the study, with further support from the EPIC (eHealth Productivity and Innovation in Cornwall and the Isle of Scilly) project which was part funded by the European Regional Development Fund. Additional funding for the EPIC project was received from University of Plymouth. ERDF Grant number [05R18P02814], as EPIC also purchased some of the ROVR equipment used. Finally, support was also provided by Innovate grant 10004423, which funded development, equipment and support provided by ROVR Systems Ltd.

Competing interests

The authors declare no competing interests.

Author Contributions

HB and KE led on the design of the research. HB, KE, LC, AW and RB conducted data collection. HB, LC, RB, AW, TK, ST, JR, GR, SG and AVR conducted data analysis. RJ oversaw the project design, completion and substantively contributed towards the write up, which was led by HB. SA, KW, CH, ACW, AC and RH oversaw study design and completion as GOALD senior management with RJ and reviewed and contributed towards the manuscript. All authors reviewed, edited and agreed to the final manuscript.

This project has received funding from the UKRI and ESRC Healthy Ageing Social Behavioural and Design Research Programme (SBDRP) grant No ES/V016113/1.
https://www.plymouth.ac.uk/research/centre-for-health-technology/goald

For peer review only

For peer review only - http://bmjopen.bmj.com/site/about/guidelines.xhtml

Generating Older Active Lives Digitally

Healthy Ageing Challenge
Social Behavioural Design
Research Programme

UK Research
and Innovation

on behalf of the GOALD project: Catherine H. Hennessy, Faculty of Social Sciences, University of Stirling, Ray B. Jones, Faculty of Health, University of Plymouth, Richard Haynes, Faculty of Arts & Humanities, University of Stirling, Anna C. Whittaker, Faculty of Health Sciences & Sport, University of Stirling, (Core Management Team) and Sheena Asthana, Plymouth Institute for Health and Care Research (PIHR), University of Plymouth, Rory Baxter, Faculty of Health, University of Plymouth, Hannah Bradwell, Faculty of Health, University of Plymouth, Arunangsu Chatterjee, Faculty of Medicine and Health, University of Leeds, Pete Coffee, School of Social Sciences, Heriot-Watt University, Leonie Cooper, Faculty of Health, University of Plymouth, Alison Dawson, Faculty of Social Sciences, University of Stirling, Katie Edwards, Faculty of Health, University of Plymouth, Swen Gaudl, Faculty of Science & Engineering, University of Plymouth, Tanja Krizaj, Faculty of Health, University of Plymouth, Gregory Mannion, Faculty of Social Sciences, University of Stirling, Gemma Ryde, Institute of Cardiovascular and Medical Sciences, University of Glasgow, Alejandro Veliz Reyes, Faculty of Arts, Humanities and Business, University of Plymouth, John Ritchie, Faculty of Arts & Humanities, University of Stirling, Simone Tomaz, Faculty of Health Sciences & Sport, University of Stirling, Alison Warren, Faculty of Health, University of Plymouth, Karen Watchman, Faculty of Health Sciences & Sport, University of Stirling, Katherine Willis, Faculty of Arts, Humanities and Business, University of Plymouth, and partners - Active Stirling Ltd., Generations Working Together, Hearing Loss Cornwall, Nudge Community Builders, South Asian Society (Devon and Cornwall), Sporting Heritage, Sports Heritage Scotland, St. Breward Community, iSightCornwall, UKActive.

Acknowledgements

We would also like to thank our collaborating care homes for hosting our workshops and colleagues on the EPIC project who helped establish the collaboration.

Data sharing

Anonymised data is available from the corresponding author at reasonable request.

References

For peer review only - http://bmjopen.bmj.com/site/about/guidelines.xhtml

1. Moyle, W., Jones, C., Pu, L., & Chen, S.-C. (2018). Applying user-centred research design and evidence to develop and guide the use of technologies, including robots, in aged care. Contemporary nurse, 54(1), 1-3. doi:10.1080/10376178.2017.1438057

2. Abdi, J., Al-Hindawi, A., Ng, T. & Vizcaychipi, M.P. (2018). Scoping review on the use of socially assistive robot technology in elderly care. BMJ Open, 8(2), e018815.

3. UK Parliament (2018). Robotics in Social Care. Houses of Parliament, Post Note. [Internet] Available from: https://post.parliament.uk/research-briefings/post-pn-0591/

4. Garçon, L., Khasnabis, C., Walker, L., Nakatani, Y., Lapitan, J., Borg, J., et al. (2016). Medical and Assistive Health Technology: Meeting the Needs of Ageing Populations. Gerontologist, 56, Suppl 2:S293-302.

5. Siniscarco, M. T., Love-Williams, C., & Burnett-Wolle, S. (2017). Video Conferencing: An Intervention for Emotional Loneliness in Long-Term Care. Activities, Adaptation & Ageing, 41(4), 316-329. doi:10.1080/01924788.2017.1326763

6. Langhammer B, Bergland A, Rydwik E. The Importance of Physical Activity Exercise among Older People. Biomed Res Int. 2018 Dec 5;2018:7856823. doi: 10.1155/2018/7856823. PMID: 30627571; PMCID: PMC6304477.

7. Sherrington, C., Michaleff, Z. A., Fairhall, N., Paul, S. S., Tiedemann, A., Whitney, J., ... & Lord, S. R. (2017). Exercise to prevent falls in older adults: an updated systematic review and meta-analysis. British journal of sports medicine, 51(24), 1750-1758. http://dx.doi.org/10.1136/bjsports-2016-096547

8. To-Miles F, Mann J, Hung L (2022). Facilitators and barriers to using virtual reality and augmented reality and its impact on social engagement in aged care settings: a scoping review protocol. BMJ Open, 12, e061722. doi: 10.1136/bmjopen-2022-061722

9. ROVR Wizdish (2021). Step into a new reality: ROVR systems. [Online] [Accessed 4/11/2021] Available from: https://rovr.systems/

10. NICE (2013). Mental wellbeing of older people in care homes. Quality standard QS50. Available from: https://www.nice.org.uk/guidance/qs50/chapter/quality-statement-1-participation-in-meaningful-activity#:~:text=Meaningful%20activity%20includes%20physical%2C%20social,crafts%2C%20conversation%2C%20and%20singing. [accessed 13/02/23].

11. UNESCO (2021). Right to participate in cultural life. UNESCO. [Online] [Accessed 2/11/2021]. Available from: https://en.unesco.org/human-rights/cultural-life

12. Heritage Alliance (2020). Heritage Alliance Annual Report. [Online] Available from: https://www.theheritagealliance.org.uk/wp-content/uploads/2020/10/Heritage-Alliance-AnnualReport_2020_Online.pdf

13. Marasco, Alessandra & Balbi, Barbara. (2019). Designing accessible experiences for heritage visitors through virtual reality. e-Review of Tourism Research. 17.

For peer review only

For peer review only - http://bmjopen.bmj.com/site/about/guidelines.xhtml

14. Kużelewska, E., Tomaszuk, M. European Human Rights Dimension of the Online Access to Cultural Heritage in Times of the COVID-19 Outbreak. *Int J Semiot Law* (2020). https://doi.org/10.1007/s11196-020-09712-x

15. Paladini, A., Dhanda, A., Ortiz, M. R., Weigert, A., Nofal, E., Min, A., … Quintero, M. S. IMPACT OF VIRTUAL REALITY EXPERIENCE ON ACCESSIBILITY OF CULTURAL HERITAGE. ISPRS - International Archives of the Photogrammetry, Remote Sensing and Spatial Information Sciences, XLII-2/W11, 929–936. https://doi.org/10.5194/ISPRS-ARCHIVES-XLII-2-W11-929-2019

16. Dempsey, L., Murphy, K., Cooney, A., Casey, D., O'Shea, E., Declan, D., Jordan, F. and Hunter, A. (2014), 'Reminiscence in dementia: a concept analysis', Dementia, 13 (2), pp. 176-92, doi: 10.1177/1471301212456277

17. Smith, N., Towers, A-M., Plamer, S., Beecham J. & Welch, E. (2018). Being occupied: supporting 'meaningful activity' in care homes for older people in England. Cambridge University Press.

18. Bradwell, H.L., Edwards, K.J., Winnington, R., Thill, S. & Jones, R.B. (2019). Companion robots for older people: importance of user-centred design demonstrated through observations and focus groups comparing preferences of older people and roboticists in South West England. BMJ Open, 9(9), e032468.

19. Tong A, Sainsbury P, Craig J. (2007). Consolidated criteria for reporting qualitative research (COREQ): a 32-item checklist for interviews and focus groups, International Journal for Quality in Health Care, 19(6), 349–357. doi.org/10.1093/intqhc/mzm042

20. Braun, V., Clarke, V., Hayfield, N. & Terry, G. (2019). Thematic analysis. In Liamputtong, P. (Ed.). Handbook of Research Methods in Health Social Sciences. https://doi.org/10.1007/978-981-10-5251-4_103

21. Bonello, M., Meehan, B. Transparency and coherence in a doctoral study case analysis: reflecting on the use of NVivo within a 'Framework' approach. The Qualitative Report. 2019;24(3):483-98

22. Morse, J.M., Field, P.A. Qualitative research methods for health professionals. 1995.

23. Campaign to End Loneliness (n.d.). The facts on loneliness. [Online] Available from: https://www.campaigntoendloneliness.org/the-facts-on-loneliness/

24. University of Plymouth (2022) Generating Older Active Lives Digitally. [Online] Available from: https://www.plymouth.ac.uk/research/centre-for-health-technology/goald [accessed 01/03/23].

25. Chang, E., Kim. H.T. & Yoo, B. (2020) Virtual Reality Sickness: A Review of Causes and Measurements, International Journal of Human–Computer Interaction, 36:17, 1658-1682, DOI: 10.1080/10447318.2020.1778351

Figure legends

For peer review only - http://bmjopen.bmj.com/site/about/guidelines.xhtml

Figure 1: (Left) Care home staff member interacting with the ROVR Adventure treadmill with researcher recording initial reactions. (Right) VR headset with the ROVR Adventure omnidirectional treadmill and example of the app to access VR worlds

Figure 2: Example of the virtual museum participants could explore by walking on the ROVR Adventure

For peer review only

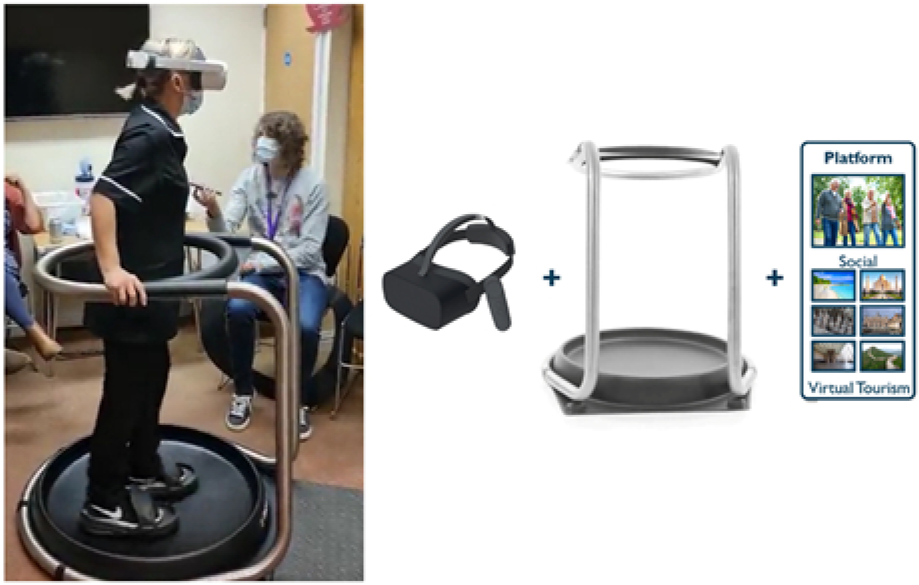

Figure 1: (Left) Care home staff member interacting with the ROVR Adventure treadmill with researcher recording initial reactions. (Right) VR headset with the ROVR Adventure omnidirectional treadmill and example of the app to access VR worlds

124x79mm (96 x 96 DPI)

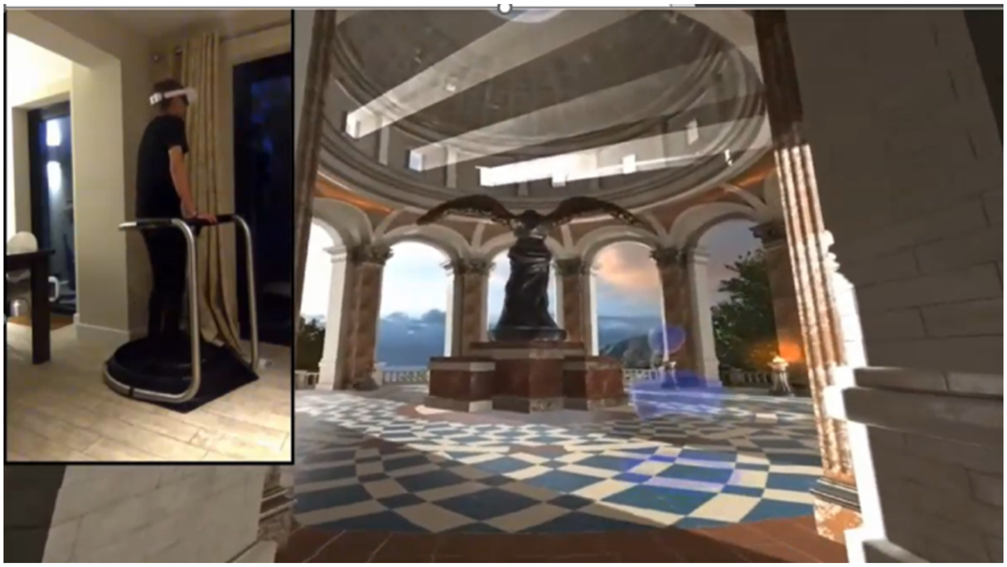

Figure 2: Example of the virtual museum participants could explore by walking on the ROVR Adventure

248x138mm (96 x 96 DPI)

Appendices

Appendix A –Workshop Interview Schedule

Site information

1) Please briefly describe the site you work at? (e.g. care home, extra care, supported living)

2) Please describe the number of residents or customers at that site and the level of care required? (e.g. physical ability of residents, prevalence of dementia)

Feedback on ROVR

1) What are your overall thoughts on the ROVR? How physically demanding did you find use of the ROVR?

2) What, if any, benefits do you perceive older adults might gain from using the ROVR?

3) What, if any, challenges might be encountered in the use of the standing or seated ROVR system with older adults (and care home residents)?

4) What characteristics do you think older people would need to use the standing and/or seated ROVR safely?

5) What challenges or health conditions do you think might make use of the standing and/or seated ROVR unsafe? (e.g. visual impairments, osteoarthritis, balance and coordination issues, frailty, mobility issues, support with grooming/communication/transfers/toileting/locomotion/self-care/communication *examples taken from the Functional Independence Measure*)

6) If the ROVR was in your care facility, do you envisage that it would change ways of working? (if so, how?)

7) Based on the video and demonstration you saw today, would you feel confident supporting use of the ROVR at your care facility? (further ideas on training requirements)

For peer review only

Appendix B – Supporting evidence for Table 2

Theme	Initial Codes, *Sub-codes*	Example Evidence
Anticipated benefits	Virtual visits	
	- *Interesting local sites*	"Eden project, Tintagel, Cornish mines, that kind of thing. That's the hope, and particularly post COVID with kind of reductions in mobility." (Care Home 1)
	- *Virtual tourist sites*	"Some of these tourist sites to visit. So you could go to Venice or Paris." (Workshop 1)
	Physical activity/exercise	"And it does exercise the top of the body as well. Yeah, obviously you need to push down. Yeah, take the weight off your feet when you slide. So it's, well, it is a whole body exercise really" (Care Home 5)<br>"You know, it's making each day have something new or useful or different, and this could open that to them. A massive, massive but as well as you know, without them realizing bit by bit and increasing their fitness, mobility, their longevity, their life span, their health" (Workshop 2)
	- *Motivating activity*	"And you know, struggle to get [exercise], how boring it is. If they can actually walk on that something to motivate them to do it. Yes, massive motivation isn't it. " (Care Home 5)
	Prevents boredom	"Oh, it's good exercise as well as Yeah, it's actually giving them an experience. It's getting a mobile, perhaps someone that might just sit in their chair normally." (Care Home 2)<br>"And [a resident] gets bored sometimes in the afternoon to the evening. And he's getting his coat or going out for a walk. Well, if we set him up on there. You could have a walk on here instead" (Care Home 5)
	Rehabilitation	"We had a bloke the other day that had a stroke. In the April he was discharged from hospital they couldn't do any more for him and he wasn't standing, wasn't walking. He went home from us because we, our staff were inputting exercise […] There was another lady […] But she was off her legs. And we've got her going and then she went home again. Yeah. If we can get close enough [to the ROVR] with the chair that they can actually sit and do it, it would be [rehabilitating]." (Care Home 5)<br>"Particularly I think it'd be fantastic for rehab. Absolutely. Brilliant. People go that have to walk up and down bars." (Care Home 5)
	Staff use	"Yeah. even if our staff will be using it, our staff in front of our residents, makes good health and wellbeing staff and a morale boost. Yeah, because it's something new, something exciting that they can all talk about. So it's not just not just yeah, not just the service users, the staff as well as the whole well being." (Care Home 5)
	Social interaction	"The novelty factor and the benefits of something different to what they've experienced in it, the talking points for themselves and their families that they probably can hang on to for quite a while." (Workshop 1)<br>"I'm anticipating, essentially, social interaction, with the carers a bit more because there's going to be more communication? Yeah. So enhanced communication. Yes. Exercise. Yes. Yeah. Exercise. And interesting life." (Care Home 5)<br>"[They can talk to each other] Yeah. I think that's the best part of it" (Care Home 2)<br>"So maybe in time, it would be nice to involve the communities in and around our houses." (Workshop 2)
	Wellbeing	"Ultimately, if they're using it, their well being is completely going to change and they're going to be happier." (Care Home 1)<br>"But I think for mental health it would be good" (Care Home 2)
	Accessible experiences	"So some are just not able to get out anymore. Yeah. So if you're providing the opportunity to see the place that they love" (Care Home 4)
	- *Different to care home*	"If you're living in a care home, to go to the different, that environment was lovely." (Workshop 1)
	Stimulates the brain	"Stimulates the brain. Yeah. Got me thinking." (Care Home 1)
	Restorative	"that will bring their confidence back" (Workshop 2)
	Reminiscence	"Especially if they're walking in an area they know. They are walking down. Trigger memories" (Care Home 2)

For peer review only - http://bmjopen.bmj.com/site/about/guidelines.xhtml

	- *Reminiscence benefits*	"Well, I think it's memories, it's reminiscing for them, isn't it? If they went to it's like, you know, bearing in mind not everybody that lives here is from Devon or Cornwall, we've got people that have moved from the land at some point. So even to have those places it brings back memories. You know, we've got old photographs of Saltash, pictures of Saltash, and that, you know, they'll say, Oh, we went to that cinema when I was young. You know, it brings back memories. So I'm presuming this will do the same. I'll show them because yeah, A lot of living with dementia. They're living years ago." (Care Home 4)
	Expectations challenges positively	"That was alright actually, I didn't think, it wasn't as bad as I thought it was going to be. I felt more in control than I thought I would." (Care Home 2)
	Positive distraction and novelty	"What we were saying, and you know you said how was things I think, you know, people at the moment are feeling so overwhelmed. Yeah, we've been through something like this would take their minds off things" (Care Home 2)
Acceptability	Positive comments	"Yeah, that's amazing. Absolutely amazing" (Care Home 3)
		"I honestly think this is amazing" (Care Home 6)
	ROVR is interesting	"Interesting idea." (Workshop 1)
	Adoption desired	"I would […] buy it for the staff and residents. I would consider that, I would put it before my board so that we actually had one permanently." (Care Home 5).
	Ease of use	"Easy to control" (Care Home 5)
	- *Headset initially heavy but comfortable*	"It feels a little bit heavy forward so I've got to bring it forward, but it is good." (Workshop 1)
	- *Physically easy*	"Not for me intense at all." (Workshop 1)
	- *Treadmill is easy to enter*	"In felt fine [getting in and out], because at least I had one stable foot on the ground, and sort of the bars were in front of me." (Workshop 1)
	- *Treadmill is easy to move on*	"I actually find the walking on it much easier than I thought it would be to be." (Workshop 1)
	- *VR is novel experience*	"One [benefit], the novelty factor and the benefits of something different to what they've experienced in it." (Workshop 1)
Concerns of use	Secureness	"That does feel more secure, leaning on the bars around you. And it helps to keep you stable." (Care Home 3)
	Safety concerns	"Yeah, just whilst they still think they can walk. And while that seems pretty easy, yeah, that potentially is gonna make them really unstable. Or they'll get breathless really quick" (Care Home 3)
		"It's unsafe I would say as strong as that they would be unsafe" (Care Home 4)
	- *Hitting objects*	"... Oh, I've got a move in a minute, because I'm going to hit, say the bollard, I already hit it. And I wanted to go up the stairs. But I couldn't go upstairs because I'd hit it already..." (Care Home 1)
	- *Shoes*	"The shoes are going to be a little bit of an issue. If they can come in different sizes that might be But the smaller they are, the tighter they're going to be. So I found that quite tight to fit the one large and I've only got size three feet. Yeah. very slippery to stand on. And I can see that being quite an issue" (Care Home 1)
	- *Safety hazards*	"Maybe you need something around the outside. So if they fell, they just they can't come out. They can only go on their knees then can't they?. Do you know what I mean? If there was a guard? Actually, because that's my worry if someone fell out of that." (Care Home 5)
		"You see that wouldn't be suitable for a resident. That is too slippery" (Care Home 4)
		"That's that's a slippery slope." (Workshop 2)
	- *Danger*	"They [residents] can't afford to fall, if they fall it's gonna be an injury isn't it" (Care Home 6)
		"It's a bit slippery to get on and off? That is a danger" (Care Home 5)
	Lack of control	"It's it's it's fast and then it's because you can't control it" (Care Home 3)

For peer review only

	- *Movement issues*	"I'm not really moving anywhere." (Workshop 1)
	Risk of falls/Dangerous	"more support for them because the type of people that we get like I've said are not as agile as they used to be even though sometimes they think they are and they're potentially going to fall in that." (Care Home 3)
	- *History of falls*	"I don't know that she'd be happy to do it. Because she's got a history of falls. Okay, so she'd probably not enjoy it." (Workshop 1)
	- *Could cause falls*	"I think when it could actually make someone fall." (Workshop 1)
	- *Risk of injury – hips*	"If you slipped now, you're gonna bang yourself on those bars, then you're gonna catch your ribs or whatever? Or your arm, break an arm on there if you go down, yeah." (Care Home 5)<br>"Yep. Okay. I can see a few hips going." (Care Home 2)
	Accessibility	"Getting in was alright, getting out was difficult, because you've already done that slippery surface" (Care Home 3)<br>"You could do with like a virtual reality wheelchair, they sit down and go, around." (Care Home 5)
	Prefer people go out	"the deterioration that we've seen in so many of our residents is tenfold and I'd rather get them out. But then you have that knock on effect that the families don't want to. But if they have the option of liaising via a website, let's go and visit Eden with her" (Care Home 1)
	Heavy shoes	"Yeah, the heaviness of those shoes, and being nervous of falling." (Workshop 1)
	Lack of motivation	"Definitely. Yeah. There's lots of 'Mister, come on, you can do it. You can do it.', 'I don't want to do it'." (Workshop 1)
	Uncomfortable movement	"I feel like the twisting itself, which is where the uncomfortable sensation perhaps maybe lies." (Workshop 1)
	Bigger environment more physically demanding	"Depends on how big the world is, because this world feels small and so I feel like I have to twist." (Workshop 1)
	Critique	"I wouldn't want them using it without a staff member on the site." (Workshop 2)<br>"Resident would be screaming the place down, and panic about the step [into ROVR]" (Care Home 6)
	Potential issues	"this idea of being in an open space or field might be disorientating" (Workshop 2)
Concerns of negative effects	Side effects	"Yeah. It wasn't motion sickness but I just started to feel like I just need to take this off now, yeah, it's just a really strange feeling" (Care Home 3)<br>"Yeah, I've got a feeling of like, yeah, maybe I've got like a bit of car sickness." (Care Home 4)
	- *Encouraging shuffling/sliding*	"That is almost the encouraging of Parkinson's type shuffling isn't it?" (Workshop 1)
	- *Unnatural movement*	"As you move around this change of texture to this and that makes it seem like it's not as organic or not as natural" (Workshop 1)<br>"A bit weird, because you're not stepping" (Care Home 5)<br>"Keep sliding […] it'll feel a bit weird" (Care Home 6)
	Dizziness	"I might feel dizzy. It was alright there but when you go off the steps and stuff you start getting a bit. It's because when you went the faster you go. Then when you turn that's when you get a bit. Isn't it like discombobulated?" (Care Home 5)<br>"It feels really weird, my head is still spinning" (Care Home 6)
	Disorientating	"That's amazing but it messes with your head" (Care Home 3)<br>"Trying to orientate yourself is quite difficult. Yeah. Yeah. Especially around the house." (Care Home 1)
	- *Orientating to environment*	"Especially when you got the headset on, you're disorientated as well." (Care Home 4)<br>"The only thing that concerns me is the disorientation that. Yeah, that's the only thing. Okay, Whether they'd be disorientated." (Care Home 2)<br>"Yeah, I think it'd be really hard because you lose all your visual cues like even though I've got no balance problems it was pretty tricky. You do certainly do lose your orientation before you put those on, like, I didn't know where anyone was. Deal with the disorientation and the

For peer review only

		nausea. Anyway, which they might not be able to tell you about, so I'd be worried about it for dementia. Without a lot of, it's worth trying." (Workshop 1)
	- *Coordination between VR and feet*	"Yeah, since you've had the headset on your feet don't seem in momentum. Is it difficult to watch and organise your feet?" (Care Home 4)
	- *Height changes*	"It [*disorientation*] didn't affect me till there was like the change in height" (Workshop 1)
	- *Motion*	"Motion is kind of backwards and forwards, like you're on a cross trainer." (Workshop 1)
	- *Social angle confusing*	"For a few? Most of them I would say it [social experience in VR] would be very confusing." (Workshop 1)
	Concentrating more on the exercise	"Concentrating more on the exercise." (Workshop 1)
	Depth perception difficulties	"Because it is such a such a strange sensation of a lot of different coordination at the same time, and that the depth perception issues" (Workshop 1)
	Motion sickness in VR	"Oh, I'm actually feeling really dizzy." (Workshop 1)<br>"I felt motion sickness when I got off yeah. But I think it was too enclosed. Like, there's too many walls and things. I hit a wall quite quickly," (Care Home 4)
	Fear	"I think some elderly people would find it quite scary. Yeah, I do." (Care Home 1)<br>"They [residents] might feel a bit scared, because because it's slippy." (Care Home 2)
Suitability/Unsuitability	Extra support	"I think the worry for me is is if we get to the stage where the residents go in there. Yeah, I think we need to have much more support for them" (Care Home 3)
	- *Supervised use required*	"I don't think I'd feel happy start with if they weren't being supervised." (Care Home 2)<br>"Yeah, but you still need to hold on it. But I think what you would always do with the residents on that, or you would always have one person here" (Care Home 5)
	- *Guidance and guided navigation*	"I was just saying, if your staff can't see what's going on, when you run into problems, they can't troubleshoot it for someone who's struggling cognitively, or even just unfamiliar with it, like, am I on the balcony? Do I need to get to it? If I'm not on the balcony, how do you guide someone up to it? What do I do? They can't then put the mask on, with infection, you can just put the mask on and check, you've got to take someone's headset off." (Workshop 1)
	- *Human resources*	"I do think it will need some supervision, so definitely, we're going to have to get the staff on board with it. Because well, I, it's not something that I would expect to walk in at nine o'clock in the morning and see someone on it without one of us being there" (Workshop 2)
	- Number of residents	"they all they've all got walking frames and need wheelchairs. And even that's residential. I would say only a couple." (Care Home 1)<br>"I can think of one called [resident name]. Yeah, that's about the only one I will allow would feel comfortable with." (Care Home 4)<br>"Obviously you just have to pick the residents that would be appropriate for, because someone with mental health issues would really struggle on this." (Care Home 4)
	- *Differences between residents*	"[Suitable for residents with] More stability when they're on their feet" (Care Home 5)
	- *Residents unable to use*	"I don't know if a resident would be able to do that" (Care Home 1)
	Diagnosis	"Imagine that somebody with cognitive impairment or a spatial awareness issue, poor eyesight, mobility wise, […] not somebody of age (Care Home 6)

For peer review only - http://bmjopen.bmj.com/site/about/guidelines.xhtml

	- *Balance*	"I think it's the overall, you know, they are on lots of medication, how that's going to affect their balance. I've got some people who have vertigo, they wouldn't be able to do it." (Care Home 1)
	- *Dementia/cognition*	"First, immediately, people are just gonna let go. Okay, especially if they got mild dementia and something like that" (Care Home 1)<br>"And of course they are in a not in a reality situation. So they don't know actually where they are." (Care Home 5)<br>"Has anyone said about the issue about with the mental health. They might think why are here? Yeah. Why are we keeping them from them. There's some here that would love to be in Australia with the granddaughter and they would fully understand that it's a Virtual world and also coming back out and leaving that experience then suddenly the grandchild is gone again. Someone living with dementia only got to see somebody crossing the car park, and they think it's one of their family and they think why do you not let them in, it's not their family but they think." (Care Home 4)<br>"I feel like it would totally freak our clients [...] I think better cognition" (Care Home 6)
	- *Fear of falling*	*Interviewer:* "Yeah. Do you think that that slipperiness would be a problem? "<br>*Participant:* "It would be for some of our residents here. Yeah. Yeah. They would just fall over" (Care Home 1)<br>"Just what we've already said, totally inappropriate for residents because if they fall." (Care Home 4)
	- *Have to be able*	"They've got to be quite able. So our most able person is is she's done the, the other one with us. She goes out gardening, she goes out doing lots of things. But she's had a fall, because she was pulling weeds out and one was too strong. So it can easily be done. That then will knock her confidence. So I don't want her going on something like this that she wasn't that keen. Yeah. Okay. So they have to be very able" (Care Home 1)<br>"In fact, they because I've had to hold here. They would have to hold on really tight. Yeah. And because they're older this the grip alone." (Care Home 4)
	- *Incontinence*	"So you might have to look around that a little bit. Yeah. Especially with people that are already afraid of falling. Yeah, they're incontinent because they fall. They don't want you know, they don't want to go to the toilet, because so going on something like this might be a little bit of an issue." (Care Home 1)
	- *Influence of medication*	"I think it's the overall, you know, they are on lots of medication, how that's going to affect their balance. I've got some people who have vertigo, they wouldn't be able to do it" (Care Home 1)
	- *Limited mobility*	"This bit as well. I mean, we've got here they're not very mobile or anything." (Care Home 1)<br>"We're got a few that are quite mobile. Even the ones that could walk I think would really struggle." (Care Home 5)
	- *Problems with vision*	"A little bit. Getting better but it's still quite blurry though." (Care Home 4)
	- *Neck pain*	"I was like that well, going around rather than like that, that would that give you the neck neck ache and then that, okay, posture? Yeah" (Care Home 1)
	- *Neuromuscular*	*Interviewer*: "So thinking about maybe some of your family members, then what kind of conditions or challenges do you think a person would have that would mean this wasn't suitable?"<br>*Participant:* "Neuromuscular degeneration is one for me." (Workshop 1)
	- *Lived problem*	"would that be important that actually there was an ongoing service? Rather than you buy this and you're left on your own? Would you prefer it that actually, perhaps I pay a monthly fee? And I know, I can always pick up the phone if something goes wrong and contact that company? Would you rather kind of buy it and own it? Or have that kind of that long term rental maintenance and the service that comes with it?" (Workshop 2)

For peer review only - http://bmjopen.bmj.com/site/about/guidelines.xhtml

For peer review only

	- *Vertigo*	"For someone with that impairment, or someone with that illness, that's just not suitable for them. What about someone with vertigo?" (Care Home 2)
	Suitability for residents	"What do you think with the residents? I just I just said there's not many that we'll be able to put on that." (Care Home 5)
	- *99% not suitable*	"99% won't be able to do it anyhow" (Workshop 1)
	- *Suitable for residents 20 years younger*	"Twenty years ago when we had residents, like you can think we still got residents that would have been fine but now. No way" (Workshop 1)
	- *Wouldn't recommend to people with mobility issues*	"Unsteady gait in general, having vertigo, history of, history of falls in any sense, increasing that risk of. I wouldn't bother I think." (Workshop 1)
	Ethics	"There's still challenges, though, isn't there of getting on and off like exercise, you know, I've taken loads of people on it, like that, it can be quite a dodgy thing to do. It's not okay, so yeah, there's still challenges with getting older adults on and off these things, especially anyone who has cognitive impairment," (Workshop 1)
	Risk	"Older adults wouldn't be able to even step over that even though she's on it [...] And probably in nursing homes older people's standing tolerance may not be that. And the effect on balance from turning, as well as the nausea point of view" (Workshop 1)
	Size of technology	"It is massive. I didn't expect it to be this big." (Care Home 1)
	- *Wifi connectivity*	"It would probably be in the lounge because probably we get quite good reception in the lounge. Sometimes in parts of the building we might lose it at times." (Care Home 4)
	Familiarisation	"And just like anything else, it's time to get used to it. That familiarization with. But that's like everything that we have that's new. Once people are familiar with it, and it's normal. I mean, to our residents, this sort of technology is quite strange, anyway," (Care Home 2)
	- *Adapting to use*	"Because I'm, I've got a bad back. But I was able to still able to do it. I got poor eyes because I've got wet macular degenerate disease but it was fine. Okay, I couldn't do as long as what [other participants] did probably, but also it's my first time in VR so I was cautious but as you get used to it." (Care Home 5)
	- *Unfamiliar*	"I can just see a pillar here. I could hear someone it's like the pillars talking [...] It's like I'm disorientated" (Care Home 5)
	- *Adjusting to headset*	"And when the VR went on, very disorientating, very blurry, and for then to adjust it as they want it, I think might be an issue. So you need to be able to see what they're seeing on the computer to you maybe have to adjust it that" (Care Home 1)
	- *Familiarity*	"I certainly think the fact that you're going to have destinations, local destinations will be the attraction." (Workshop 2)
Improvements	Content suggestions	"Under the sea would be good wouldn't it?" (Care Home 5)<br>"We could take them to places. If we could get in the gardens every year. If you could capture that." (Care Home 4)<br>"Because you really you're taking them to places you know like the Beaches."(Care Home 4)
	- *Cartoon-like images*	"You want to touch the flowers, so they will let go and then they'll Yeah, yeah. So I do think that's a bit of a challenge mobility, and that's quite cartoonish as well. So will it be reality?" (Care Home 1)
	- *New environments*	"Yeah, Mount Edgecumbe. All those kinds of things that they they would love. Even the Tamar Bridge. A garden center. You know, last gardens of Helligen, Eden Project. I think they'd love it. Yeah, I think they'd really love it. And they're sitting down and they can look at the flowers. And, you know, pick, they can do all these kinds of things. But if they're standing up, I think there'll be more "I don't like it. I don't like it", because it's do you see what I mean? Yes. Yeah," (Care Home 1)
	Inclusivity	"how we could adapt the equipment for her typically she has full capacity and would really benefit from this but obviously it's a double amputee so she's not going to physically walk" (Care Home 3)
	- *Adaptable bar height*	"Yeah, even if that was that was, you could undo it and take that bit off. You only need an eye and a button don't you like you don't like the walking sticks with the eyes and the button you only need a click button and then you could make it different sizes as well. It could go back further, be adaptable. They have something to hold on to in front of them." (Care Home 5)

For peer review only

	- *Fitting to a wheelchair*	"Even if it's just like a normal chair. Yeah. Not a wheelchair. Because when you soon as you get in a wheelchair, you think you're going somewhere, which might be a good thing." (Care Home 1)
	- *People less mobile*	"You know, most of them come in in wheelchairs. Okay. That's the way residents are coming in." (Workshop 1)
	- *Access could be improved*	"You have to naturally be able to step up, don't you? So if you could step up onto like, just a something there, as a gap in between." (Workshop 1)
	- *Physical requirements*	"From a physical perspective. I have to say this is a dance of what they have been used to before, but I would think that whoever would need to, to safely do this would already have a degree of practicality about them." (Workshop 1)
	Safety improvements	"There's that feeling of going back. So I think if there were two front legs and at the back, that would give much more support?" (Care Home 3)<br>"I think you're looking at someone with a degenerative physical disease, but good cognition, once you've made some modification, so it doesn't kill them." (Workshop 1)
	- *Softer impact bars*	"If they do, if a resident slips out, as in legs come down. Obviously, we would need some sort of buffers over the top of the bars," (Care Home 5)
	- *Stop slipping*	"I don't know, do you need to have it slippery actually." (Care Home 5)
	- *Awfully slippery*	"Awfully slippery. So you're, you're like when you're walking on ice or wherever you're on guard. So that takes away the enjoyment." (Workshop 1)
	- *Difficulty exiting treadmill*	"I'm not sure how many people would be able to get out of that." (Workshop 1)
	Add arm movements	"You have like any interaction with hand stuff as well. You know you've got your feet Yeah, you do. So you have things in your hands so" (Care Home 5)
	Cast visual for group to watch	"Yeah. I don't know if we could connect to this screen, so the rest of them can actually see on the screen where this [in the VR] is." (Care Home 5)
	Seated version	"The seated unit would be more beneficial to our client base, as we are at the moment." (Care Home 2)<br>"It would have to be a sitting one, but would they like that [VR headset] on their head" (Care Home 6)
	- *Square*	"If it was sitting down. Yeah. Yeah, that would be different." (Workshop 1)
	- *Headset use only*	"I would think if it was less slippery, yeah. And maybe if you could, well, you couldn't just use the headset. Could you? Could you just use the headset?" (Care Home 1)
	- *Adaptations*	"Could they not sit? If they were sat using their feet Yeah. But then they wouldn't get do you have to. Is there a was a chair scenario." (Care Home 4)
	Possibilities/exercise bike	"I wonder about it on a bike because that way. You've got your bike, which turns, so pedalling is innate. People know peddling goes forwards. And so on. And if you could turn on that. I wonder if it would keep you a little bit more orientated? Because all your movement has been done with that and with the steering wheel aspect to that. And so I don't know if that would be better. If you're trying to get the exercise. Cause it's really interesting. You're looking at it from the exercise point of view. If it's just getting someone out and about." (Care Home 5)
Current design	Good content	"I'm just getting to the balcony. Oh my god. Oh that is amazing brilliant. It is amazing that is phenomenal." (Care Home 5)<br>"But it just feels so real" (Care Home 4)<br>"It's beautiful" (Care Home 6)
	- *Visual features*<br>- *Spatial features*	"No, I don't want to go down the steps. I'm gonna go this way. I don't want to go down the steps, I don't like it" (Care Home 4)
	Liked headset	"It doesn't feel heavy on my head" (Care Home 5)

For peer review only - http://bmjopen.bmj.com/site/about/guidelines.xhtml

		“Yeah, that's good, it feels really comfortable” (Care Home 4)
	Safety features	“That’s obviously why the bars are there” (Care Home 5)
	Technology issues	“However much walking I got I wasn't going anywhere.” (Care Home 5)
	- *Glitching*	“You’re actually flying all over the place […] you are there but you’re flashing all over it as well.” (Care Home 5)
	- *Loading worlds*	“All I got is clouds and a green line. It's just a green line.” (Care Home 5)
	- *Speed of movement*	“I'm guessing I would say that one slide of that foot? You'd probably moving about two feet.” (Care Home 5)
	Ergonomics	“I'm stepping in a way I wouldn't normally step” (Care Home 5)<br>“They didn't feel big on the feet. Because you're sliding” (Care Home 4)
	Mechanics	“It doesn't feel like natural walking.” (Workshop 2)
	Resolution	“it's going to feel a lot more stable with that in there, and so they've suggested that when people have their first experiences, it's always with the training mats in place.” (Workshop 2)

For peer review only

For peer review only - http://bmjopen.bmj.com/site/about/guidelines.xhtml

For peer review only

For peer review only - http://bmjopen.bmj.com/site/about/guidelines.xhtml

COREQ (COnsolidated criteria for REporting Qualitative research) Checklist

A checklist of items that should be included in reports of qualitative research. You must report the page number in your manuscript where you consider each of the items listed in this checklist. If you have not included this information, either revise your manuscript accordingly before submitting or note N/A.

Topic	Item No.	Guide Questions/Description	Reported on Page No.
**Domain 1: Research team and reflexivity**			
Personal characteristics			
Interviewer/facilitator	1	Which author/s conducted the interview or focus group?	
Credentials	2	What were the researcher's credentials? E.g. PhD, MD	
Occupation	3	What was their occupation at the time of the study?	
Gender	4	Was the researcher male or female?	
Experience and training	5	What experience or training did the researcher have?	
Relationship with participants			
Relationship established	6	Was a relationship established prior to study commencement?	
Participant knowledge of the interviewer	7	What did the participants know about the researcher? e.g. personal goals, reasons for doing the research	
Interviewer characteristics	8	What characteristics were reported about the inter viewer/facilitator? e.g. Bias, assumptions, reasons and interests in the research topic	
**Domain 2: Study design**			
Theoretical framework			
Methodological orientation and Theory	9	What methodological orientation was stated to underpin the study? e.g. grounded theory, discourse analysis, ethnography, phenomenology, content analysis	
Participant selection			
Sampling	10	How were participants selected? e.g. purposive, convenience, consecutive, snowball	
Method of approach	11	How were participants approached? e.g. face-to-face, telephone, mail, email	
Sample size	12	How many participants were in the study?	
Non-participation	13	How many people refused to participate or dropped out? Reasons?	
Setting			
Setting of data collection	14	Where was the data collected? e.g. home, clinic, workplace	
Presence of non-participants	15	Was anyone else present besides the participants and researchers?	
Description of sample	16	What are the important characteristics of the sample? e.g. demographic data, date	
Data collection			
Interview guide	17	Were questions, prompts, guides provided by the authors? Was it pilot tested?	
Repeat interviews	18	Were repeat inter views carried out? If yes, how many?	
Audio/visual recording	19	Did the research use audio or visual recording to collect the data?	
Field notes	20	Were field notes made during and/or after the inter view or focus group?	
Duration	21	What was the duration of the inter views or focus group?	
Data saturation	22	Was data saturation discussed?	
Transcripts returned	23	Were transcripts returned to participants for comment and/or	

For peer review only

Topic	Item No.	Guide Questions/Description	Reported on Page No.
		correction?	
**Domain 3: analysis and findings**			
Data analysis			
Number of data coders	24	How many data coders coded the data?	
Description of the coding tree	25	Did authors provide a description of the coding tree?	
Derivation of themes	26	Were themes identified in advance or derived from the data?	
Software	27	What software, if applicable, was used to manage the data?	
Participant checking	28	Did participants provide feedback on the findings?	
Reporting			
Quotations presented	29	Were participant quotations presented to illustrate the themes/findings? Was each quotation identified? e.g. participant number	
Data and findings consistent	30	Was there consistency between the data presented and the findings?	
Clarity of major themes	31	Were major themes clearly presented in the findings?	
Clarity of minor themes	32	Is there a description of diverse cases or discussion of minor themes?	

Developed from: Tong A, Sainsbury P, Craig J. Consolidated criteria for reporting qualitative research (COREQ): a 32-item checklist for interviews and focus groups. *International Journal for Quality in Health Care*. 2007. Volume 19, Number 6: pp. 349 – 357

Once you have completed this checklist, please save a copy and upload it as part of your submission. DO NOT include this checklist as part of the main manuscript document. It must be uploaded as a separate file.

For peer review only

BMJ Open

Staff perceptions towards virtual reality motivated treadmill exercise for care home residents: A qualitative feedback study with key stakeholders and follow-up interview with technology developer.

Journal:	*BMJ Open*
Manuscript ID	bmjopen-2023-073307.R2
Article Type:	Original research
Date Submitted by the Author:	11-Oct-2023
Complete List of Authors:	Bradwell, Hannah; University of Plymouth<br>Cooper, Leonie; University of Plymouth<br>Edwards, Katie; University of Plymouth, Faculty of Health<br>Baxter, Rory; University of Plymouth<br>Tomaz, Simone; University of Stirling<br>Ritchie, John; University of Stirling<br>Gaudl, Swen; University of Plymouth<br>Veliz-Reyes, Alejandro; University of Plymouth<br>Ryde, Gemma; University of Stirling<br>Križaj, Tanja; University of Plymouth<br>Warren, Alison; University of Plymouth<br>Chatterjee, Arunangsu; University of Plymouth, Centre of Health Technology; University of Leeds, School of Medicine<br>Willis, Katharine; University of Plymouth<br>Haynes, Richard; University of Stirling<br>Hennessy, Catherine; University of Stirling<br>Whittaker, Anna; University of Stirling<br>Asthana, Sheena; University of Plymouth<br>Jones, Ray; University of Plymouth
**Primary Subject Heading**:	Qualitative research
Secondary Subject Heading:	Geriatric medicine, Health informatics
Keywords:	Virtual Reality, Physical Therapy Modalities, Information technology < BIOTECHNOLOGY & BIOINFORMATICS

SCHOLARONE™
Manuscripts

I, the Submitting Author has the right to grant and does grant on behalf of all authors of the Work (as defined in the below author licence), an exclusive licence and/or a non-exclusive licence for contributions from authors who are: i) UK Crown employees; ii) where BMJ has agreed a CC-BY licence shall apply, and/or iii) in accordance with the terms applicable for US Federal Government officers or employees acting as part of their official duties; on a worldwide, perpetual, irrevocable, royalty-free basis to BMJ Publishing Group Ltd ("BMJ") its licensees and where the relevant Journal is co-owned by BMJ to the co-owners of the Journal, to publish the Work in this journal and any other BMJ products and to exploit all rights, as set out in our licence.

The Submitting Author accepts and understands that any supply made under these terms is made by BMJ to the Submitting Author unless you are acting as an employee on behalf of your employer or a postgraduate student of an affiliated institution which is paying any applicable article publishing charge ("APC") for Open Access articles. Where the Submitting Author wishes to make the Work available on an Open Access basis (and intends to pay the relevant APC), the terms of reuse of such Open Access shall be governed by a Creative Commons licence – details of these licences and which Creative Commons licence will apply to this Work are set out in our licence referred to above.

Other than as permitted in any relevant BMJ Author's Self Archiving Policies, I confirm this Work has not been accepted for publication elsewhere, is not being considered for publication elsewhere and does not duplicate material already published. I confirm all authors consent to publication of this Work and authorise the granting of this licence.

For peer review only

Staff perceptions towards virtual reality motivated treadmill exercise for care home residents: A qualitative feedback study with key stakeholders and follow-up interview with technology developer.

Bradwell, H.L[1*]. Cooper, L[1]., Edwards, K.J[1]., Baxter, R[1]., Tomaz, S.A[2]., Ritchie, J[2]., Gaudl, S[1]., Veliz-Reyes, A[1]., Ryde, G.C[2]., Krizaj, T[1]., Warren, A[1]., Chatterjee, A[1 3]., Willis, K[1]., Haynes, R[2]., Hennessy, C.H[2]., Whittaker, A.C[2]., Asthana, S[1]., Jones, R.B[1] on behalf of the GOALD project.

[1] Centre for Health Technology, University of Plymouth, Plymouth, UK.

[2] University of Stirling, Stirling, UK.

[3] School of Medicine, University of Leeds

*Corresponding author: Hannah.bradwell@plymouth.ac.uk

For peer review only

Abstract

Objectives
Health and care resources are under increasing pressure, partly due to the ageing population. Physical activity supports healthy ageing, but motivating exercise is challenging. We aimed to explore staff perceptions towards a virtual reality (VR) omnidirectional treadmill (MOTUS), aimed at increasing physical activity for older adult care home residents.

Design
Interactive workshops and qualitative evaluation.

Settings
Eight interactive workshops were held at six care homes and two university sites across Cornwall, England from September-November 2021.

Participants
Forty-four staff participated, including care home, supported living, clinical care and compliance managers, carers, activity coordinators, occupational therapists, and physiotherapists.

Interventions
Participants tried the VR treadmill system, followed by focus groups exploring device design, potential usefulness, or barriers for care home residents. Focus groups were audio recorded, transcribed verbatim and thematically analysed. We subsequently conducted a follow-up interview with the technology developer (September 2022), to explore feedback impact.

Results
Analysis produced seven key themes: anticipated benefits, acceptability, concerns of use, concerns of negative effects, suitability/unsuitability, improvements, and current design. Participants were generally positive towards VR to motivate care home residents' physical activity and noted several potential benefits (increased exercise, stimulation, social interaction, and rehabilitation). Despite reported potential, staff had safety concerns for

For peer review only - http://bmjopen.bmj.com/site/about/guidelines.xhtml

frail older residents, due to the standing position. Participants suggested design improvements to enhance safety, usability, and accessibility. Feedback to the designers resulted in development of a new seated VR treadmill to address concerns on falls while maintaining motivation to exercise. The follow-up developer interview identified significant value in academia-industry collaboration.

Conclusion
The use of VR motivated exercise holds potential to increase exercise, encourage reminiscence, and promote meaningful activity for care home residents. Staff concerns resulted in a re-designed seated treadmill for those too frail to use the standing version. This novel study demonstrates importance of stakeholder feedback in product design.

Key words: social care, physical activity, technology, virtual reality, innovation, knowledge exchange

Word count: 4342

Article Summary

Strengths and limitations of this study

- Relatively large sample of participants for qualitative research providing breadth of perceptions and data saturation
- Rigorous qualitative analysis on novel topic by a research team with diverse (yet, related to the topic) disciplinary backgrounds (including engineering, health, design)
- Explored perceptions of key stakeholders (health and social care staff)
- However, limited interaction durations between participants and the technology
- No perceptions gathered from older adult stakeholders

Background

Introduction

Pressure on health and social care (H&SC) resources is increasing, because of increased life expectancy and decreasing numbers of care workers [1, 2]. Therefore, there has been a push for research and innovation into technologies to support healthy ageing [3, 4]. Reducing loneliness and remaining active are key strands of improved wellbeing for older adults [5, 6]. Physical activity has numerous health benefits for older adults, helping reduce risks of diseases such as cancer, diabetes, cardiovascular disease and stroke, and delaying dementia onset [6]. Being consistently active for over six months has also been associated with reduced incidence of falls [7]. However older adults do little exercise and have difficulties maintaining exercise over time [6].

One technology of interest in supporting older people with meaningful activity, motivating exercise, and encouraging social connectivity is virtual reality (VR). There have been increasing numbers of studies exploring VR use within care settings [8]. Here we report a

For peer review only - http://bmjopen.bmj.com/site/about/guidelines.xhtml

study of a VR and omnidirectional treadmill system, MOTUS Adventure (MOTUS, formerly known as ROVR), designed for older adults [9]. The system is designed to motivate activity, physically navigating virtual environments that have meaning for the users. The use of environments with meaning to the user is essential to achieve the aim of meaningful activity, which, by definition, must be tailored to the users' preferences [10].

The VR environments in MOTUS include places of cultural and historical interest such as ancient forts, museums, nature scenes and cityscapes. These 3D virtual environments represent places care home residents may no longer be able to visit physically. Access to culture (including heritage and history) is a human right [11], with documented health and wellbeing benefits, including improved quality of life, opportunity for reminiscence and connectedness to spaces and history [12]. Older adults in particular are invested in heritage, yet physical access to sites of cultural or heritage interest is often limited due to site characteristics and conservation issues (e.g., uneven ground, inability to implement accessibility features) [13]. The Heritage Alliance annual report [12] recommended partnership between heritage and health, to overcome access challenges to help realise the full potential of heritage for health and wellbeing. The Covid-19 pandemic highlighted the potential for online access to culture and heritage in place of physical access [14], with VR offering possible solutions [15]. The use of VR to visit cultural and heritage sites can therefore aid in inclusivity for those with physical and sensory barriers [15], particularly prevalent among older adults. The opportunity for reminiscence brings additional wellbeing potential, with reminiscence thought to provide stimulation, enjoyment, sense of self-worth and achievement [16]. It should also be considered however, that some concerns have been raised on use of VR with older adults [8]. For example, older adults can have vision-related challenges, and motion sickness in VR specifically for older adults should be thoroughly explored in research with end-users. Future research should consider how to achieve the intended exercise intensity and access to heritage and culture while ensuring resident comfort over suitable durations of VR use.

Alongside potentially motivating physical activity and providing access to cultural and heritage sites, MOTUS allows for 'walks' to be socially interactive, with multiple users joining the same virtual environment to 'walk and talk' together, regardless of their location. The aim of the system therefore is to enhance social connectedness and improve physical health through meaningful activity [17]. However, prior to this study MOTUS had never been used in care homes. With any technology, there may be significant differences in opinions between developers and end-users [18] so older people's feedback in design of technology is essential. Given potential safety concerns and the need to understand how to implement the technology we first needed to explore care home staff opinions on feasibility, design, and potential for older care home residents.

Method

Aims

The aims of this study were to explore staff perceptions towards acceptability and feasibility of the MOTUS Adventure system for older adult care home residents, an understanding of

For peer review only - http://bmjopen.bmj.com/site/about/guidelines.xhtml

whether staff believe the technology will be feasible, acceptable or useful for care home residents is essential ahead of any direct feasibility study or implementation evaluation. Following study completion, results from the analysis were provided to the technology developer to help design iterations, based on staff feedback on potential improvements and design requirements.

Design

This study was a qualitative study based on technology interactions and focus group interviews, allowing staff to try, then to discuss, the equipment. Results were presented to the technology developer allowing them to modify the design. Results were presented verbally during meetings and in written form later, based on full analysis. We interviewed the Chief Executive about this and their experience of business working with research. This manuscript has been constructed following the COREQ checklist for qualitative research [19].

Location and dates

Data collection on perceptions of the MOTUS standing treadmill and VR system took place from September to November 2021 in Looe, Liskeard, Saltash, St Austell, Perranporth, Camborne, and Truro in Cornwall UK. Feedback (from the participants and our interactions) was provided to the developers at the time through verbal meetings and in written format via email. A follow-up interview with the technology developer took place via Zoom in September 2022.

Materials

Figure 1 shows the MOTUS Adventure omnidirectional treadmill, with a Pico Neo 2 VR headset to immerse the users in the virtual environments. The headset is equipped with a 4K LCD display that provides a resolution of 1920 x 2160 pixels per eye with a field view of 101 degrees. The treadmill is a smooth plastic dish coated thinly with lubricant on which people stand wearing overshoes with ceramic plates on the base. This allows for a low friction, slippery surface on which people 'slide' their feet backwards and forwards to represent walking. The treadmill links to the MOTUS PC software (in this study we ran the programme on a Lenovo ThinkPad L13) and VR system (Figure 1) via Bluetooth, which together, translates movement on the treadmill into movement through VR worlds. To change direction, participants swivel around and point their bodies in the direction of desired travel. VR worlds include 3D scanned, real world, heritage locations as well as animated worlds ranging from museums to simple houses (Figure 2). The MOTUS VR worlds can also be social experiences, with several users entering the same world from distant locations and walking and talking together. Within the workshops, participants were first offered the opporunity to walk in the simple, animated house, to become accustomed to the experience before trying more complex models such as the heritage locations or museum. Each participant tried at least two or three of the VR environments. The participant was always 'joined' in the VR world by a researcher (as an avatar) linking in to the social world through a computer and walking with them via keyboard controls, to allow demonstration of the social angle.

For peer review only - http://bmjopen.bmj.com/site/about/guidelines.xhtml

For peer review only

(Figure 1 and 2 here)

Procedure

Recruitment

Convenience sampling was used. The health professionals were recruited at University of Plymouth e-Health events, through approach by the researcher (HB). Health professionals read participant information sheets and signed written consent forms before engaging in the workshop. All health professionals had experience of older adult care in various forms, some with movement/exercise in particular (care homes, physiotherapy, occupational therapy). The care homes were approached by email by HB, chosen based on prior collaboration with the researcher, to scope interest in trying the MOTUS. For care home contacts who were interested, researcher visits were arranged, and participant information sheets and consent forms were provided. It is important to consider the impact of prior collaboration on results, although the participants may have had enhanced exposure to other technologies through prior work, they had not previously been involved in any VR studies.

Workshops

Two researchers (HB, KE, both PhD holders with a background in digital health research and specific interest on social care) engaged in eight interaction workshops where a total of 44 key stakeholders (health and care staff) were provided with demonstrations of the MOTUS Adventure and the opportunity to try the technology themselves. All staff tried the MOTUS having at least 5 minutes of interaction, although some interactions were longer (>15 minutes), and all staff watched as other staff used the technology, including via casting of the VR world onto a computer screen, to help further inform their opinions through increased exposure to the technology in use. We used audio recording devices to capture comments during use. All 44 staff then participated in focus group discussions to share their perceptions. These were also audio recorded. Sessions lasted from 43-110 (mean 71) minutes. Sessions had between three and eight participants (Table 1). We were unable to link participants to distant MOTUS users as a social experience due to feasibility but did demonstrate two users in one world (the researcher on laptop and participant on treadmill), to facilitate a discussion around the potential social experience. One of the workshops engaged health professionals such as occupational therapists and physiotherapists, and the other seven workshops engaged care home staff and managers.

For the one follow-up interview (approximately 10 months after the data collection and feedback to the business based on participant perceptions), the technology developer was invited to take part in an informal interview to discuss their perceptions of the impact of the academic feedback from the focus groups on their products and processes. The interview was conducted by a separate researcher (AW) to those who had been collaborating with the technology developer for the purpose of this study, to avoid bias. The interview was one hour in duration.

Ethics

For peer review only - http://bmjopen.bmj.com/site/about/guidelines.xhtml

This study received ethical approval from the Faculty of Health Ethics Committee at the University of Plymouth, project ID 2887 (August 2021). All participants gave written informed consent.

Patient and public involvement

Considering the timing of this study, with health and care staff capacity still limited due to ongoing effects of the Covid-19 pandemic, it was not possible to involve them in the design, reporting or dissemination of this research.

Data Collection

Stakeholders provided feedback during their interaction with the MOTUS Adventure treadmill and following the interaction during informal focus groups (interview schedule available in Appendix A). We used a semi-structure interview approach, following the schedule but deviating for natural conversation and follow-up questions on interesting comments. The feedback was audio-recorded and transcribed verbatim. The interview with the technology developer was also audio recorded and transcribed verbatim, there was no interview schedule used, but general queries posed to the developer on their perceptions towards the feedback from this study.

Data Analysis

Researchers (HB, LC, RB, ST, JR, AW, TK, SG, ARV) analysed the transcripts using thematic analysis. Braun et al. [20] detail the process of thematic analysis, a form of analysing qualitative data that is often used in health and social sciences. As prescribed, the researchers first became familiar with the data set (through reading and understanding the full transcripts), before generating initial codes upon re-reading and looking for meaning, searching for themes across the codes from all transcripts, and reviewing and defining those themes. The sub-type of thematic analysis used was reflexive, in generating codes from explicit content, which evolves and adapts through considerable analytic work, to produce themes, representing an understanding of meaning across a dataset [20]. Analysis was inductive, as researchers had pre-determined aims, and therefore were seeking understanding of participant perceptions towards the device among the data. Analysis was conducted by nine researchers due to the size of the dataset, with a considerable amount of qualitative data collected. All nine researchers conducted initial coding, with transcripts divided between researchers, then HB, LC and RB led on iterations of reviewing and defining themes, however, the whole research team had two meetings to explore different perspectives on the data and codes to enhance validity of interpretation through perspectives from multi-disciplinary expertise (occupational therapy, psychology, physical activity science, computer science, social care research, digital design). The range of experience provided ensured broad perspectives were considered in our interpretations. We used the NVivo 12 data analysis software for data management to ensure a clear audit trail, enhancing dependability [21]. The involvement of a multidisciplinary team with various expertise in the data analysis processes enhances the credibility of our Results [22], the researchers who conducted analysis had diverse (yet, related to the topic) disciplinary

For peer review only - http://bmjopen.bmj.com/site/about/guidelines.xhtml

backgrounds (including engineering, health, design), increasing robustness and significance of results (all researchers included have or are working towards a PhD and strong experience in data analysis). The process for the analysis of the follow-up interview with the technology developer was the same, although conducted by GR, AW and HB.

Participants

In total, across the eight interaction workshops, 44 participants interacted with the MOTUS systems VR treadmill and provided evaluative feedback (Table 1). The participants included provided a broad range of appropriate stakeholders for English care homes, including across all levels of the operational care home staff and additionally various health professionals with expertise relevant for movement and activity (e.g. occupational therapist and physiotherapist). For the follow-up interview with the technology developer there was one participant, the chief operating officer of the MOTUS Systems technology developer.

Table 1: Participants at each interaction workshop

Site	Participants (n)
Workshop at University site 1	(5) General health and care professionals including occupational therapist, physiotherapist and patient involvement expert
Workshop at University site 2	(3) Supported living manager and care home staff
Workshop at Care home 1 – 26 bed residential care home	(3) Care home manager, compliance manager, activity coordinator and carer
Workshop at Care home 2 – 83 bed nursing home with unit for people with dementia	(7) Care home compliance manager, clinical needs manager, activity coordinator and carers
Workshop at Care home 3 – 47 bed care home for people with dementia	(6) Care home manager and carers
Workshop at Care home 4 – 33 bed care home	(8) Care home manager and carers
Workshop at Care home 5 – 40 bed care home	(7) Care home manager and carers
Workshop at Care home 6 – 60 bed nursing home for people with dementia	(5) Residential care nursing manager, carers

Results

Section 1: Workshop results

Here we discuss the results of the eight interactive workshops exploring stakeholder perceptions towards the VR motivated MOTUS treadmill. In section 1, quotes have been identified by site where the data were collected rather than by individual participant. The roadshow/workshop nature of data collection and recording meant identifying individuals within busy transcripts was not feasible. In section 2, we share the results of the follow-up

For peer review only - http://bmjopen.bmj.com/site/about/guidelines.xhtml

interview with the technology developer. Table 2 shows the themes and codes identified in the analysis of the eight workshops.

Table 2: The results of thematic analysis of the 8 interaction workshops. Evidence to support each code can be found in Appendix B and is synthesised in narrative below.

**Theme**	**Initial Codes, *Sub-codes***
Anticipated benefits	Virtual visits; *Interesting local sites, Virtual tourist sites*<br>Physical activity/exercise; *Motivating exercise*<br>Prevents boredom<br>Rehabilitation<br>Staff use<br>Social interaction<br>Wellbeing<br>Accessible experiences; *Different to care home*<br>Stimulates the brain<br>Restorative<br>Reminiscence; *Reminiscence benefits*<br>Expectations challenges positively<br>Positive distraction and novelty
Acceptability	Positive comments<br>MOTUS is interesting<br>Adoption desired<br>Ease of use; *Headset initially heavy but comfortable, Physically easy, Treadmill is easy to enter, Treadmill is easy to move on, VR is novel experience*
Concerns of use	Secureness<br>Safety concerns; *Hitting objects, Shoes, Safety hazards, Danger*<br>Lack of control; *Movement issues*<br>Risk of falls/Dangerous; *History of falls, Could cause falls, Risk of injury - hips*<br>Accessibility<br>Prefer people go out<br>Heavy shoes<br>Lack of motivation<br>Uncomfortable movement<br>Bigger environment more physically demanding<br>Critique<br>Potential issues
Concerns of negative effects	Side effects; *Encouraging shuffling/sliding, Unnatural movement*<br>Dizziness<br>Disorientating; *Orienting to environment, Coordination between VR and feet, Height changes, Motion, Social angle confusing*<br>Concentrating more on the exercise<br>Depth perception difficulties<br>Motion sickness in VR<br>Fear

For peer review only - http://bmjopen.bmj.com/site/about/guidelines.xhtml

Suitability/Unsuitability	Extra support; *Supervised use required, Guidance and guided navigation, Human resources, Number of residents, Differences between residents, Residents unable to use*<br>Diagnosis; *Balance, Dementia/cognition, Fear of falling, Have to be able, Incontinence, Influence of medication, Limited mobility, Problems with vision, Neck pain, Neuromuscular, Lived problem, Vertigo*<br>Suitability for residents; *99% not suitable, Suitable for residents 20 years younger, Wouldn't recommend to people with mobility issues*<br>Ethics<br>Risk<br>Size of technology; *WiFi connectivity*<br>Familiarisation; *Adapting to use, Unfamiliar, Adjusting to headset, Familiarity*
Improvements	Content suggestions; *Cartoon-like images, New environments*<br>Inclusivity; *Adaptable bar height, Fitting to a wheelchair, People less mobile, Access could be improved, Physical requirements*<br>Safety improvements; *Softer impact bars, Stop slipping, Awfully slippery, Difficulty exiting treadmill*<br>Add arm movements<br>Cast visual for group to watch<br>Seated version; *Square, Headset use only, Adaptations*<br>Possibilities/exercise bike
Current design	Good content; *Visual Features, Spatial Features*<br>Liked headset<br>Safety features<br>Technology issues; *Glitching, Loading worlds, Speed of movement*<br>Ergonomics<br>Mechanics<br>Resolution

Anticipated Benefits

Participants noted a range of perceived benefits of using MOTUS, with many focusing on the potential for physical benefits, such as providing "*good exercise*" (Care Home 2) and being "*particularly […] fantastic for rehab*" (Care Home 5). Participants noted that finding motivation to complete standard exercises can be a "*struggle*" and "*boring*" (Care Home 5), while the VR content would mean "*they can actually walk on that, something to motivate them to do it*" (Care home 5).

Participants also noted the potential enhanced accessibility of "*tourist sites*" (Care Home 5), enhancing visiting potential, particularly for those with "*reductions in mobility*" (Care Home 1). One staff member reported "*some [residents] are just not able to get out anymore […] you're providing the opportunity to see the place that they love*" (Care Home 4). This experience was additionally expected to create "*enhanced communication*" (Care Home 5)

For peer review only - http://bmjopen.bmj.com/site/about/guidelines.xhtml

prompting conversations between residents with each other and the staff. Care staff additionally felt that reminiscence could be an additional benefit as it may "*trigger memories*" for the residents. (Care Home 2).

Acceptability

Further to the range of potential benefits noted by care staff, participants generally demonstrated good acceptability towards the device, often praising the technology as "*amazing, absolutely amazing*" (Care Home 3), "*honestly I think it's amazing*" (Care Home 6). One care home manager even reported that she would "*buy it for the staff and residents […] so that we actually had one permanently*" (Care Home 5).

Concerns of Use

Despite the general acceptability and praise towards the concept, and appreciation for potential benefits, participants noted some concerns about suitability of such devices for older adults and the care setting. Most concerns related to the safety of vulnerable older adults using a low-friction, stand-up treadmill. One care home manager reported that for care home residents specifically, the design was "*unsafe*" (Care Home 4), with another suggesting it was "*a danger*" due to being "*slippery*" (Care Home 5). The worry was that generally care home residents are "*not as agile as they used to be*" and could "*potentially fall*" (Care Home 3). This could result in injuries to "*ribs […] or your arm, break an arm*" (Care Home 5), with one carer suggesting "*I can see a few hips going*" (Care Home 2). As a result of these concerns, participants felt they would not want residents using the treadmill "*without a staff member*" (Workshop 2).

Concerns of Negative Effects

Beyond some safety concerns of residents using a stand-up, slippery treadmill, broader concerns were raised around "*motion sickness*" (Care Home 3), feeling "*dizzy*" (Care Home 5), and unnatural movements "*because you're not stepping*" (Care Home 5), "*it'll feel a bit weird*" (Care Home 6).

Suitability/Unsuitability

Participants debated the older adults in their care who MOTUS would and would not be best suited to. Based on the concerns presented above, care home staff echoed that residents would "*need to have much more support*" to use the treadmill (Care Home 3), and to be "*supervised*" (Care Home 2), which would mean "*we're going to have to get the staff on board*" (Workshop 2). The treadmill was felt to be more suitable for residents with "*more stability when they're on their feet*" (Care Home 5), while care staff listed diagnoses that would make the standing treadmill less suitable. Suitability concerns were raised for people with "*lots of medication [that] affects their balance*" (Care Home 1), people with "*dementia*" (Care Home 4), who could "*totally freak*" (Care Home 6) and people with "*vertigo*" (Care Home 1). Despite the concerns, participants acknowledged that "*just like anything else, it's time to get used to it*" (Care Home 2), suggesting that prolonged use may encourage more confidence.

For peer review only - http://bmjopen.bmj.com/site/about/guidelines.xhtml

Improvements

Although participants were generally accepting and appreciative of the concept of VR to motivate physical activity, and acknowledged the potential benefits, the stakeholders were conscious of potential safety concerns which led to numerous participants suggesting a re-design of the treadmill to a "*seated unit*" which would "*be more beneficial to our client base*" (Care Home 2). Care staff suggested less mobile residents could be "*sat using their feet*" (Care Home 4) on a flat platform, to navigate the VR worlds without the risk of the standing, low-friction treadmill. Participants felt this would improve inclusivity of the experience as "*most [residents] come in in wheelchairs*" (Care Home 1). With these adaptations in mind, participants also shared thoughts on desired content for inclusion as a VR world, such as "*gardens*" (Care Home 4), "*under the sea*" (Care Home 5), or "*beaches*" (Care Home 4).

Current Design

While care stakeholders noted required adaptations to use VR to motivate movement and exercise safely for the target population of older adults and care home residents, participants praised aspects of the current design, including the VR content itself as "*amazing, brilliant, it is amazing, that is phenomenal*" (Care Home 5). They felt the headset "*doesn't feel too heavy on my head*" (Care Home 5), but "*feels really comfortable*" (Care Home 4). Participants did also note some technical glitches to be overcome, such as not "*going anywhere*" (Care Home 5) when walking, and "*flashing all over*" (Care Home 5). These forms of issue were reported to the developers for bug fixes during the study period.

Section 2: Results of the follow-up interview with the technology developer

Quotes have not been provided with an identifier as only one participant took part in this follow-up interview. Table 3 provides the main themes and initial codes and sub-codes resultant from analysis.

Table 3: The results of thematic analysis of the follow-up interview with the technology developer. Evidence to support each code is synthesised in narrative below.

Theme	Initial Codes, *Sub-codes*
Benefits	Beneficial outcomes; P*hysical benefits, Social benefits, Mental benefits, Pushing personal boundaries, Benefits of Working with the University*
Process of development	Technology development; *Seated version*<br>Considering inclusivity; *Age, Care homes, Medical issues, Different sectors*<br>Phases of product development
Challenges	Cost and resources

Benefits

The follow-up interview with the technology developer highlighted a range of benefits born from the industry-academia collaboration. This included an initial code directly relating to

For peer review only - http://bmjopen.bmj.com/site/about/guidelines.xhtml

benefits of working with the University as the participant reported it was "*really helpful*" through the opportunity to *"apply the minimum viable product [and figure] out where it works and where it doesn't"* stating *"you get really fast feedback."* The developer felt academic collaboration and real-world feedback from stakeholders provided *"feedback we can work with."* The participant stated: "*it's really important for commerce [industry] to understand this, they need to work with people who are already experts*" as the participant felt universities *"have the freedom to do what most companies can't."* In this case, the technology developer benefited from the extensive experience the researchers had in working within social care and with older adults, *"you were working in this environment and already had that knowledge and understood the challenges involved."* The developers felt bringing their specific technical knowledge and combining it with the university's evaluation expertise meant "*we didn't have to […] learn it ourselves […] or teach you […], all we had to do was provide the equipment and gain feedback."* The developer felt having evaluation through the University provided a "*helpful," "independent view" "which is unbiased."*

The technology developer also noted benefits they perceived of the technology itself, based on the interactions during the study period and subsequent collaborative studies (to be published elsewhere). The developer discussed how older adults in general can need "*motivation to exercise*" with one particular challenge "*in the Northern hemisphere*" being "*winters [which are] long and wet and dark*" leading to older adults becoming "*deconditioned over the winter."* Technologies such as MOTUS provide an indoor solution to motivate physical activity, which could be achieved "*on a daily basis."* The developer also shared the benefits of the potential social connection facilitated within the MOTUS VR worlds, "*they [older adult] visited [cultural location in VR] before, they'd love to visit. They can meet up with friends, they don't have to be in the same location, they can join, without necessarily having VR equipment yeah. And that makes this a social interaction."* Beyond the physical activity and potential for social contact, the developer felt they observed that the technology *"cognitively de-stressed*" older adults, meaning a range of potential benefits were observed.

Process of development

During the interview, the technology developer detailed how feedback from the current study helped lead to development of a seated version of the MOTUS treadmill (MOTUS Explore), which they felt was particularly required for "*the level of condition we found in care homes*" as "*they're really seated […] without major assistance for a long time*" meaning they couldn't use the standing MOTUS. The participant stated: "*one of the limitations, […] is the inability to move around safely*." The developer responded with "*a plate, which could be sat in front of people if they were seated and enable them to move."* The participant stated: "*it was a real opportunity to include people who would otherwise be excluded*" demonstrating significant value in this insight and product development as a result of the collaboration.

Challenges

For peer review only - http://bmjopen.bmj.com/site/about/guidelines.xhtml

The technology developer also shared some thoughts on challenges they face generally, such as cost. Before 2012, VR development was only for those *"with a million pound budget*," and though costs have fallen dramatically the feeling is that "*costs of development are still pretty high at present*" with "*various challenges around the world adding to those costs"*. Development costs and associated challenges further highlight the value of evaluation, iterative design and co-development with stakeholders, ensuring early developments are suitable and desirable for the intended audience before funds are wasted on inappropriate mass production.

Discussion

This study explored health and care staff perceptions towards the acceptability, feasibility and potential for VR motivated treadmill exercise for older adult care home residents, which is important due to the impact of the ageing population and growing burden of disease [2]. Research into the potential for technological innovations to support healthy ageing is essential to help respond to these challenges [3]. Our study adds to the literature on design of technology to encourage and support physical activity and exercise in older adults and care home residents.

Novel technologies such as the MOTUS have potential to support healthy ageing. Our results demonstrate clear promise for such technologies to motivate and sustain increased physical activity in older adults and people living in care homes, with our large sample of care staff generally agreeing on potential benefits for exercise and activity. Implementation of products such as these would likely have benefits for meaningful activity, meeting the NICE [10] definition of providing an activity that promotes health and wellbeing through an activity with meaning to the individual, and is tailored to their preferences. Our results have demonstrated an interest and preference among stakeholders for cultural, heritage and natural sites for use as a meaningful VR experience. Further work is now required with older adults themselves to confirm their preference for such an activity.

Staff also perceived benefits for social interaction, through both the social aspect of the VR platform but additionally through increases in residents talking to each other, their families and carers with the technology experience providing a focal point. This could have implications for the experience of loneliness among the target population [5, 23]. Our results suggest the technology was also expected to allow for reminiscence and enhanced experiences, through allowing older adults and care home residents to 'visit' places and heritage sites they could no longer access. Staff also noted that older adults may be able to 'visit' places significant to them through memories from the past. The benefits of access to heritage locations are well documented [12], therefore future research could explore and document the impact virtual access that is paired with technology that involves exercise to move around heritage sites has on health and wellbeing outcomes. Further exploration is also required to quantitatively assess the impact of the physical activity (or motivation to exercise) over longitudinal periods on outcomes for older adults. Additional future research should also seek to document the technical competence required for care staff to implement and facilitate such technologies without researchers present.

For peer review only - http://bmjopen.bmj.com/site/about/guidelines.xhtml

However, despite the positive perceptions towards the concept of VR to motivate activity and use of some form of treadmill to encourage movement and muscle use, care staff raised concerns around product design, mostly related to safety. Participants were concerned that for the frailer older adults and care home residents, standing on the low-friction treadmill could present a falls risk. When results were provided to the technology developer, this study had immediate implications for product design. Based on this stakeholder feedback, the MOTUS Systems team subsequently developed the Explore system, a seated version of the omnidirectional treadmill which would not present a falls risk while still motivating activity and lower leg movement to build up leg strength. The seated Explore system is currently undergoing evaluation with stakeholders as part of the ongoing GOALD project [24]. Beyond this immediate effect, this study has broader implications for those designing physical activity technologies aimed at supporting aged care. It demonstrates that care stakeholders are open and accepting to such technologies, believing the concept to be positive, while also providing useful design, hardware, and content insight for future developments. This value is clearly demonstrated in this study through the follow-up interview with the developer, and subsequent new product development.

This study also reinforces previous work [1, 18], showing the importance of engaging stakeholders in the design and testing of technologies aimed at health and care. The follow-up technology developer interview showed the value of academic-industry collaboration. The developers were able to use these Results to adapt the technology into a seated treadmill, resulting in an enhanced safety experience for those unable to comfortably stand and to increase the accessibility of the experience and its potential health and wellbeing benefits.

Strengths and limitations

Our study achieved a relatively large sample for qualitative work and gained deep and thorough insight into care staff perceptions. Our analysis used rigorous methods and the topic of enquiry was novel, in exploring VR motivated physical activity specifically for older adult care home residents.

The strong qualitative approach produced data that was substantial and complex, and although the themes and categories presented here encompass all data, there is additional evidence of more profound and complex forms of engagement with VR technologies, such as speculative feedback or feelings of telepresence. These additional dimensions warrant further investigation.

There were two limitations. First, the duration for which participants interacted with MOTUS was short. Our Results therefore may be over optimistic and influenced by the novelty effect. Longitudinal work would have better helped identify barriers and facilitators to use that would not be immediately recognised. The concerns of negative effects reported in our results, such as dizziness and motion sickness, would likely have been better assessed in longitudinal work, allowing time for participants to familiarise and overcome any initial negative sensations. These types of reactions are common among all VR systems, with work underway to generally reduce cybersickness experiences for users of head mounted displays

For peer review only - http://bmjopen.bmj.com/site/about/guidelines.xhtml

[25]. Research has shown that repeated exposure to the same VR content will reduce severity of motion sickness [25], thus a longitudinal or repeated measures study would more accurately report on the degree of barrier presented by the unpleasant sensations. As with the potential reduction in motion sickness over time, participants themselves noted that it would take time to gain confidence in using the MOTUS device with older adults and care home residents, therefore demonstrating potential to be more accepting over time.

Secondly, we only sought staff opinions in the potential future use of these devices, rather than older adults themselves. However, for devices such as these to be used by older adult care home residents, as intended by the developers, they first need to be accepted and adopted by the staff team who would be responsible for buying and facilitating use. To overcome both the limitation of short interaction period and staff stakeholders only, we have conducted a 6-week implementation study with the MOTUS device to be reported separately. It should also be considered that we did not explore device cost or site investment potential, therefore for real world implementations, cost would be a consideration.

Conclusion

The use of VR motivated exercise on an omnidirectional treadmill holds potential based on the perceptions of care staff for increasing exercise of older care home residents, while also providing virtual access to heritage sites, encouraging reminiscence and social connectivity, and promoting participation in a meaningful activity. A standing device however was not appropriate for a care home population and a new design is now being used for research with care home residents. User-centred design of health and care technologies is essential, and this study has demonstrated significant benefits for industry partners in collaborating with university institutions for independent evaluation with end-users.

Funding

This work was supported by UKRI Healthy Ageing Social, Behavioural and Design Research Programme grant number ES/V016113/1 as the project was completed by researchers on the GOALD project and the project also purchased MOTUS equipment for the study, with further support from the EPIC (eHealth Productivity and Innovation in Cornwall and the Isle of Scilly) project which was part funded by the European Regional Development Fund. Additional funding for the EPIC project was received from University of Plymouth. ERDF Grant number [05R18P02814], as EPIC also purchased some of the MOTUS equipment used. Finally, support was also provided by Innovate grant 10004423, which funded development, equipment and support provided by MOTUS Systems Ltd.

Competing interests

The authors declare no competing interests.

Author Contributions

HB and KE led on the design of the research. HB, KE, LC, AW and RB conducted data collection. HB, LC, RB, AW, TK, ST, JR, GR, SG and AVR conducted data analysis. RJ oversaw

For peer review only - http://bmjopen.bmj.com/site/about/guidelines.xhtml

the project design, completion and substantively contributed towards the write up, which was led by HB. SA, KW, CH, ACW, AC and RH oversaw study design and completion as GOALD senior management with RJ and reviewed and contributed towards the manuscript. All authors reviewed, edited and agreed to the final manuscript.

This project has received funding from the UKRI and ESRC Healthy Ageing Social Behavioural and Design Research Programme (SBDRP) grant No ES/V016113/1.
https://www.plymouth.ac.uk/research/centre-for-health-technology/goald

on behalf of the GOALD project: Catherine H. Hennessy, Faculty of Social Sciences, University of Stirling, Ray B. Jones, Faculty of Health, University of Plymouth, Richard Haynes, Faculty of Arts & Humanities, University of Stirling, Anna C. Whittaker, Faculty of Health Sciences & Sport, University of Stirling, (Core Management Team) and Sheena Asthana, Plymouth Institute for Health and Care Research (PIHR), University of Plymouth, Rory Baxter, Faculty of Health, University of Plymouth, Hannah Bradwell, Faculty of Health, University of Plymouth, Arunangsu Chatterjee, Faculty of Medicine and Health, University of Leeds, Pete Coffee, School of Social Sciences, Heriot-Watt University, Leonie Cooper, Faculty of Health, University of Plymouth, Alison Dawson, Faculty of Social Sciences, University of Stirling, Katie Edwards, Faculty of Health, University of Plymouth, Swen Gaudl, Faculty of Science & Engineering, University of Plymouth, Tanja Krizaj, Faculty of Health, University of Plymouth, Gregory Mannion, Faculty of Social Sciences, University of Stirling, Gemma Ryde, Institute of Cardiovascular and Medical Sciences, University of Glasgow, Alejandro Veliz Reyes, Faculty of Arts, Humanities and Business, University of Plymouth, John Ritchie, Faculty of Arts & Humanities, University of Stirling, Simone Tomaz, Faculty of Health Sciences & Sport, University of Stirling, Alison Warren, Faculty of Health, University of Plymouth, Karen Watchman, Faculty of Health Sciences & Sport, University of Stirling, Katherine Willis, Faculty of Arts, Humanities and Business, University of Plymouth, and partners - Active Stirling Ltd., Generations Working Together, Hearing Loss Cornwall, Nudge Community Builders, South Asian Society (Devon and Cornwall), Sporting Heritage, Sports Heritage Scotland, St. Breward Community, iSightCornwall, UKActive.

Acknowledgements

We would also like to thank our collaborating care homes for hosting our workshops and colleagues on the EPIC project who helped establish the collaboration.

Data sharing

Anonymised data is available from the corresponding author at reasonable request.

Ethics

This study received ethical approval from the Faculty of Health Ethics Committee at the University of Plymouth, project ID 2887 (August 2021). All participants gave written informed consent.

References

For peer review only - http://bmjopen.bmj.com/site/about/guidelines.xhtml

1. Moyle, W., Jones, C., Pu, L., & Chen, S.-C. (2018). Applying user-centred research design and evidence to develop and guide the use of technologies, including robots, in aged care. Contemporary nurse, 54(1), 1-3. doi:10.1080/10376178.2017.1438057

2. Abdi, J., Al-Hindawi, A., Ng, T. & Vizcaychipi, M.P. (2018). Scoping review on the use of socially assistive robot technology in elderly care. BMJ Open, 8(2), e018815.

3. UK Parliament (2018). Robotics in Social Care. Houses of Parliament, Post Note. [Internet] Available from: https://post.parliament.uk/research-briefings/post-pn-0591/

4. Garçon, L., Khasnabis, C., Walker, L., Nakatani, Y., Lapitan, J., Borg, J., et al. (2016). Medical and Assistive Health Technology: Meeting the Needs of Ageing Populations. Gerontologist, 56, Suppl 2:S293-302.

5. Siniscarco, M. T., Love-Williams, C., & Burnett-Wolle, S. (2017). Video Conferencing: An Intervention for Emotional Loneliness in Long-Term Care. Activities, Adaptation & Ageing, 41(4), 316-329. doi:10.1080/01924788.2017.1326763

6. Langhammer B, Bergland A, Rydwik E. The Importance of Physical Activity Exercise among Older People. Biomed Res Int. 2018 Dec 5;2018:7856823. doi: 10.1155/2018/7856823. PMID: 30627571; PMCID: PMC6304477.

7. Sherrington, C., Michaleff, Z. A., Fairhall, N., Paul, S. S., Tiedemann, A., Whitney, J., ... & Lord, S. R. (2017). Exercise to prevent falls in older adults: an updated systematic review and meta-analysis. British journal of sports medicine, 51(24), 1750-1758. http://dx.doi.org/10.1136/bjsports-2016-096547

8. To-Miles F, Mann J, Hung L (2022). Facilitators and barriers to using virtual reality and augmented reality and its impact on social engagement in aged care settings: a scoping review protocol. BMJ Open, 12, e061722. doi: 10.1136/bmjopen-2022-061722

9. MOTUS (2021). Step into a new reality: MOTUS systems. [Online] [Accessed 4/11/2021] Available from: https://motusvr.com/

10. NICE (2013). Mental wellbeing of older people in care homes. Quality standard QS50. Available from: https://www.nice.org.uk/guidance/qs50/chapter/quality-statement-1-participation-in-meaningful-activity#:~:text=Meaningful%20activity%20includes%20physical%2C%20social,crafts%2C%20conversation%2C%20and%20singing. [accessed 13/02/23].

11. UNESCO (2021). Right to participate in cultural life. UNESCO. [Online] [Accessed 2/11/2021]. Available from: https://en.unesco.org/human-rights/cultural-life

12. Heritage Alliance (2020). Heritage Alliance Annual Report. [Online] Available from: https://www.theheritagealliance.org.uk/wp-content/uploads/2020/10/Heritage-Alliance-AnnualReport_2020_Online.pdf

13. Marasco, Alessandra & Balbi, Barbara. (2019). Designing accessible experiences for heritage visitors through virtual reality. e-Review of Tourism Research. 17.

For peer review only - http://bmjopen.bmj.com/site/about/guidelines.xhtml

14. Kużelewska, E., Tomaszuk, M. European Human Rights Dimension of the Online Access to Cultural Heritage in Times of the COVID-19 Outbreak. *Int J Semiot Law* (2020). https://doi.org/10.1007/s11196-020-09712-x

15. Paladini, A., Dhanda, A., Ortiz, M. R., Weigert, A., Nofal, E., Min, A., … Quintero, M. S. IMPACT OF VIRTUAL REALITY EXPERIENCE ON ACCESSIBILITY OF CULTURAL HERITAGE. ISPRS - International Archives of the Photogrammetry, Remote Sensing and Spatial Information Sciences, XLII-2/W11, 929–936. https://doi.org/10.5194/ISPRS-ARCHIVES-XLII-2-W11-929-2019

16. Dempsey, L., Murphy, K., Cooney, A., Casey, D., O'Shea, E., Declan, D., Jordan, F. and Hunter, A. (2014), 'Reminiscence in dementia: a concept analysis', Dementia, 13 (2), pp. 176-92, doi: 10.1177/1471301212456277

17. Smith, N., Towers, A-M., Plamer, S., Beecham J. & Welch, E. (2018). Being occupied: supporting 'meaningful activity' in care homes for older people in England. Cambridge University Press.

18. Bradwell, H.L., Edwards, K.J., Winnington, R., Thill, S. & Jones, R.B. (2019). Companion robots for older people: importance of user-centred design demonstrated through observations and focus groups comparing preferences of older people and roboticists in South West England. BMJ Open, 9(9), e032468.

19. Tong A, Sainsbury P, Craig J. (2007). Consolidated criteria for reporting qualitative research (COREQ): a 32-item checklist for interviews and focus groups, International Journal for Quality in Health Care, 19(6), 349–357. doi.org/10.1093/intqhc/mzm042

20. Braun, V., Clarke, V., Hayfield, N. & Terry, G. (2019). Thematic analysis. In Liamputtong, P. (Ed.). Handbook of Research Methods in Health Social Sciences. https://doi.org/10.1007/978-981-10-5251-4_103

21. Bonello, M., Meehan, B. Transparency and coherence in a doctoral study case analysis: reflecting on the use of NVivo within a 'Framework' approach. The Qualitative Report. 2019;24(3):483-98

22. Morse, J.M., Field, P.A. Qualitative research methods for health professionals. 1995.

23. Campaign to End Loneliness (n.d.). The facts on loneliness. [Online] Available from: https://www.campaigntoendloneliness.org/the-facts-on-loneliness/

24. University of Plymouth (2022) Generating Older Active Lives Digitally. [Online] Available from: https://www.plymouth.ac.uk/research/centre-for-health-technology/goald [accessed 01/03/23].

25. Chang, E., Kim. H.T. & Yoo, B. (2020) Virtual Reality Sickness: A Review of Causes and Measurements, International Journal of Human–Computer Interaction, 36:17, 1658-1682, DOI: 10.1080/10447318.2020.1778351

Figure legends

For peer review only - http://bmjopen.bmj.com/site/about/guidelines.xhtml

Figure 1: (Left) Care home staff member interacting with the MOTUS Adventure treadmill with researcher recording initial reactions. (Right) VR headset with the MOTUS Adventure omnidirectional treadmill and example of the app to access VR worlds

Figure 2: Example of the virtual museum participants could explore by walking on the MOTUS Adventure

For peer review only

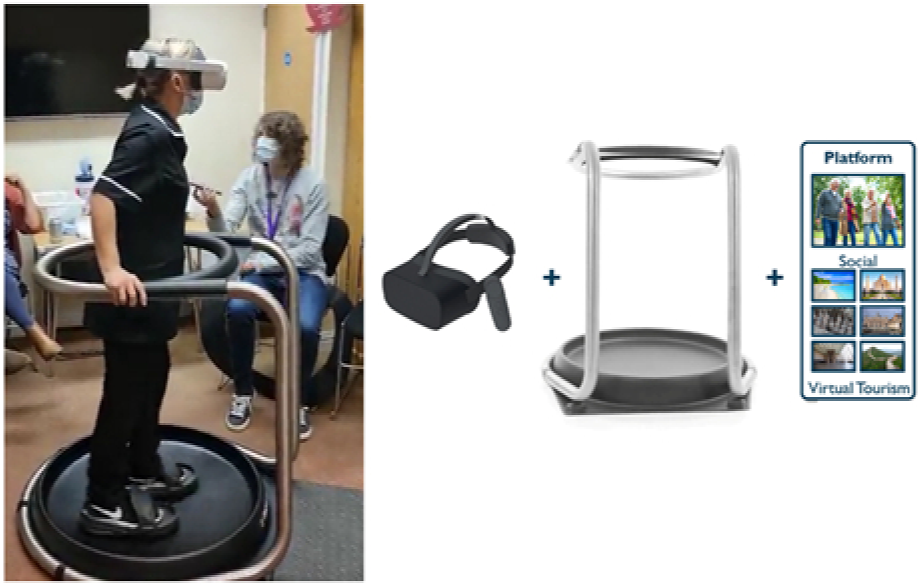

Figure 1: (Left) Care home staff member interacting with the ROVR Adventure treadmill with researcher recording initial reactions. (Right) VR headset with the ROVR Adventure omnidirectional treadmill and example of the app to access VR worlds

124x79mm (96 x 96 DPI)

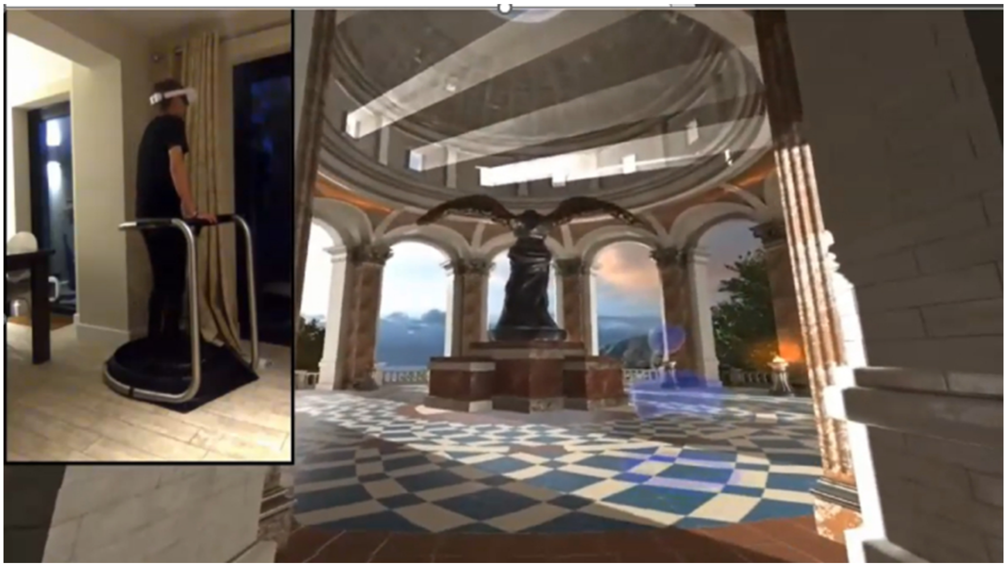

Figure 2: Example of the virtual museum participants could explore by walking on the ROVR Adventure

248x138mm (96 x 96 DPI)

Appendices

Appendix A –Workshop Interview Schedule

Site information

1) Please briefly describe the site you work at? (e.g. care home, extra care, supported living)

2) Please describe the number of residents or customers at that site and the level of care required? (e.g. physical ability of residents, prevalence of dementia)

Feedback on ROVR

1) What are your overall thoughts on the ROVR? How physically demanding did you find use of the ROVR?

2) What, if any, benefits do you perceive older adults might gain from using the ROVR?

3) What, if any, challenges might be encountered in the use of the standing or seated ROVR system with older adults (and care home residents)?

4) What characteristics do you think older people would need to use the standing and/or seated ROVR safely?

5) What challenges or health conditions do you think might make use of the standing and/or seated ROVR unsafe? (e.g. visual impairments, osteoarthritis, balance and coordination issues, frailty, mobility issues, support with grooming/communication/transfers/toileting/locomotion/self-care/communication *examples taken from the Functional Independence Measure*)

6) If the ROVR was in your care facility, do you envisage that it would change ways of working? (if so, how?)

7) Based on the video and demonstration you saw today, would you feel confident supporting use of the ROVR at your care facility? (further ideas on training requirements)

For peer review only

Appendix B – Supporting evidence for Table 2

Theme	Initial Codes, *Sub-codes*	Example Evidence
Anticipated benefits	Virtual visits	
	- *Interesting local sites*	"Eden project, Tintagel, Cornish mines, that kind of thing. That's the hope, and particularly post COVID with kind of reductions in mobility." (Care Home 1)
	- *Virtual tourist sites*	"Some of these tourist sites to visit. So you could go to Venice or Paris." (Workshop 1)
	Physical activity/exercise	"And it does exercise the top of the body as well. Yeah, obviously you need to push down. Yeah, take the weight off your feet when you slide. So it's, well, it is a whole body exercise really" (Care Home 5)<br>"You know, it's making each day have something new or useful or different, and this could open that to them. A massive, massive but as well as you know, without them realizing bit by bit and increasing their fitness, mobility, their longevity, their life span, their health" (Workshop 2)
	- *Motivating activity*	"And you know, struggle to get [exercise], how boring it is. If they can actually walk on that something to motivate them to do it. Yes, massive motivation isn't it. " (Care Home 5)
	Prevents boredom	"Oh, it's good exercise as well as Yeah, it's actually giving them an experience. It's getting a mobile, perhaps someone that might just sit in their chair normally." (Care Home 2)<br>"And [a resident] gets bored sometimes in the afternoon to the evening. And he's getting his coat or going out for a walk. Well, if we set him up on there. You could have a walk on here instead" (Care Home 5)
	Rehabilitation	"We had a bloke the other day that had a stroke. In the April he was discharged from hospital they couldn't do any more for him and he wasn't standing, wasn't walking. He went home from us because we, our staff were inputting exercise […] There was another lady […] But she was off her legs. And we've got her going and then she went home again. Yeah. If we can get close enough [to the ROVR] with the chair that they can actually sit and do it, it would be [rehabilitating]." (Care Home 5)<br>"Particularly I think it'd be fantastic for rehab. Absolutely. Brilliant. People go that have to walk up and down bars." (Care Home 5)
	Staff use	"Yeah. even if our staff will be using it, our staff in front of our residents, makes good health and wellbeing staff and a morale boost. Yeah, because it's something new, something exciting that they can all talk about. So it's not just not just yeah, not just the service users, the staff as well as the whole well being." (Care Home 5)
	Social interaction	"The novelty factor and the benefits of something different to what they've experienced in it, the talking points for themselves and their families that they probably can hang on to for quite a while." (Workshop 1)<br>"I'm anticipating, essentially, social interaction, with the carers a bit more because there's going to be more communication? Yeah. So enhanced communication. Yes. Exercise. Yes. Yeah. Exercise. And interesting life." (Care Home 5)<br>"[They can talk to each other] Yeah. I think that's the best part of it" (Care Home 2)<br>"So maybe in time, it would be nice to involve the communities in and around our houses." (Workshop 2)
	Wellbeing	"Ultimately, if they're using it, their well being is completely going to change and they're going to be happier." (Care Home 1)<br>"But I think for mental health it would be good" (Care Home 2)
	Accessible experiences	"So some are just not able to get out anymore. Yeah. So if you're providing the opportunity to see the place that they love" (Care Home 4)
	- *Different to care home*	"If you're living in a care home, to go to the different, that environment was lovely." (Workshop 1)
	Stimulates the brain	"Stimulates the brain. Yeah. Got me thinking." (Care Home 1)
	Restorative	"that will bring their confidence back" (Workshop 2)
	Reminiscence	"Especially if they're walking in an area they know. They are walking down. Trigger memories" (Care Home 2)

For peer review only

	- *Reminiscence benefits*	"Well, I think it's memories, it's reminiscing for them, isn't it? If they went to it's like, you know, bearing in mind not everybody that lives here is from Devon or Cornwall, we've got people that have moved from the land at some point. So even to have those places it brings back memories. You know, we've got old photographs of Saltash, pictures of Saltash, and that, you know, they'll say, Oh, we went to that cinema when I was young. You know, it brings back memories. So I'm presuming this will do the same. I'll show them because yeah, A lot of living with dementia. They're living years ago." (Care Home 4)
	Expectations challenges positively	"That was alright actually, I didn't think, it wasn't as bad as I thought it was going to be. I felt more in control than I thought I would." (Care Home 2)
	Positive distraction and novelty	"What we were saying, and you know you said how was things I think, you know, people at the moment are feeling so overwhelmed. Yeah, we've been through something like this would take their minds off things" (Care Home 2)
Acceptability	Positive comments	"Yeah, that's amazing. Absolutely amazing" (Care Home 3)<br>"I honestly think this is amazing" (Care Home 6)
	ROVR is interesting	"Interesting idea." (Workshop 1)
	Adoption desired	"I would […] buy it for the staff and residents. I would consider that, I would put it before my board so that we actually had one permanently." (Care Home 5).
	Ease of use	"Easy to control" (Care Home 5)
	- *Headset initially heavy but comfortable*	"It feels a little bit heavy forward so I've got to bring it forward, but it is good." (Workshop 1)
	- *Physically easy*	"Not for me intense at all." (Workshop 1)
	- *Treadmill is easy to enter*	"In felt fine [getting in and out], because at least I had one stable foot on the ground, and sort of the bars were in front of me." (Workshop 1)
	- *Treadmill is easy to move on*	"I actually find the walking on it much easier than I thought it would be to be." (Workshop 1)
	- *VR is novel experience*	"One [benefit], the novelty factor and the benefits of something different to what they've experienced in it." (Workshop 1)
Concerns of use	Secureness	"That does feel more secure, leaning on the bars around you. And it helps to keep you stable." (Care Home 3)
	Safety concerns	"Yeah, just whilst they still think they can walk. And while that seems pretty easy, yeah, that potentially is gonna make them really unstable. Or they'll get breathless really quick" (Care Home 3)<br>"It's unsafe I would say as strong as that they would be unsafe" (Care Home 4)
	- *Hitting objects*	"... Oh, I've got a move in a minute, because I'm going to hit, say the bollard, I already hit it. And I wanted to go up the stairs. But I couldn't go upstairs because I'd hit it already..." (Care Home 1)
	- *Shoes*	"The shoes are going to be a little bit of an issue. If they can come in different sizes that might be But the smaller they are, the tighter they're going to be. So I found that quite tight to fit the one large and I've only got size three feet. Yeah. very slippery to stand on. And I can see that being quite an issue" (Care Home 1)
	- *Safety hazards*	"Maybe you need something around the outside. So if they fell, they just they can't come out. They can only go on their knees then can't they?. Do you know what I mean? If there was a guard? Actually, because that's my worry if someone fell out of that." (Care Home 5)<br>"You see that wouldn't be suitable for a resident. That is too slippery" (Care Home 4)<br>"That's that's a slippery slope." (Workshop 2)
	- *Danger*	"They [residents] can't afford to fall, if they fall it's gonna be an injury isn't it" (Care Home 6)<br>"It's a bit slippery to get on and off? That is a danger" (Care Home 5)
	Lack of control	"It's it's it's fast and then it's because you can't control it" (Care Home 3)

For peer review only

For peer review only - http://bmjopen.bmj.com/site/about/guidelines.xhtml

	- *Movement issues*	"I'm not really moving anywhere." (Workshop 1)
	Risk of falls/Dangerous	"more support for them because the type of people that we get like I've said are not as agile as they used to be even though sometimes they think they are and they're potentially going to fall in that." (Care Home 3)
	- *History of falls*	"I don't know that she'd be happy to do it. Because she's got a history of falls. Okay, so she'd probably not enjoy it." (Workshop 1)
	- *Could cause falls*	"I think when it could actually make someone fall." (Workshop 1)
	- *Risk of injury – hips*	"If you slipped now, you're gonna bang yourself on those bars, then you're gonna catch your ribs or whatever? Or your arm, break an arm on there if you go down, yeah." (Care Home 5)<br>"Yep. Okay. I can see a few hips going." (Care Home 2)
	Accessibility	"Getting in was alright, getting out was difficult, because you've already done that slippery surface" (Care Home 3)<br>"You could do with like a virtual reality wheelchair, they sit down and go, around." (Care Home 5)
	Prefer people go out	"the deterioration that we've seen in so many of our residents is tenfold and I'd rather get them out. But then you have that knock on effect that the families don't want to. But if they have the option of liaising via a website, let's go and visit Eden with her" (Care Home 1)
	Heavy shoes	"Yeah, the heaviness of those shoes, and being nervous of falling." (Workshop 1)
	Lack of motivation	"Definitely. Yeah. There's lots of 'Mister, come on, you can do it. You can do it.', 'I don't want to do it'." (Workshop 1)
	Uncomfortable movement	"I feel like the twisting itself, which is where the uncomfortable sensation perhaps maybe lies." (Workshop 1)
	Bigger environment more physically demanding	"Depends on how big the world is, because this world feels small and so I feel like I have to twist." (Workshop 1)
	Critique	"I wouldn't want them using it without a staff member on the site." (Workshop 2)<br>"Resident would be screaming the place down, and panic about the step [into ROVR]" (Care Home 6)
	Potential issues	"this idea of being in an open space or field might be disorientating" (Workshop 2)
Concerns of negative effects	Side effects	"Yeah. It wasn't motion sickness but I just started to feel like I just need to take this off now, yeah, it's just a really strange feeling" (Care Home 3)<br>"Yeah, I've got a feeling of like, yeah, maybe I've got like a bit of car sickness." (Care Home 4)
	- *Encouraging shuffling/sliding*	"That is almost the encouraging of Parkinson's type shuffling isn't it?" (Workshop 1)
	- *Unnatural movement*	"As you move around this change of texture to this and that makes it seem like it's not as organic or not as natural" (Workshop 1)<br>"A bit weird, because you're not stepping" (Care Home 5)<br>"Keep sliding […] it'll feel a bit weird" (Care Home 6)
	Dizziness	"I might feel dizzy. It was alright there but when you go off the steps and stuff you start getting a bit. It's because when you went the faster you go. Then when you turn that's when you get a bit. Isn't it like discombobulated?" (Care Home 5)<br>"It feels really weird, my head is still spinning" (Care Home 6)
	Disorientating	"That's amazing but it messes with your head" (Care Home 3)<br>"Trying to orientate yourself is quite difficult. Yeah. Yeah. Especially around the house." (Care Home 1)<br>"Especially when you got the headset on, you're disorientated as well." (Care Home 4)
	- *Orientating to environment*	"The only thing that concerns me is the disorientation that. Yeah, that's the only thing. Okay, Whether they'd be disorientated." (Care Home 2)<br>"Yeah, I think it'd be really hard because you lose all your visual cues like even though I've got no balance problems it was pretty tricky. You do certainly do lose your orientation before you put those on, like, I didn't know where anyone was. Deal with the disorientation and the

For peer review only - http://bmjopen.bmj.com/site/about/guidelines.xhtml

		nausea. Anyway, which they might not be able to tell you about, so I'd be worried about it for dementia. Without a lot of, it's worth trying." (Workshop 1)
	- *Coordination between VR and feet*	"Yeah, since you've had the headset on your feet don't seem in momentum. Is it difficult to watch and organise your feet?" (Care Home 4)
	- *Height changes*	"It [*disorientation*] didn't affect me till there was like the change in height" (Workshop 1)
	- *Motion*	"Motion is kind of backwards and forwards, like you're on a cross trainer." (Workshop 1)
	- *Social angle confusing*	"For a few? Most of them I would say it [social experience in VR] would be very confusing." (Workshop 1)
	Concentrating more on the exercise	"Concentrating more on the exercise." (Workshop 1)
	Depth perception difficulties	"Because it is such a such a strange sensation of a lot of different coordination at the same time, and that the depth perception issues" (Workshop 1)
	Motion sickness in VR	"Oh, I'm actually feeling really dizzy." (Workshop 1)<br>"I felt motion sickness when I got off yeah. But I think it was too enclosed. Like, there's too many walls and things. I hit a wall quite quickly," (Care Home 4)
	Fear	"I think some elderly people would find it quite scary. Yeah, I do." (Care Home 1)<br>"They [residents] might feel a bit scared, because because it's slippy." (Care Home 2)
Suitability/Unsuitability	Extra support	"I think the worry for me is is if we get to the stage where the residents go in there. Yeah, I think we need to have much more support for them" (Care Home 3)<br>"I don't think I'd feel happy start with if they weren't being supervised." (Care Home 2)
	- *Supervised use required*	"Yeah, but you still need to hold on it. But I think what you would always do with the residents on that, or you would always have one person here" (Care Home 5)
	- *Guidance and guided navigation*	"I was just saying, if your staff can't see what's going on, when you run into problems, they can't troubleshoot it for someone who's struggling cognitively, or even just unfamiliar with it, like, am I on the balcony? Do I need to get to it? If I'm not on the balcony, how do you guide someone up to it? What do I do? They can't then put the mask on, with infection, you can just put the mask on and check, you've got to take someone's headset off." (Workshop 1)
	- *Human resources*	"I do think it will need some supervision, so definitely, we're going to have to get the staff on board with it. Because well, I, it's not something that I would expect to walk in at nine o'clock in the morning and see someone on it without one of us being there" (Workshop 2)
	- Number of residents	"they all they've all got walking frames and need wheelchairs. And even that's residential. I would say only a couple." (Care Home 1)<br>"I can think of one called [resident name]. Yeah, that's about the only one I will allow would feel comfortable with." (Care Home 4)<br>"Obviously you just have to pick the residents that would be appropriate for, because someone with mental health issues would really struggle on this." (Care Home 4)
	- *Differences between residents*	"[Suitable for residents with] More stability when they're on their feet" (Care Home 5)
	- *Residents unable to use*	"I don't know if a resident would be able to do that" (Care Home 1)
	Diagnosis	"Imagine that somebody with cognitive impairment or a spatial awareness issue, poor eyesight, mobility wise, […] not somebody of age (Care Home 6)

For peer review only - http://bmjopen.bmj.com/site/about/guidelines.xhtml

	- *Balance*	"I think it's the overall, you know, they are on lots of medication, how that's going to affect their balance. I've got some people who have vertigo, they wouldn't be able to do it." (Care Home 1)
	- *Dementia/cognition*	"First, immediately, people are just gonna let go. Okay, especially if they got mild dementia and something like that" (Care Home 1)<br>"And of course they are in a not in a reality situation. So they don't know actually where they are." (Care Home 5)<br>"Has anyone said about the issue about with the mental health. They might think why are here? Yeah. Why are we keeping them from them. There's some here that would love to be in Australia with the granddaughter and they would fully understand that it's a Virtual world and also coming back out and leaving that experience then suddenly the grandchild is gone again. Someone living with dementia only got to see somebody crossing the car park, and they think it's one of their family and they think why do you not let them in, it's not their family but they think." (Care Home 4)<br>"I feel like it would totally freak our clients [...] I think better cognition" (Care Home 6)
	- *Fear of falling*	*Interviewer:* "Yeah. Do you think that that slipperiness would be a problem? "<br>*Participant:* "It would be for some of our residents here. Yeah. Yeah. They would just fall over" (Care Home 1)<br>"Just what we've already said, totally inappropriate for residents because if they fall." (Care Home 4)
	- *Have to be able*	"They've got to be quite able. So our most able person is is she's done the, the other one with us. She goes out gardening, she goes out doing lots of things. But she's had a fall, because she was pulling weeds out and one was too strong. So it can easily be done. That then will knock her confidence. So I don't want her going on something like this that she wasn't that keen. Yeah. Okay. So they have to be very able" (Care Home 1)<br>"In fact, they because I've had to hold here. They would have to hold on really tight. Yeah. And because they're older this the grip alone." (Care Home 4)
	- *Incontinence*	"So you might have to look around that a little bit. Yeah. Especially with people that are already afraid of falling. Yeah, they're incontinent because they fall. They don't want you know, they don't want to go to the toilet, because so going on something like this might be a little bit of an issue." (Care Home 1)
	- *Influence of medication*	"I think it's the overall, you know, they are on lots of medication, how that's going to affect their balance. I've got some people who have vertigo, they wouldn't be able to do it" (Care Home 1)
	- *Limited mobility*	"This bit as well. I mean, we've got here they're not very mobile or anything." (Care Home 1)<br>"We're got a few that are quite mobile. Even the ones that could walk I think would really struggle." (Care Home 5)
	- *Problems with vision*	"A little bit. Getting better but it's still quite blurry though." (Care Home 4)
	- *Neck pain*	"I was like that well, going around rather than like that, that would that give you the neck neck ache and then that, okay, posture? Yeah" (Care Home 1)
	- *Neuromuscular*	*Interviewer*: "So thinking about maybe some of your family members, then what kind of conditions or challenges do you think a person would have that would mean this wasn't suitable?"<br>*Participant:* "Neuromuscular degeneration is one for me." (Workshop 1)
	- *Lived problem*	"would that be important that actually there was an ongoing service? Rather than you buy this and you're left on your own? Would you prefer it that actually, perhaps I pay a monthly fee? And I know, I can always pick up the phone if something goes wrong and contact that company? Would you rather kind of buy it and own it? Or have that kind of that long term rental maintenance and the service that comes with it?" (Workshop 2)

For peer review only - http://bmjopen.bmj.com/site/about/guidelines.xhtml

	- *Vertigo*	“For someone with that impairment, or someone with that illness, that's just not suitable for them. What about someone with vertigo?” (Care Home 2)
	Suitability for residents	“What do you think with the residents? I just I just said there's not many that we'll be able to put on that.” (Care Home 5)
	- *99% not suitable*	“99% won't be able to do it anyhow” (Workshop 1)
	- *Suitable for residents 20 years younger*	“Twenty years ago when we had residents, like you can think we still got residents that would have been fine but now. No way” (Workshop 1)
	- *Wouldn’t recommend to people with mobility issues*	“Unsteady gait in general, having vertigo, history of, history of falls in any sense, increasing that risk of. I wouldn't bother I think.” (Workshop 1)
	Ethics	“There's still challenges, though, isn't there of getting on and off like exercise, you know, I've taken loads of people on it, like that, it can be quite a dodgy thing to do. It's not okay, so yeah, there's still challenges with getting older adults on and off these things, especially anyone who has cognitive impairment,” (Workshop 1)
	Risk	“Older adults wouldn't be able to even step over that even though she's on it […] And probably in nursing homes older people's standing tolerance may not be that. And the effect on balance from turning, as well as the nausea point of view” (Workshop 1)
	Size of technology	“It is massive. I didn't expect it to be this big.” (Care Home 1)
	- *Wifi connectivity*	“It would probably be in the lounge because probably we get quite good reception in the lounge. Sometimes in parts of the building we might lose it at times.” (Care Home 4)
	Familiarisation	“And just like anything else, it's time to get used to it. That familiarization with. But that's like everything that we have that's new. Once people are familiar with it, and it's normal. I mean, to our residents, this sort of technology is quite strange, anyway,” (Care Home 2)
	- *Adapting to use*	“Because I'm, I've got a bad back. But I was able to still able to do it. I got poor eyes because I've got wet macular degenerate disease but it was fine. Okay, I couldn't do as long as what [other participants] did probably, but also it's my first time in VR so I was cautious but as you get used to it.” (Care Home 5)
	- *Unfamiliar*	“I can just see a pillar here. I could hear someone it's like the pillars talking […] It's like I'm disorientated” (Care Home 5)
	- *Adjusting to headset*	“And when the VR went on, very disorientating, very blurry, and for then to adjust it as they want it, I think might be an issue. So you need to be able to see what they're seeing on the computer to you maybe have to adjust it that” (Care Home 1)
	- *Familiarity*	“I certainly think the fact that you're going to have destinations, local destinations will be the attraction.” (Workshop 2)
Improvements	Content suggestions	“Under the sea would be good wouldn't it?” (Care Home 5)<br>“We could take them to places. If we could get in the gardens every year. If you could capture that.” (Care Home 4)<br>“Because you really you're taking them to places you know like the Beaches.”(Care Home 4)
	- *Cartoon-like images*	“You want to touch the flowers, so they will let go and then they'll Yeah, yeah. So I do think that's a bit of a challenge mobility, and that's quite cartoonish as well. So will it be reality?” (Care Home 1)
	- *New environments*	“Yeah, Mount Edgecumbe. All those kinds of things that they they would love. Even the Tamar Bridge. A garden center. You know, last gardens of Helligen, Eden Project. I think they'd love it. Yeah, I think they'd really love it. And they're sitting down and they can look at the flowers. And, you know, pick, they can do all these kinds of things. But if they're standing up, I think there'll be more "I don't like it. I don't like it", because it's do you see what I mean? Yes. Yeah,” (Care Home 1)
	Inclusivity	“how we could adapt the equipment for her typically she has full capacity and would really benefit from this but obviously it's a double amputee so she's not going to physically walk” (Care Home 3)
	- *Adaptable bar height*	“Yeah, even if that was that was, you could undo it and take that bit off. You only need an eye and a button don't you like you don't like the walking sticks with the eyes and the button you only need a click button and then you could make it different sizes as well. It could go back further, be adaptable. They have something to hold on to in front of them.” (Care Home 5)

For peer review only - http://bmjopen.bmj.com/site/about/guidelines.xhtml

	- *Fitting to a wheelchair*	"Even if it's just like a normal chair. Yeah. Not a wheelchair. Because when you soon as you get in a wheelchair, you think you're going somewhere, which might be a good thing." (Care Home 1)
	- *People less mobile*	"You know, most of them come in in wheelchairs. Okay. That's the way residents are coming in." (Workshop 1)
	- *Access could be improved*	"You have to naturally be able to step up, don't you? So if you could step up onto like, just a something there, as a gap in between." (Workshop 1)
	- *Physical requirements*	"From a physical perspective. I have to say this is a dance of what they have been used to before, but I would think that whoever would need to, to safely do this would already have a degree of practicality about them." (Workshop 1)
	Safety improvements	"There's that feeling of going back. So I think if there were two front legs and at the back, that would give much more support?" (Care Home 3)<br>"I think you're looking at someone with a degenerative physical disease, but good cognition, once you've made some modification, so it doesn't kill them." (Workshop 1)
	- *Softer impact bars*	"If they do, if a resident slips out, as in legs come down. Obviously, we would need some sort of buffers over the top of the bars," (Care Home 5)
	- *Stop slipping*	"I don't know, do you need to have it slippery actually." (Care Home 5)
	- *Awfully slippery*	"Awfully slippery. So you're, you're like when you're walking on ice or wherever you're on guard. So that takes away the enjoyment." (Workshop 1)
	- *Difficulty exiting treadmill*	"I'm not sure how many people would be able to get out of that." (Workshop 1)
	Add arm movements	"You have like any interaction with hand stuff as well. You know you've got your feet Yeah, you do. So you have things in your hands so" (Care Home 5)
	Cast visual for group to watch	"Yeah. I don't know if we could connect to this screen, so the rest of them can actually see on the screen where this [in the VR] is." (Care Home 5)
	Seated version	"The seated unit would be more beneficial to our client base, as we are at the moment." (Care Home 2)<br>"It would have to be a sitting one, but would they like that [VR headset] on their head" (Care Home 6)
	- *Square*	"If it was sitting down. Yeah. Yeah, that would be different." (Workshop 1)
	- *Headset use only*	"I would think if it was less slippery, yeah. And maybe if you could, well, you couldn't just use the headset. Could you? Could you just use the headset?" (Care Home 1)
	- *Adaptations*	"Could they not sit? If they were sat using their feet Yeah. But then they wouldn't get do you have to. Is there a was a chair scenario." (Care Home 4)
	Possibilities/exercise bike	"I wonder about it on a bike because that way. You've got your bike, which turns, so pedalling is innate. People know peddling goes forwards. And so on. And if you could turn on that. I wonder if it would keep you a little bit more orientated? Because all your movement has been done with that and with the steering wheel aspect to that. And so I don't know if that would be better. If you're trying to get the exercise. Cause it's really interesting. You're looking at it from the exercise point of view. If it's just getting someone out and about." (Care Home 5)
Current design	Good content	"I'm just getting to the balcony. Oh my god. Oh that is amazing brilliant. It is amazing that is phenomenal." (Care Home 5)<br>"But it just feels so real" (Care Home 4)<br>"It's beautiful" (Care Home 6)
	- *Visual features*<br>- *Spatial features*	"No, I don't want to go down the steps. I'm gonna go this way. I don't want to go down the steps, I don't like it" (Care Home 4)
	Liked headset	"It doesn't feel heavy on my head" (Care Home 5)

For peer review only - http://bmjopen.bmj.com/site/about/guidelines.xhtml

		"Yeah, that's good, it feels really comfortable" (Care Home 4)
	Safety features	"That's obviously why the bars are there" (Care Home 5)
	Technology issues	"However much walking I got I wasn't going anywhere." (Care Home 5)
	- *Glitching*	"You're actually flying all over the place […] you are there but you're flashing all over it as well." (Care Home 5)
	- *Loading worlds*	"All I got is clouds and a green line. It's just a green line." (Care Home 5)
	- *Speed of movement*	"I'm guessing I would say that one slide of that foot? You'd probably moving about two feet." (Care Home 5)
	Ergonomics	"I'm stepping in a way I wouldn't normally step" (Care Home 5)<br>"They didn't feel big on the feet. Because you're sliding" (Care Home 4)
	Mechanics	"It doesn't feel like natural walking." (Workshop 2)
	Resolution	"it's going to feel a lot more stable with that in there, and so they've suggested that when people have their first experiences, it's always with the training mats in place." (Workshop 2)

For peer review only - http://bmjopen.bmj.com/site/about/guidelines.xhtml

For peer review only

For peer review only - http://bmjopen.bmj.com/site/about/guidelines.xhtml

COREQ (COnsolidated criteria for REporting Qualitative research) Checklist

A checklist of items that should be included in reports of qualitative research. You must report the page number in your manuscript where you consider each of the items listed in this checklist. If you have not included this information, either revise your manuscript accordingly before submitting or note N/A.

Topic	Item No.	Guide Questions/Description	Reported on Page No.
**Domain 1: Research team and reflexivity**			
Personal characteristics			
Interviewer/facilitator	1	Which author/s conducted the interview or focus group?	
Credentials	2	What were the researcher's credentials? E.g. PhD, MD	
Occupation	3	What was their occupation at the time of the study?	
Gender	4	Was the researcher male or female?	
Experience and training	5	What experience or training did the researcher have?	
Relationship with participants			
Relationship established	6	Was a relationship established prior to study commencement?	
Participant knowledge of the interviewer	7	What did the participants know about the researcher? e.g. personal goals, reasons for doing the research	
Interviewer characteristics	8	What characteristics were reported about the inter viewer/facilitator? e.g. Bias, assumptions, reasons and interests in the research topic	
**Domain 2: Study design**			
Theoretical framework			
Methodological orientation and Theory	9	What methodological orientation was stated to underpin the study? e.g. grounded theory, discourse analysis, ethnography, phenomenology, content analysis	
Participant selection			
Sampling	10	How were participants selected? e.g. purposive, convenience, consecutive, snowball	
Method of approach	11	How were participants approached? e.g. face-to-face, telephone, mail, email	
Sample size	12	How many participants were in the study?	
Non-participation	13	How many people refused to participate or dropped out? Reasons?	
Setting			
Setting of data collection	14	Where was the data collected? e.g. home, clinic, workplace	
Presence of non-participants	15	Was anyone else present besides the participants and researchers?	
Description of sample	16	What are the important characteristics of the sample? e.g. demographic data, date	
Data collection			
Interview guide	17	Were questions, prompts, guides provided by the authors? Was it pilot tested?	
Repeat interviews	18	Were repeat inter views carried out? If yes, how many?	
Audio/visual recording	19	Did the research use audio or visual recording to collect the data?	
Field notes	20	Were field notes made during and/or after the inter view or focus group?	
Duration	21	What was the duration of the inter views or focus group?	
Data saturation	22	Was data saturation discussed?	
Transcripts returned	23	Were transcripts returned to participants for comment and/or	

For peer review only

Topic	Item No.	Guide Questions/Description	Reported on Page No.
		correction?	
**Domain 3: analysis and findings**			
Data analysis			
Number of data coders	24	How many data coders coded the data?	
Description of the coding tree	25	Did authors provide a description of the coding tree?	
Derivation of themes	26	Were themes identified in advance or derived from the data?	
Software	27	What software, if applicable, was used to manage the data?	
Participant checking	28	Did participants provide feedback on the findings?	
Reporting			
Quotations presented	29	Were participant quotations presented to illustrate the themes/findings? Was each quotation identified? e.g. participant number	
Data and findings consistent	30	Was there consistency between the data presented and the findings?	
Clarity of major themes	31	Were major themes clearly presented in the findings?	
Clarity of minor themes	32	Is there a description of diverse cases or discussion of minor themes?	

Developed from: Tong A, Sainsbury P, Craig J. Consolidated criteria for reporting qualitative research (COREQ): a 32-item checklist for interviews and focus groups. *International Journal for Quality in Health Care*. 2007. Volume 19, Number 6: pp. 349 – 357

Once you have completed this checklist, please save a copy and upload it as part of your submission. DO NOT include this checklist as part of the main manuscript document. It must be uploaded as a separate file.

For peer review only
